# High Resolution NMR Spectroscopy as a Structural and Analytical Tool for Unsaturated Lipids in Solution

**DOI:** 10.3390/molecules22101663

**Published:** 2017-10-05

**Authors:** Eleni Alexandri, Raheel Ahmed, Hina Siddiqui, Muhammad I. Choudhary, Constantinos G. Tsiafoulis, Ioannis P. Gerothanassis

**Affiliations:** 1 Section of Organic Chemistry and Biochemistry, Department of Chemistry, University of Ioannina, GR-45110 Ioannina, Greece; alexandri_e@hotmail.com; 2 H.E.J. Research Institute of Chemistry, International Center for Chemical and Biological Sciences, University of Karachi, Karachi 75270, Pakistan; genius88raheel@gmail.com (R.A.); hinahej@gmail.com (H.S.); 3 Department of Biochemistry, Faculty of Science, King Abdulaziz University, Jeddah 214412, Saudi Arabia; iqbal.choudhary@iccs.edu; 4 NMR Center, University of Ioannina, GR-45110 Ioannina, Greece; ctsiafou@cc.uoi.gr

**Keywords:** unsaturated lipids, polyunsaturated fatty acids (PUFAs), NMR, chemical shifts, coupling constants

## Abstract

Mono- and polyunsaturated lipids are widely distributed in Nature, and are structurally and functionally a diverse class of molecules with a variety of physicochemical, biological, medicinal and nutritional properties. High resolution NMR spectroscopic techniques including ^1^H-, ^13^C- and ^31^P-NMR have been successfully employed as a structural and analytical tool for unsaturated lipids. The objective of this review article is to provide: (i) an overview of the critical ^1^H-, ^13^C- and ^31^P-NMR parameters for structural and analytical investigations; (ii) an overview of various 1D and 2D NMR techniques that have been used for resonance assignments; (iii) selected analytical and structural studies with emphasis in the identification of major and minor unsaturated fatty acids in complex lipid extracts without the need for the isolation of the individual components; (iv) selected investigations of oxidation products of lipids; (v) applications in the emerging field of lipidomics; (vi) studies of protein-lipid interactions at a molecular level; (vii) practical considerations and (viii) an overview of future developments in the field.

## 1. Introduction

**Figure 1:**
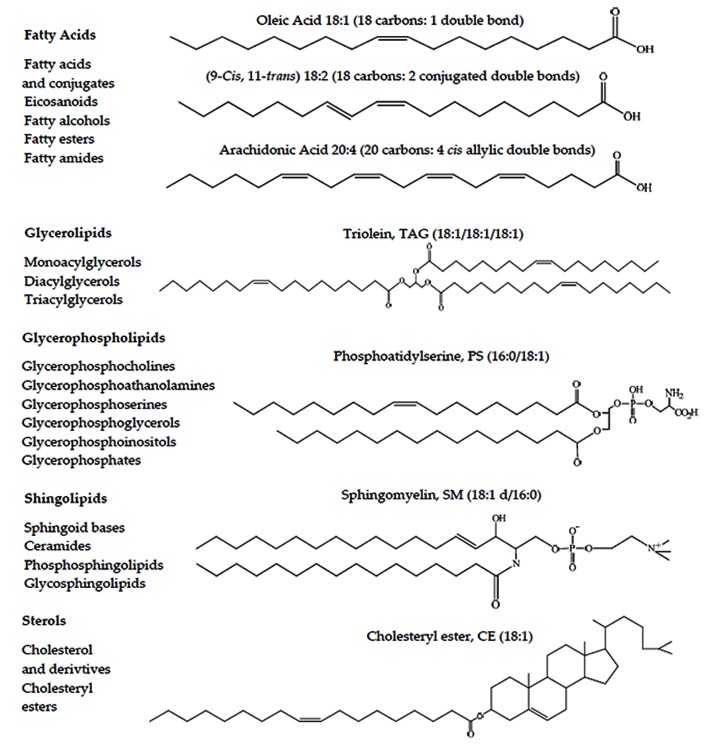
Representative structures of various classes of lipids. Adopted, with permission, from [14]. Copyright 2012, Wiley VCH.

Lipids are a broad and diverse group of naturally occurring biomolecules with numerous physicochemical and biological properties. They play diverse and important roles in nutrition and health [1,2,3,4]. Lipids have important functional roles as major constituents of cellular membranes (e.g., brain consists mainly of fats [4]), signaling molecules, and as energy source for metabolic processes. Lipids due to their hydrophobic chemical structures are incompatible with an aqueous medium, therefore, their circulation through the blood stream to reach peripheral tissues is achieved through the formation of macromolecular complexes with lipoproteins. Fatty acids (FAs) are the major lipid building blocks of complex lipids, such as glycerolipids, i.e., monoacylglycerols (MAGs), diacylglycerols (DAGs), and triacylglycerols (TAGs, Figure 1). These neutral lipids have a glycerol backbone with FA chains attached to the glycerol group, most commonly with an ester bond, but ether bonded FAs can be also found in minor amounts.

Lipids are widely distributed in plants and animals. In plants, they are found in seeds (e.g., sunflower, papeseed, cottonseed and soybean), fruits (e.g., olive, palm fruit and avocado), and nuts (e.g., almonds and walnuts). Common animal lipid sources are meat, fish, eggs and milk. Monounsaturated lipids are widely distributed in olives, peanuts, canola and other vegetable oils. Polyunsaturated lipids are found in various seed oils and sea food. The degree of unsaturation of various lipids has raised considerable research interest in the last decades, since dietary oils containing saturated fatty acids were shown to elevate total and LDL cholesterol and, thereby, increase the risk for cardiovascular diseases [5]. On the contrary, oils which contain polyunsaturated lipids lower the plasma lipid levels [5,6,7]. Health concerns have also been raised about the *trans* fatty acids that may be present in processed oils as a result of isomerization of the *cis* double bonds during refining and deodorization processes [8,9]. Health organizations, therefore, have recommended limited dietary intake of *trans* fatty acids [10,11] and for food products which contain fats, the nutritional labelling should include specific information about the content in *trans*, saturated, mono- and polyunsaturated fatty acids [12].

The nomenclature of unsaturated fatty acids (UFAs) derives from the name of the parent hydrocarbon with the suffix enoic acid [13]. The numbering of carbon atoms in FAs (Table 1 and Table 2) begins at the carbonyl terminus and the end methyl carbon is known as omega (ω) or *n* carbon atom. Thus, linoleic acid is a ω-6 (*n*-6) fatty acid because the second double bond is 6 carbons from the methyl end of the molecule (i.e., between C-12 and C-13 from the carbonyl end). Various conventions are adopted for indicating the positions of the double bonds. The most widely used is the symbol delta (Δ) followed by the superscript number. For example Δ^9^ means that there is a double bond between C-9 and C-10. The total number of carbon atoms and number of position(s) of double bond(s) is again indicated by convention. Thus, the symbol 18:2; 9, 12 denotes a C-18 fatty acid with two double bonds between C-9 and C-10 and between C-12 and C-13.

**Figure 2:**
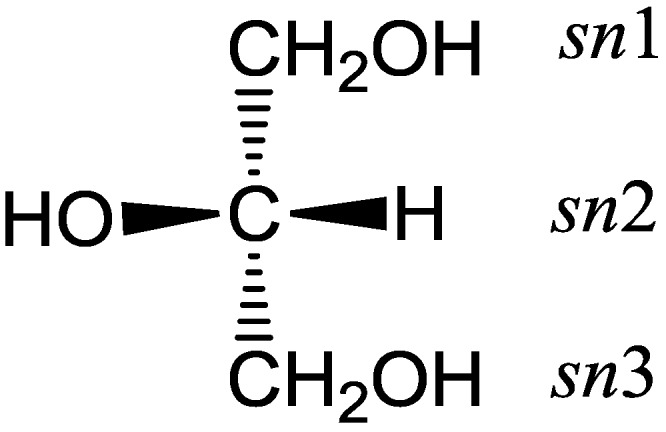
Stereospecific numbering of *sn* of triacylglycerols. Adopted, with permission, from [13]. Copyright 2002, Marcel Dekker, Inc.

Analytical and structural determination of unsaturated lipids is challenging, due to their physicochemical, biological, and nutritional diversity, markedly different polarities and the lack of chromophores that might facilitate spectrophotometric detection. Furthermore, when the acyl chain of the glycerol hydroxyl groups C-1 and C-3 are different or when one of the primary hydroxyls is esterified, the central carbon atom becomes a chiral center and enantiomeric configurations should be identified (Figure 2). Therefore, highly sensitive and selective analytical and structural methods are required for comprehensive study of this class of lipids [14,15]. Methods widely used include gas chromatography (GC), GC-mass spectrometry (GC-MS), GC-tandem mass spectrometry (MS/MS), LC-UV, liquid chromatography-mass spectrometry (LC-MS), and LC-MS/MS. The most powerful tool for analysis of lipid mediators is LC-MS/MS due to its specificity and high sensitivity [14,15]. However, GC methods involve several manipulation and derivatization steps that may cause oxidation of lipids [16,17], variable results depending on the *trans*-methylaction method and columns used [16,17] and undesirable isomerization processes [18].

High resolution nuclear magnetic resonance (NMR) spectroscopy has become a widely utilized method in lipid analysis. It allows structure elucidation, and qualitative and quantitative analysis of lipid molecules even in complex mixtures. Compared to common chromatographic analysis (e.g., GC and HPLC), NMR spectroscopy has several advantages. NMR is a non-destructive method and, for quantification, no specific standards are necessary. However, NMR techniques have only low to moderate sensitivity, compared to MS. Moreover, NMR-based lipid-analytical and structural methods are typically limited by overlapping signals in the ^1^H-NMR spectrum and the low natural abundance of ^13^C. Nevertheless, with the increasing availability of high field instrumentation, high sensitivity cryogenic probes and micro coil technology, advanced data processing and the great variety of multinuclear 1D and 2D NMR techniques, high-resolution NMR spectroscopy has become increasingly popular in the study of lipids. Readers should consult relevant review articles and books [19,20,21,22,23,24,25,26,27,28,29].

The objective of this review article is to provide a synopsis of the pleiotropic roles of unsaturated fatty acids and an overview of the critical ^1^H-, ^13^C- and ^31^P-NMR parameters for structural and analytical investigations and of the various 1D and 2D NMR techniques that can be utilised for resonance assignments. Several selected analytical and structural studies will be provided with emphasis in the identification of major and minor polyunsaturated fatty acids in complex lipid extracts without the need for the isolation of the individual components, investigations of oxidation products of lipids, applications in the emerging field of lipidomics and of protein-lipid interactions. A synopsis of practical aspects and future developments in the field will, also, be provided.

## 2. The Pleiotropic Role of Unsaturated Fatty Acids

Unsaturated fatty acids (UFAs) exert their physiological effects from specific locations in phospholipid pools in which they can be esterified. The movement of UFAs into phospholipid pools is catalyzed by fatty acid carrier proteins [30]. The acyl compositions of membranes and specific phospholipid pools are mediated by the specification of these enzymes. Two common functions of unsaturated fatty acids are protein-lipid interactions for enzyme targeting and messenger signaling [31]. A protein domain can recognize a membrane lipid and be targeted to a specific location in the cell for specific protein function. Lipids may also act as signaling molecules. For example, diacyglycerol (DAG) is a lipid second messenger that can modulate the function of protein kinase C (PKC) [32], while lysophosphatidic acid (PLA) is involved in the regulation of cell proliferation, migration and survival [33].

There is considerable evidence that the unsaturated fatty content of membranes can modify the structure and functionality of membrane enzymes [34]. Unsaturated fatty acids into membranes are confined, with few exceptions, in the *sn*-2 position of phospholipids (Figure 2). The occurrence of unsaturated and saturated fatty acids in the same phospholipid molecule can have important implications in membrane structure and function. First, the two types of acyl chains cannot demix in the membrane. Second, high degree of *cis* unsaturation implies less ability for rotation around the double bonds and, thus, greatly influences membrane acyl packing. Third, the position of the *cis* double bonds can play a role in the modification of the membrane structures. In contrast, the structures of *trans* fatty acids are relatively unaffected by the double bond position.

**Figure 3:**
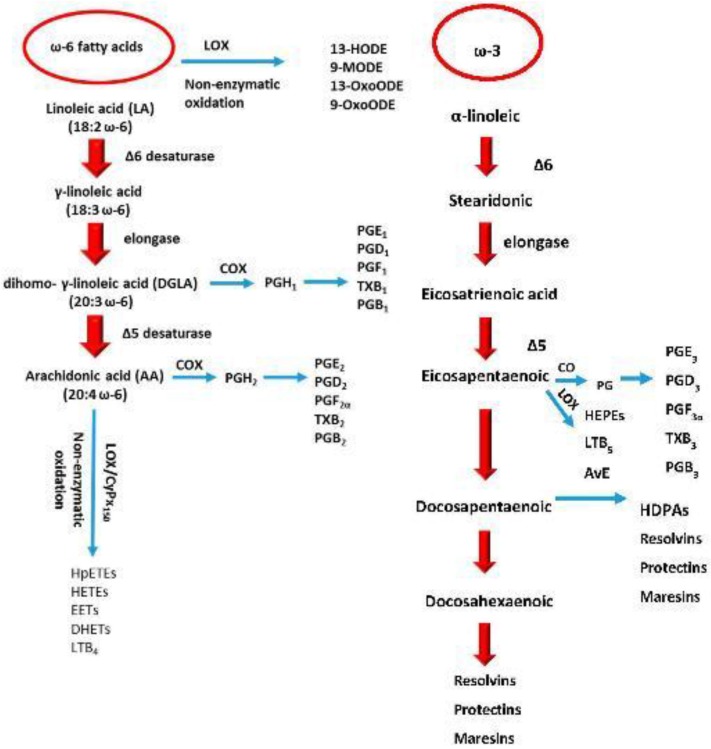
Schematic overview of ω-3 and ω-6 PUFA-derived lipid mediators. Adopted, with permission, from [35]. Copyright 2014, Springer International Publishing, AG.

Omega-6 and omega-3 polyunsaturated fatty acids (PUFAs), are essential fatty acids because they cannot be synthesized from simple precursors in the diet. They are precursors of biologically active lipid mediators such as eicosanoids including prostaglandins (PG), leukotrienes (LT), hydroxy eicosapentaenoic acid (HEPE), hydroxy eicosatetraenoic acid (HETE), epoxyeicosatrienoic acid (EET), and thromboxanes (TX) (Figure 3). They are a diverse class of bioactive lipid mediators derived from arachidonic acid (AA, 20:4ω-6) and eicosapentaenoic acid (EPA, 20:5ω-3) via cyclooxygenase (COX), lipoxygenase (LOX), and cytochrome P450 (CYP450) as well as nonenzymatic pathways. Lipid mediators are also produced from linoleic acid (e.g., hydroxyoctadecadienoic acid [HODE] and oxooctadecadienoic acid [KODE]) via the 15-LOX or non-enzymatic pathway. Other potent anti-inflammatory metabolites such as resolvins, protectins, and maresins are produced within the same pathway from EPA, docosapentaenoic acid (DPA, 22:5ω-3), and docosahexaenoic acid (DHA, 22:6ω-3).

It has recently been demonstrated [36,37] that several key actions of ω-3 fatty acids are mediated by two G-protein-coupled receptors (GPCRs) in the free fatty acid receptor (FFAR) family, FFA1 (GPR40) and FFA4 (GPR120). This is a rather strong evidence supporting preventative effects of ω-3 fatty acid consumption on several human cancers [38] including breast cancer [39].

The effects of ω-3 fatty acids on cardiometabolic risk factors, such as obesity, insulin resistance and dyslipidaemia are widely reported and debated in the literature. It has been postulated that the changing ratio of ω-3 vs. ω-6 fatty acids has affected physiology and that humans may have developed major classes of pathologies such as coronary artery disease, cancer and autoimmunity [40,41,42,43]. The primary reasons are the increased uptake of ω-6 fatty acids (most typically found in seed crops, which are consequence of modern tendencies in agricultural [30]) and the increased uptake of high energy food with less intake of ω-3 fatty acids.

**Figure 4:**
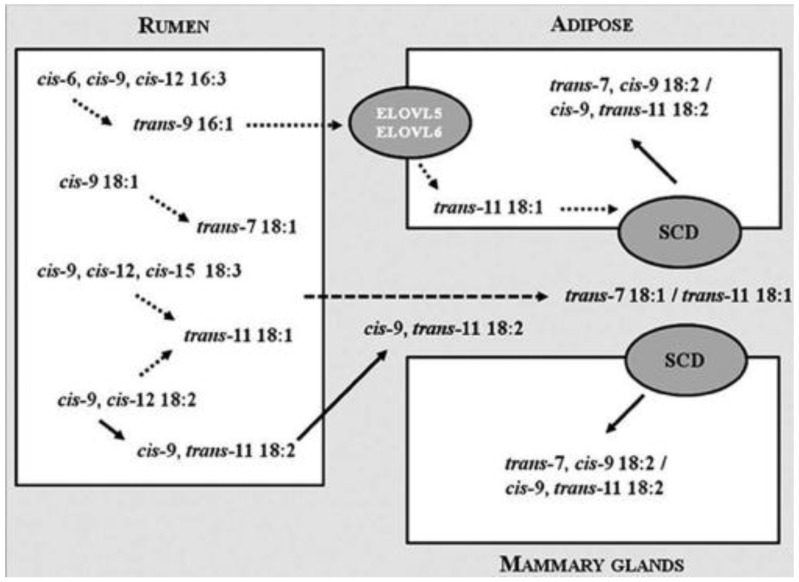
Metabolic pathways of endogenous conjugated linoleic acid synthesis in ruminants. Adopted, with permission, from [45]. Copyright 2014, Royal Society of Chemistry.

Conjugated linoleic acids (CLAs) is an important class of polyunsaturated fatty acids which is a complex mixture of positional (6–8 to 13–15) and geometric (*cis-cis*, *trans-trans*, *cis-trans* and *trans-cis*) fatty acid isomers that contain conjugated double bonds [44]. CLA isomers occur naturally in foods derived from ruminants and are produced as intermediates of the biohydrogenation of poly-unsaturated fatty acids especially linoleic (*cis*-9, *cis*-12) 18:2 and α-linolenic (*cis*-9, *cis*-12, *cis*-15) 18:3 by rumen bacteria [45]. Figure 4 illustrates that most of the *cis*-9, *trans*-11 18:2 CLA incorporated into tissue lipids and milk fat, may be synthesized by the action of stearoyl-CoA desaturase (SCD) on *trans*-11 18:1 in adipose and in the mammary glands. *Trans*-11 18:1 is formed as the intermediate of 18:2 ω-6 and 18:3 ω-3 metabolism in the rumen. *Cis*-9, *trans*-11 18:2 CLA in adipose may also be synthesized from the elongation and desaturation of *trans*-9 16:1 formed in the rumen during the biohydrogenation of 16:3 ω-4 [45].

CLAs have a number of effects on human health with regard to cancer, atherosclerosis, obesity, and immune response [46]. Thus, it has been postulated that the *cis*-9, *trans*-11 18:2 CLA isomer, in a range of animal models, is implicated in anti-carcinogenesis, immunomodulation and anti-atherosclerosis effects. On the contrary, there is accumulating evidence that the *trans*-10, *cis*-12 18:2 CLA isomer may adversely affect insulin sensitivity and blood lipids and can elicit pro-carcinogenic effects in animal models [47,48]. The multiple physiological effects reported for CLA are probably due to contribution to numerous metabolic signaling pathways.

The group of eicosanoids, docosanoids, and other lipid mediators exhibits a large number of isobaric compounds, therefore, detailed MS/MS analysis is required to distinguish and quantify these species. High resolution mass spectrometry (HRMS) allows the separation of many of these isobars by measuring accurate *m*/*z* values of the lipid species and computing the elemental formulae. However, as pointed out recently [15], in several cases isomers were chosen to maximize structural difference and, thus, technical success. The analysis of CLA isomers is also extremely challenging and complex, since it can be very slow and laborious and may require various preparations and analysis steps during which undesirable additional isomerization may take place. This subject has been critically evaluated for various chromatographic and hyphenated techniques [18]. NMR spectroscopy can provide a rapid, reliable and efficient analytical and structural probe with minimum derivatization steps or specific sample preparation procedures, as it will be discussed in detail in the following sections.

## 3. NMR Structural and Analytical Parameters

Naturally occurring lipids consist mainly of the elements hydrogen, carbon, oxygen, nitrogen, and phosphorous. Each of these elements has at least one isotope with a non-zero spin quantum number, I. The ^17^O nucleus (I = 5/2) has very low natural abundance (0.037%), very low gyromagnetic ratio (−3.628 × 10^7^ rad T^−1^ s^−1^), extremely low receptivity with respect to that of ^1^H (~1.08 × 10^−5^) [49] and very broad NMR resonances due to quadrupolar relaxation [50,51,52]. These unfavorable nuclear properties do not allow the routine NMR use of this nucleus. The ^14^N nucleus (I = 1) has a high natural abundance (99.63%), very low gyromagnetic ratio (~1.938 × 10^7^ rad T^−1^ s^−1^) and small receptivity (~1 × 10^−3^) with respect to that of ^1^H. Furthermore, relatively large quadrupolar coupling constants and, thus, broad resonances limit applications of this isotope to nitrogen sites with spherical electronic symmetry around the nitrogen nucleus. The ^15^N isotope has a spin quantum number I = 1/2, very low natural abundance of 0.37%, very low gyromagnetic ratio (−2.71 × 10^7^ rad T^−1^ s^−1^) and extremely low receptivity (0.39 × 10^−5^) with respect to that of ^1^H and, thus, has been found very limited applications [53]. Therefore, for the purpose of the present review article only ^1^H, ^13^C and ^31^P nuclei will be discussed.

### 3.1. ^1^H-NMR Spectroscopy

The ^1^H nucleus with spin quantum number I = 1/2, high gyromagnetic ratio (26.75 × 10^7^ rad T^−1^ s^−1^) and natural abundance of 99.985% is the most sensitive NMR probe and, thus, appropriate for investigating even minor unsaturated lipids within a short experimental time. ^1^H nuclei can lead to different NMR signals when hydrogens are in different chemical environments. Peaks in a specific spectral region can, therefore, be assigned to certain structural elements and the amount of different groups in lipids can be determined.

**Figure 5:**
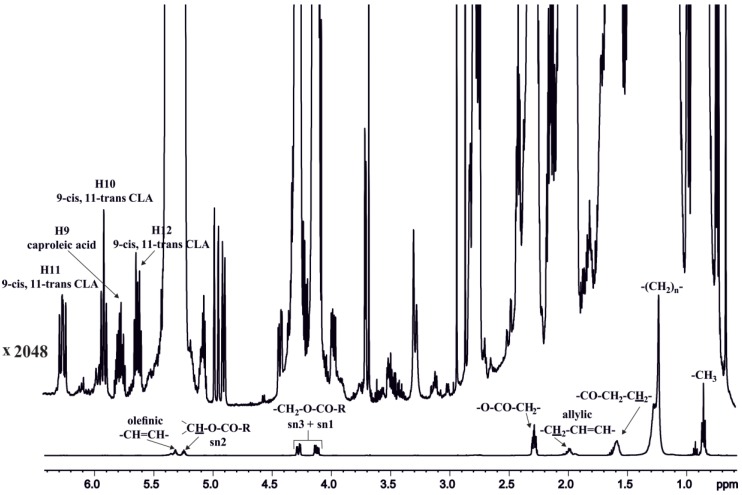
500 MHz ^1^H-NMR spectrum of the lipid extract of a milk sample in CDCl_3_ (298 K, acquisition time (AQ) = 4.0 s, relaxation delay (RD) = 3.6 s, total experimental = 1 h 5 min). The inset shows 2048× magnification of the spectrum in order to display some resonances of the minor species 9-*cis*, 11-*trans*, 18:2 CLA and caproleic acid.

A typical ^1^H-NMR spectrum of the lipid fraction of a milk sample is illustrated in Figure 5. The ^1^H-NMR chemical shifts can be grouped in several broadly defined regions: the olefinic protons of isolated double bonds in the region of 6.2–5.30 ppm, the olefinic protons of conjugated double bonds in the region of 6.40–5.20 ppm, the protons of the glycerol moiety of 5.10–3.70 ppm, the allylic protons of 3.05–2.60 ppm, the α-CH_2_ protons of 2.50–2.30 ppm, the CH_2_–CH=CH protons of ~2.0 ppm and the (CH_2_)_n_ and CH_3_ protons in the regions of 1.60–1.2 ppm and 0.98–0.86 ppm, respectively. Resonances can also be observed in the region of 10.5–8 ppm which can be attributed to the autoxidation of unsaturated fatty acids. This process results in the formation of hydroperoxides and, subsequently, in the chain scission and the formation of shorter chain saturated and unsaturated aldehydes. This topic will be analyzed in Section 5.4.

#### 3.1.1. Olefinic Protons

The olefinic protons, –CH=CH–, of unsaturated fatty acids appear mainly in the region of 5.2–5.5 ppm. Exception is the terminal ω-1 double bond (vinyl group) of caproleic acid which results in a multiplet at 5.79 ppm (H9) and two doublets of quadruplets at 4.91 ppm and at 4.97 ppm due to H10 and H11 protons (Figure 5). Similarly, the olefinic ^1^H-NMR chemical shifts of 2-*cis* methyl octadecenoate are deshielded due to conjugation of the COOH group and depend upon the configuration of the double bond (Table 3) [54].The ^1^H-NMR chemical shifts of the various positional isomers were explained in terms of long-range deshielding effects which depend upon the number of intervening CH_2_ groups from the carboxyl group. However, it is clear from Table 3 that reliable distinction cannot be made with respect to the position and configuration of the double bonds at positions above 4-*cis*/4-*trans*, especially in mixture analysis.

**Figure 6:**
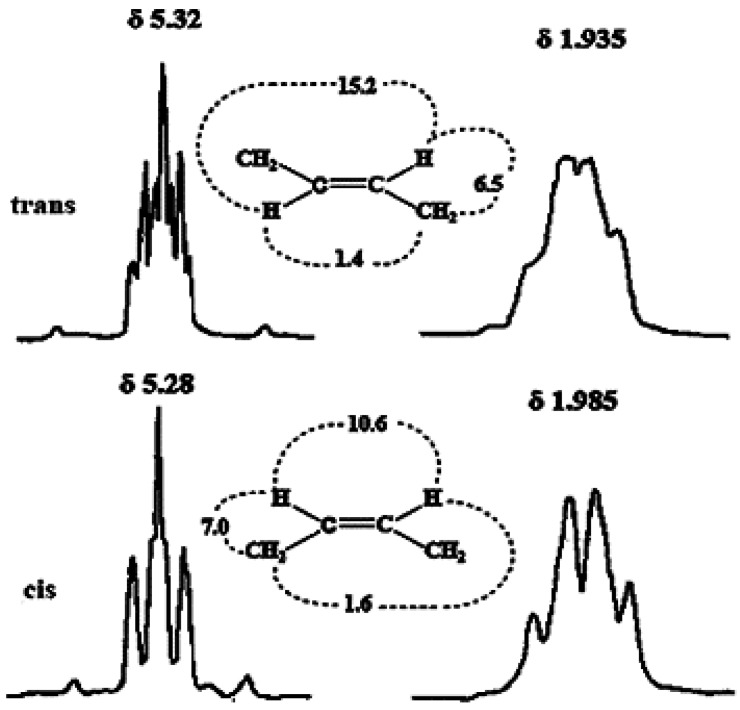
Computer simulated ^1^H-NMR signals for alkenyl and allylic protons in isolated *cis* and *trans* double bonds. Adopted, with permission, from [54]. Copyright 1975, North-Holland Publishing Company.

The geometric isomerization of the double bond could, in principle, be determined by the coupling constant of the methylene protons which is larger for the *trans* (^3^*J* ≈ 15 Hz) than that of the *cis* bonds (^3^*J* ≈ 10 Hz). However, in practice, this is very difficult because the chemical shift differences of the methylene proton are very small and, thus, the spin coupling system illustrates a second order spin pattern. Figure 6 shows computer simulations of alkenyl protons of isolated *cis* and *trans* double bonds under the hypothesis that there is no chemical shift difference between the two alkenyl protons. Determining the *cis*/*trans* stereochemistry of lipid double bonds with the use of ^1^H-NMR of the olefinic protons, therefore, is problematic in lipid analysis and, at this stage, such data are mostly limited to the identification of stereoisomers from a limited number of standards. Recent progress has been achieved with the use of band selective ^1^H-^13^C HMBC experiments (Section 4.7) and shift reagents (Section 4.9).

#### 3.1.2. Olefinic Protons in Conjugated Double Bonds

Conjugated linoleic acids (CLAs) have been extensively investigated using ^1^H-NMR (Table 4) including the (*cis*-9, *trans*-12) 18:2 isomer, which is the dominant species in dairy products, and several other positional and geometric isomers [55,56] which may have different biological functions. Figure 5 illustrates that the ^1^H-NMR spectrum of CLAs in the lipid fraction of milk exhibit highly diagnostic resonances due to the –CH= protons of the conjugated double bonds in the region of 6.35 to 5.50 ppm. Some of these resonances are clearly shifted to higher frequencies with respect to protons of isolated double bonds, thus, illustrating the increased delocalization of conjugated double bonds. The “inner” olefinic protons of C10 and C11 are deshielded with respect the olefinic protons of C9 and C12. Furthermore, in the mixed *cis/trans* isomers the signals of the *cis* bonds are deshielded with respect to those of the *trans* bonds. In the case of identical bond configuration, the signals of the two “inner” protons overlap as well as the two signals of the “outer” protons.

Conjugated linolenic acids (CL_n_As) is a group of positional (8, 10, 12–18:3; 9, 11, 13–18:3; 10, 12, 14–18:3; etc.) and geometric (*trans-trans-trans*, *cis-trans-trans*, *cis-trans-cis* etc.) [57,58] isomers which occur in significant amounts in some seed oils such as tung oil, catalpa and cherry seed oil. NMR studies of CL_n_As are limited to ^13^C-NMR [57] and ^1^H and ^13^C-NMR [58] data of α-eleostearic acid: (9-*cis*, 11-*trans*, 13-*trans*) 18:3, β-eleostearic acid: (9-*trans*, 11-*trans*, 13-*trans*) 18:3 and punicic acid: (9-*cis*, 11-*trans*, 13-*cis*) 18:3 (Table 5). Due to extensive overlap of the ^1^H-NMR signals, homonuclear ^1^H decoupling techniques were extensively used for the simplification of the ^1^H-NMR spectra and calculation of the ^3^*J* coupling constants. In the all-*trans* β-eleostearic acid, three multiplets at 6.10(2H) ppm, 6.04(2H) ppm, and 5.66(2H) ppm were found due to near magnetic equivalence of the protons of each group. As in the case of CLAs, conjugation results in a change in the charge density of the “inner” protons and, thus, shielding compared with the olefinic protons in the “outer” position.

#### 3.1.3. Protons of the Glycerol Moiety 

The location of acyl chain esterification on the glycerol backbone was assigned as *sn*-1, *sn*-2 and *sn*-3 (Figure 2). NMR spectroscopy can be used to provide information on the regiospecific distribution of fatty acids of triacylglycerols (TAGs) and phospholipids. 1,3-triglycerides (TAG), 1- and 2-monoacylglycerides (MAG) and 1,2-diacylglycerides (DAG) can be identified on the basis of the chemical shifts of the protons attached to glycerol carbons (Table 6). The proton signal at 5.26 ppm (m, *J*_1a′,2′_ = 5.88 Hz) was assigned to H2 of the glycerol backbone of TAG. The H1 and H3 protons of the glycerol moiety in TAG appear at 4.15 and 4.30 ppm. The 2′-CH–OCO– and HO–CH_2_–CH resonances of *sn*-1/2,3 DAG, the 1′b-CH_2_–OCO– and 1′a-CH_2_–OCO– resonances of *sn*-1,2 DAG and the 1′b, 3′b-CH_2_–OCO–, 1′a, 3′a-CH_2_–OCO– and –CH_2_–OCO resonances of *sn*-1,3 DAG have also been assigned in oils. The 3′a-CH_2_–OCO– resonance of 1-MAG appears at 3.59 ppm.

#### 3.1.4 Bis-allylic Protons 

The bis-allylic protons, =HC–CH_2_–CH=, in polyenoic fatty acids and esters are in a characteristic region of 2.60–2.85 ppm due to the effect of the magnetic anisotropy of the double bonds. The chemical shifts appear to be related to the number and the geometric configuration of the double bonds (Table 7). Thus, the bis-allylic protons of DHA, α-linolenic acid and linoleic acid appear at 2.85, 2.82 and 2.78 ppm, respectively. The bis-allylic protons of α-linolenic acid are more deshielded (δ = 2.82 ppm) than in linoleic acid (δ = 2.78 ppm) due to a larger number of double bonds in the former case. The *cis-cis* configuration results in a stronger deshielding effect with respect to *cis-trans* and *trans-trans* configuration.

The deshielding effect in polyyonic fatty acids and esters is stronger than that observed in polyenoic fatty acids and esters (Table 8). This may be attributed to the position of the allylic protons in the deshielding region of the magnetic anisotropy of the triple bond.

#### 3.1.5. α-CH_2_, Allylic CH_2_–CH=CH and (CH_2_)_n_ Protons

The CH_2_–CH_2_–COOH protons of DHA result in a composite signal at 2.46 ppm due to the combined deshielding effect of the COOH group and the ω-3 double bond (Table 9). This chemical shift is highly diagnostic for the presence of DHA in mixture analysis especially in fish oils (Section 5.1). The –OOC–CH_2_–CH_2_– protons of all fatty acids appear at 2.33 ppm. The double bond configuration influences the chemical shift of the –CH_2_–CH=CH– protons of UFA. In particular, the methylene protons adjacent to a *cis* double bond is deshielded by ~0.05 ppm with respect to that adjacent to a *trans* bond.

#### 3.1.6. The CH_3_ Protons 

The –CH_3_ group of α-linolenic acid appears at 0.98 ppm (triplet, ^3^*J* = 7.5 Hz), the –CH_3_ group of butyric acid at 0.94 ppm (^3^*J* = 7.4 Hz), and the –CH_3_ terminal groups of the rest of the fatty acids in a narrow range of chemical shifts of 0.885 to 0.87 ppm (triplets, ^3^*J* = 6.9 to 7.3 Hz). α-linolenic acid (18:3, ω-3) contains a double bond close to the terminal –CH_3_ group that causes a shift to higher ppm values from 0.89 to 0.98 ppm.

**Figure 7:**
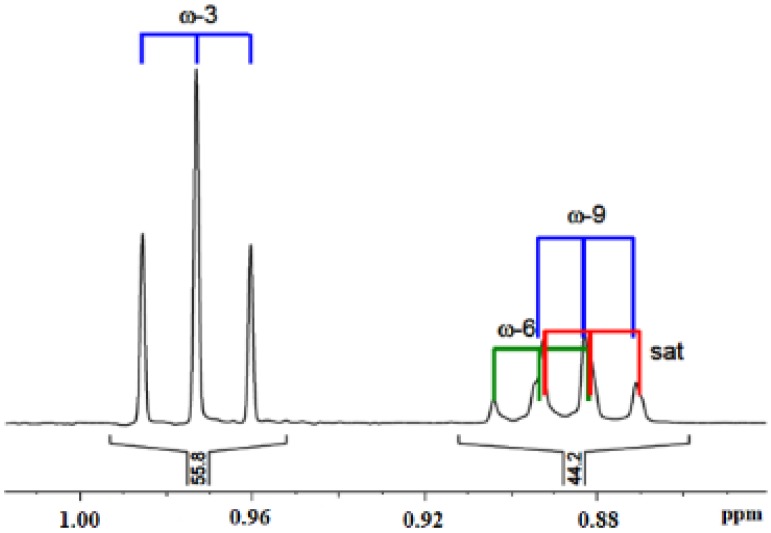
Separation of ω-3 from ω-6, ω-9 and saturated fatty acids on the basis of ^1^H-NMR chemical shifts of –CH_3_ groups. Free download from: http://www.ilps.org/index.php/Standardized_Methods.html.

Figure 7 illustrates that ω-3 fatty acids can be clearly identified from the other lipids on the basis of the chemical shifts of the terminal methyl group. Interestingly, the apparently quartet at ~0.88 ppm is due to superposition of three triplets which results in the identification of the ω-6 and ω-9 fatty acids from the saturated FA. This demonstrates the significant long range effect of the double bond on the chemical shifts of the terminal CH_3_ groups. It should be emphasized, however, that sufficient separation of the triplets would require either the use of ultra-high magnetic fields or advanced 2D ^1^H-^13^C HSQC-TOCSY (Section 4.6) and band selective ^1^H-^13^C HMBC (Section 4.7) experiments.

#### 3.1.7. ^1^H-NMR Relaxation Times 

In order to obtain quantitative NMR data and avoid differential saturation effects, the protons should be fully relaxed. This demands recycling time i.e., acquisition time + relaxation delay of at least 5 × T_1_, where T_1_ is the longitudinal relaxation time of the slowest relaxing nuclei. The longitudinal relaxation times (T_1_) of the ^1^H nuclei of the acyl chains of triglycerides in oils were found to be sufficiently short, the longest being that of the terminal CH_3_ group (1.51 s) (Table 10), and substantially shorter than those of the ^13^C nuclei (see Section 3.2). This implies that very good S/N ratio and, thus, quantitative results can be obtained even for minor analytes within relatively short period of time.

### 3.2. ^13^C-NMR Spectroscopy 

The ^13^C nucleus with I = 1/2 has become a very useful analytical and structural tool in lipid research despite its low gyromagnetic ratio (6.726 × 10^7^ rad T^−1^ s^−1^), its low natural abundance (1.108%) and the low receptivity with respect to that of ^1^H (1.7 × 10^−4^). This may be attributed to the fact that ^13^C has a large chemical shift range (~200 ppm) with respect to that of ^1^H (~10 ppm), the significant advances in NMR instrumentation, the use of versatile pulse sequences, polarization transfer experiments and sophisticated 2D ^1^H-^13^C NMR experiments. High resolution ^13^C-NMR, therefore, has emerged as a versatile technique for the analysis of lipids, since the pioneering work of Shoolery [61], Gunstone [19,62] and Wollenberg [63], which allows the characterization of the isomeric distribution of omega-3 PUFA in lipid fractions. ^13^C-NMR was demonstrated to be an excellent method in determining the distribution of triacylglycerols and the position of the fatty acids on the glycerol backbone and in obtaining quantitative information about the fatty acid composition of vegetable oils, vegetable seeds and other lipid extracts, as will be analyzed below.

The ^13^C-NMR chemical shifts of lipids can be grouped in four regions: the carbonyl and carboxyl carbons in the region of 172–178 ppm, unsaturated carbons of 124–134 ppm, the glycerol backbone carbons of 60–72 ppm and aliphatic carbons of 10–35 ppm.

#### 3.2.1. Carbonyl and Carboxyl Carbon Region 

**Figure 8:**
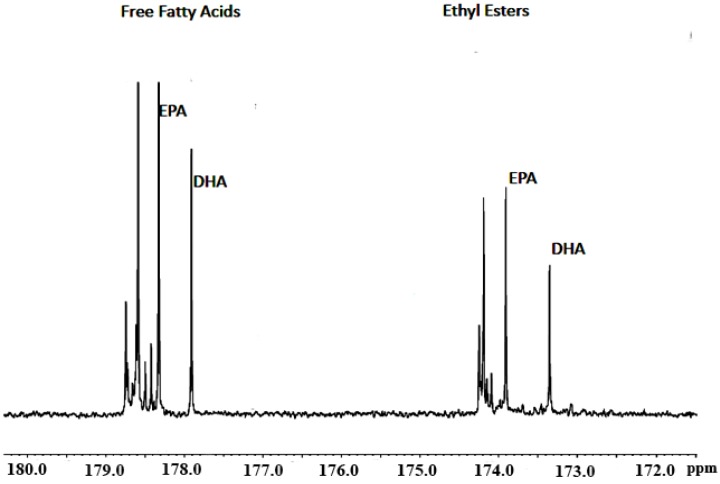
^13^C-NMR simultaneous detection of free fatty acids (left side) and their alkyl esters (right side). Free download from: http://www.ilps.org/index.php/Standardized_Methods.html.

The carboxyl carbon atoms of the free fatty acids appear in a narrow but distinct region of 177.5 to 180 ppm. The resonances are shifted to high frequency with respect to ethyl esterified derivatives by ~4 ppm (Table 11 and Figure 8). This high frequency shift is sufficient for the quantification of free fatty acids with respect to esterified lipids. For accurate quantification results, however, caution should be exercised since the relaxation times of carboxyl and carbonyl carbon atoms (Table 12) are significantly longer than those of ^1^H-NMR (Table 10). To obtain quantitative data, the relaxation problem can be resolved by adding a relaxation reagent, such as chromium acetonylacetonoate (Cr(acac)_3_), or by recording proton decoupled NOE suppressed ^13^C-NMR spectra with sufficiently long interpulse delay (~5 T_1_, where T_1_ is the longest relaxation time of a lipid mixture).

The carbonyl carbons of fatty acids of TAG appear as two sets of resonances: the high frequency group which includes the chains esterified at 1(3)-glycerol positions and the low frequency group which includes the 2-glycerol position chains. The chemical shift difference of about 0.42 ppm was explained by the effect of two γ-gauche interactions on the C=O group of 2-position with respect to one interaction of the carbonyls of 1(3) chains [64].

**Figure 9:**
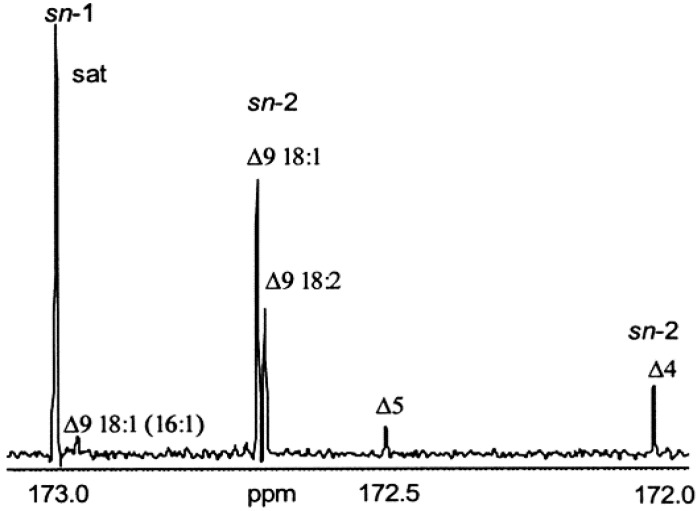
^13^C-NMR of the carbonyl region of docosahexaenoic acid-enriched egg yolk phosphatidylcholine. Adopted, with permission, from [21]. Copyright 2001, Wiley-VCH.

The carbonyl carbons of long chain saturated and unsaturated acyl groups in *sn*-1,3 TAG appear at 172.8–172.7 ppm while those in *sn*-2 TAG at 172.41–172.38 ppm. Saturated and unsaturated acyl groups in *sn*-1,3 DAG appear at 173.35–173.32 ppm. Acyl chains in the external position in *sn*-1,2/-2,3 DAG appear at 173.18 ppm while those in *sn*-2 at 173.08 ppm. It should be emphasized that the ^13^C-NMR carbonyl and carboxyl signals enable the selective identification of different Δ type fatty acids i.e., DHA which is a Δ^4^ and EPA which is a Δ^5^ fatty acid (Figure 8). The carbonyl signals can also be used for the integration of the position of unsaturated fatty acids within the glycerol moiety (*sn*-1,3 and *sn*-2). Thus, ^13^C-NMR enables the identification and, thus, quantification of *sn*-1 saturated with respect to *sn*-1 Δ^9^ 18:1 and the *sn*-2 distribution of Δ^9^ 18:1, Δ^9^ 18:2, Δ^5^ and Δ^4^ without the need for the isolation of the individual lipid components (Figure 9).

#### 3.2.2. Olefinic Carbon Region 

The ^13^C-NMR chemical shift range of the olefinic carbons (124–134 ppm) is wider than that of the carbonyl and carboxyl carbons (Table 13 and Table 14). Nevertheless, the olefinic carbon region is usually the most complex part of the spectrum in lipid mixture analysis. The unsaturated carbons were resolved according to the position of the double bond with respect to the C=O carbons and the double bond number. The non-equivalence of the ^13^C chemical shifts of olefinic carbons has been investigated in a number of mono-unsaturated fatty acids with different number of C–C bonds separating the double bond and the ester group. The unsaturated carbon nearest to the chain of the ester group is shifted to lower frequency from the unsaturated carbon nearest to the chain methyl end. This allows the shift differences of the double bond pair to be used as a criterion of the number of C–C bonds intervening between the C=O and the C=C bonds [64,65,66]. The carbon of the double bond closer to the carbonyl C=O bond in the *sn*-2 position is shielded with respect to that in the 1(3)-position. The opposite trend was observed for the carbon which is further from the C=O bond. The chemical shift differences were explained in terms of a linear electric field effect of the C=O group [67].

Davis et al. [68] suggested the use of shift increments to predict the configuration of an unknown CLA geometric and positional isomer. For a number of (7-*cis*, 9-*trans*), (8-*cis*, 10-*trans*), (9-*cis*, 11-*trans*), (10-*cis*, 12-*trans*) and (11-*cis*, 13-*trans*) 18:2 positional isomers, the differential chemical shift, Δδ, between C_1ol_ (the olefinic carbon closest to the carboxyl group) and C_4ol_ (the olefinic carbon most remote from the carboxyl group) shows a gradual and significant shift as the conjugated system becomes more remote from the carboxyl function (Table 15).

#### 3.2.3. Glycerol Carbons

The ^13^C-NMR signals of the glycerol carbon occur in the region of 60–72 ppm (Table 16). 2-monoacylglycerols, 1,3-diacyglycerols and triacylglycerols result in two signals for the glycerol moiety with intensity rations 1:2. The asymmetrical 1-monoacyglycerols and 1,2-diacyglycerols result in three signals. The resonances of the glycerol C-2 of all glycerides are shifted to higher frequencies with respect to those of the glycerol 1(3)-carbons. The length and degree of unsaturation of the acyl chains has been shown to influence the chemical shifts of the glycerol carbons (see Section 5.6.3).

#### 3.2.4. Aliphatic Carbons

The ^13^C-NMR chemical shift range of the aliphatic carbons (Table 17) is very wide and has been utilized extensively for the identification and quantification of lipids. The mixed geometric (9-*cis*, 11-*trans*) 18:2 and (9-*trans*, 11-*cis*) 18:2 CLA isomers were identified on the basis of the carbon chemical shifts of the allylic methylene groups. Resonances at δ(^13^C) ≈ 27.6 ppm are characteristic of an adjacent *cis*-double bond and signals at δ(^13^C) ≈ 32.8 ppm to methylene carbons adjacent to the *trans*-bond.

**Figure 10:**
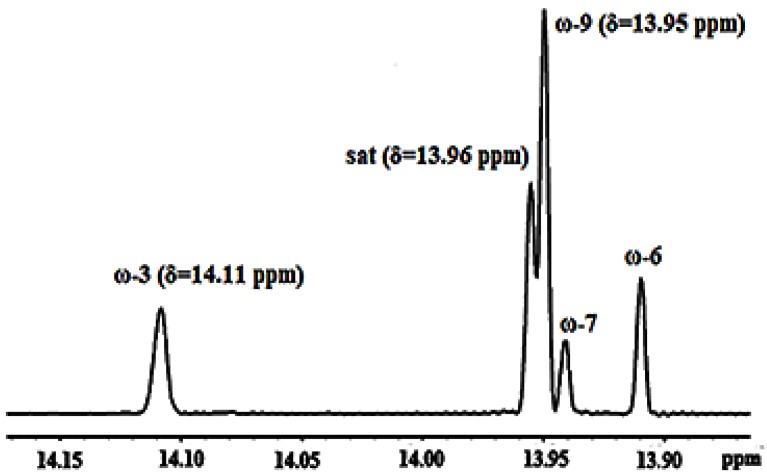
^13^C-NMR chemical shifts of the terminal methyl groups allow the identification and quantification of ω-3, ω-6, ω-7, ω-9 and saturated fatty acids of a salmon oil. Free download from: http://www.ilps.org/index.php/Standardized_Methods.html.

**Figure 11:**
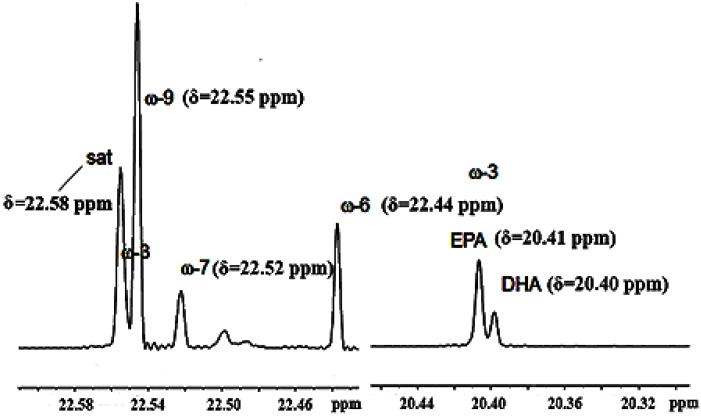
^13^C-NMR chemical shifts of the methylene C-2 carbons allow the identification of the EPA and DHA ω-3 fatty acids. Free download from: http://www.ilps.org/index.php/Standardized_Methods.html.

The length of saturated FA and acyl chains and the proximity of the last double bond to the methyl end, strongly affects the ^13^C chemical shift which can be utilized for the classification and quantification of ω-3, ω-6 and ω-7 fatty acids (Figure 10). The ^13^C resonance of the CH_3_ groups of ω-3 fatty acids is deshielded by 0.15 ppm with respect to the CH_3_ group of saturated fatty acids. Separation of the ω-6, ω-7 and ω-9 UFA can also be achieved, however, the chemical shift do not follow a regular pattern with respect to the number of intervening CH_2_ bonds (Figure 10). The methylene C-2 carbons demonstrate a clear separation of the ω-3 EPA (δ = 20.41 ppm) and DHA (δ = 20.40 ppm) (Figure 11).

Combination, therefore, of the –CH_3_ and methylene C-2 spectral regions results in chromatographic like NMR methodology for the identification and quantification of various PUFA, even when using a medium strength spectrometer (500 MHz for ^1^H).

### 3.3. ^31^P-NMR Spectroscopy

The ^31^P nucleus has I = 1/2, high gyromagnetic ratio (10.829 × 10^9^ rad T^−1^ s^−1^) and a wide range of chemical shifts, 100% natural abundance and, thus, high sensitivity, which is only 15 times less than that of the ^1^H nucleus [23]. ^31^P-NMR has been shown to be a powerful method in phospholipid analysis. ^31^P-NMR spectra show separate signals depending on the fatty acid distribution of the phospholipids. In soybean lecithin ~20% of the fatty acids are saturated and nearly 100% of them are distributed in the *sn*-1 position. The unsaturated fatty acids are distributed in the *sn*-1 and *sn*-2 positions. ^31^P-NMR was able to distinguish between double unsaturated and mixed saturated/unsaturated phospholipids. Similarly, the ^31^P of phosphatidic acid (PA) shows two unequal signals due to the presence of the mixed saturated / unsaturated (40%) and unsaturated/unsaturated (60%) lipids [21,69].

**Scheme 1:**
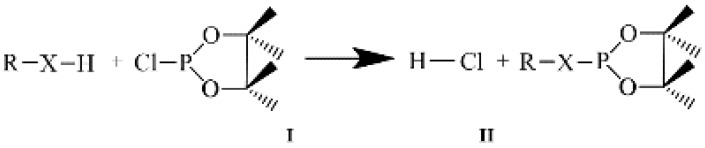
Reaction of hydroxyl and carboxyl groups with the phosphorus reagent 2-chloro-4,4,5,5-tetramethyldioxaphospholane (**I**) and the phosphitylated product (**II**).

^31^P-NMR has been applied in the analysis of olive oil and other vegetable oils [59,70,71,72] and, especially, in cases where strong signal overlap and dynamic range problems in ^1^H-NMR spectra and/or long relaxation times of the insensitive ^13^C nuclei make the analysis of oils a difficult task. Application of ^31^P-NMR in the analysis of olive oil and other vegetable oils was achieved by derivatization of the labile hydrogens of functional groups, such as hydroxyl and carboxyl groups of the oil constituents with the phosphorus reagent 2-chloro-4,4,5,5-tetramethyldioxaphospholane (**I**) and the use of ^31^P chemical shifts to identify labile groups (compound **II**). The phosphitylation reaction (Scheme 1) is quantitative, takes place under mild conditions (within the NMR tube) and is completed in less than 15 min. Using this reaction, it was possible to quantify DAG and MAG species and free glycerol in oils. Quantitative ^31^P requires the use of the inverse gated decoupling technique in combination with the paramagnetic chemical shift reagent Cr(acac)_3_ and a relaxation delay 5T_1_ ≈ 4.6 s, where T_1_ is the relaxation time of the internal standard. The disadvantage of the ^31^P-NMR methodology is the destruction of the analytes.

## 4. NMR Methods for Assignment

### 4.1. Selective Suppression of Major Signals

^1^H-NMR spectroscopy has found limited application in the identification and quantification of minor species in lipid extracts. This is due to the fact that the equilibrium magnetization of e.g., the olefinic (CH_2_)_n_^1^H spins of the major constituents of the lipids is 10^2^–10^4^ greater than the equilibrium magnetization of the ^1^H spins of the minor species. The problem is particularly severe in two dimensional experiments in which changes in the phase of the strong signals during various increments can lead to severe t_1_ noise. A straightforward method of reducing major resonances is the saturation of selective resonances by the use of variable frequency presaturation.

**Figure 12:**
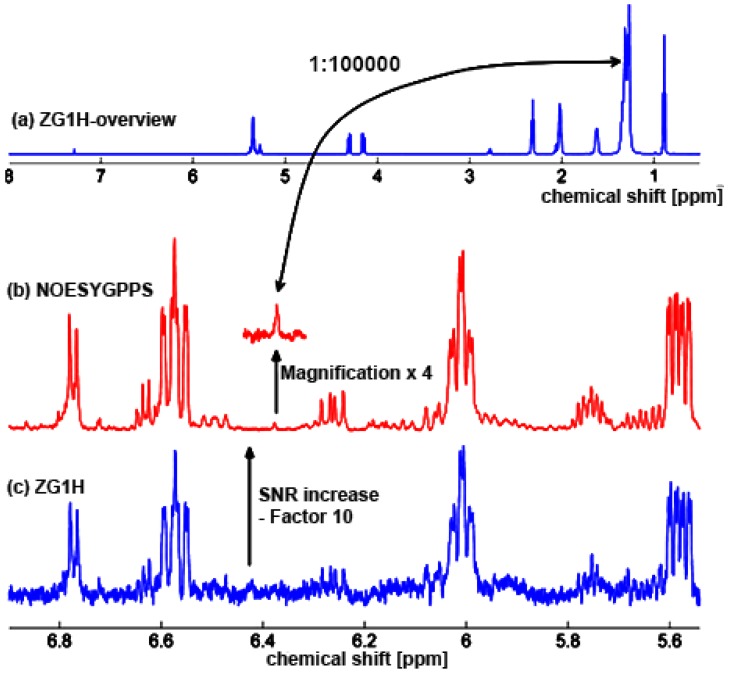
Signal-to-noise (SNR) increase by a factor of ~10 was obtained after suppression of multiple dominating lipid signals due to the increase of the receiver gain. Number of scans and total experimental time were kept constant, i.e., NS = 32 and 4 min, respectively. Adopted, with permission, from [73]. Copyright 2011, Elsevier Ltd.

Figure 12 illustrates that a S/N ratio increase by a factor of ~10 was achieved after multiple suppression of dominating lipid signals due to increase in the receiver gain of the spectrometer [73]. Similarly, selective suppression of the two major lipid resonances at 0.90 and 1.20 ppm in the lipid fraction of a lyophilized milk resulted in an increase in the S/N ratio of the minor CLA resonances by a factor of ~3 which allowed the identification and quantification of minor CLA isomers [56].

### 4.2. Selective 1D TOCSY Experiments

**Figure 13:**
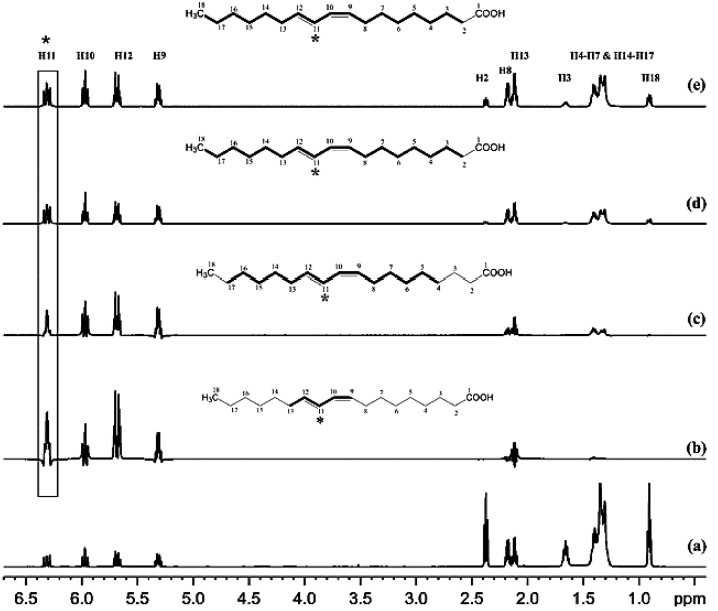
(**a**) 500 MHz 1D ^1^H-NMR spectrum of 20 mM solution of the (9-*cis*,11-*trans*) 18:2 CLA in CDCl_3_ (T, 298 K; acquisition time, 4.3 s; relaxation delay, 5 s; number of scans, 256; and experimental time, ~25 min). (**b**–**e**) Selective 1D TOCSY spectra of the above solution using a mixing time of τ_m_ = 33 ms (**b**), 70 ms (**c**), 200 ms (**d**), and 400 ms (**e**). The asterisk denotes the selected H11 resonance that was excited. For panels (**b**–**e**), the magnetization transfer network is illustrated. Adopted, with permission, from [78]. Copyright 2015, American Chemical Society.

The selective 1D TOCSY experiment is an important NMR technique for establishing ^1^H-^1^H connectivity via scalar coupling in small and medium size molecules [74,75]. However, the method has found limited applications in complex mixtures and, more specific, in natural products and food extracts [76,77,78,79]. Figure 13 illustrates a series of selective 1D TOCSY spectra of the (9-*cis*, 11-*trans*) 18:2 CLA isomer. The H11 olefinic proton (δ = 6.27 ppm) was selected for excitation using mixing times τ_m_ of 33 ms, 70 ms, 200 ms and 400 ms. For mixing times of τ_m_ = 200 ms and 400 ms the complete assignment and structure of the compound can be achieved with very good resolution. In mixture analysis what is required, is an isolated target peak to be used as the magnetization transfer source. This is the case e.g., of CLA olefinic resonances which provided a means to distinguish a lipid in complex lipid fractions not only at a class level, with reference to molecular fragments (saturated, monounsaturated, bis-allylic, and polyunsaturated), but also at the level of individual compounds (Section 5.2).

### 4.3. Homonuclear 2D ^1^H-^1^H COSY and 2D ^1^H-^1^H TOCSY Experiments [80,81,82]

**Figure 14:**
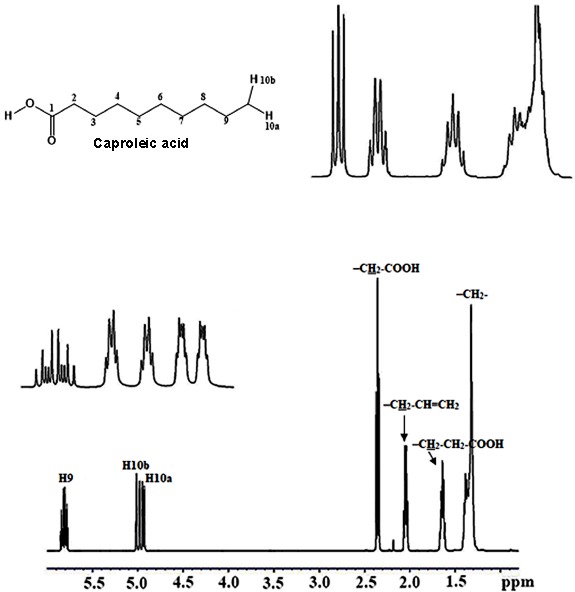
500 MHz 1D ^1^H-NMR spectrum of caproleic acid in CDCl_3_ (concentration: 5 mM, T: 298 K, acquisition time: 2.7 s, relaxation delay: 8 s, number of scans: 128, experimental time: ~23 min).

**Figure 15:**
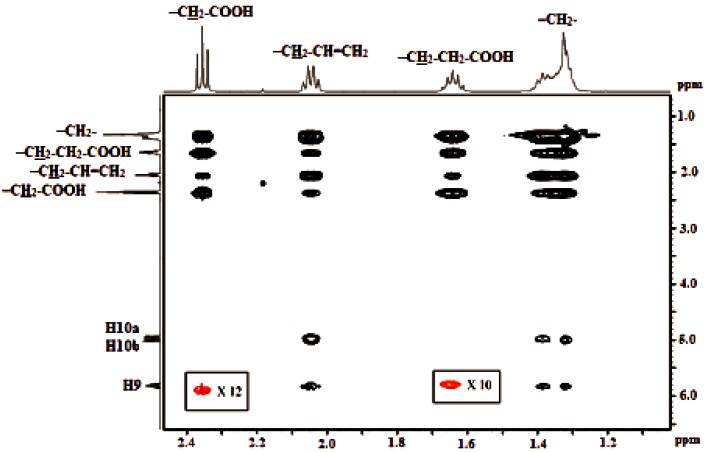
Selected region of 500 MHz 2D ^1^H-^1^H TOCSY spectrum of caproleic acid in CDCl_3_ (solution conditions the same as in Figure 14). Mixing time τ_m_ = 100 ms, number of scans (ns) = 32, number of increments = 256, total experimental time = 5 h 19 min.

The ^1^H-NMR spectrum of the caproleic acid (Figure 14) shows the resonances due to the protons of the –CH=CH_2_ proton at 5.79 ppm (multiplet), the =CH_2_ protons at 4.98 ppm (H10b, dq) and 4.92 ppm (H10a, dq), the –CH_2_–COOH protons at 2.35 ppm (triplet), the –CH_2_–CH=CH_2_ protons at 2.05 ppm (multiplet), the –CH_2_-CH_2_–COOH protons at 1.64 ppm (multiplet) and all –CH_2_– protons at 1.25 ppm (multiplet). Figure 15 illustrates a selected region of the 2D ^1^H-^1^H TOCSY spectrum of the caproleic acid. The use of mixing time τ_m_ = 100 ms cause magnetization transfer throughout the whole number of protons of caproleic acid. The H9 (5.79 ppm) correlates with the H8 (2.05 ppm) and the H7 (1.35 ppm). The red peaks show the weak correlations between H9 (5.79 ppm)–H2 (2.35 ppm) and H9 (5.79 ppm)–H3 (1.65 ppm). This also provides unequivocal assignment of the H9 with respect to H10a,b and H2 for the given mixing time.

**Figure 16:**
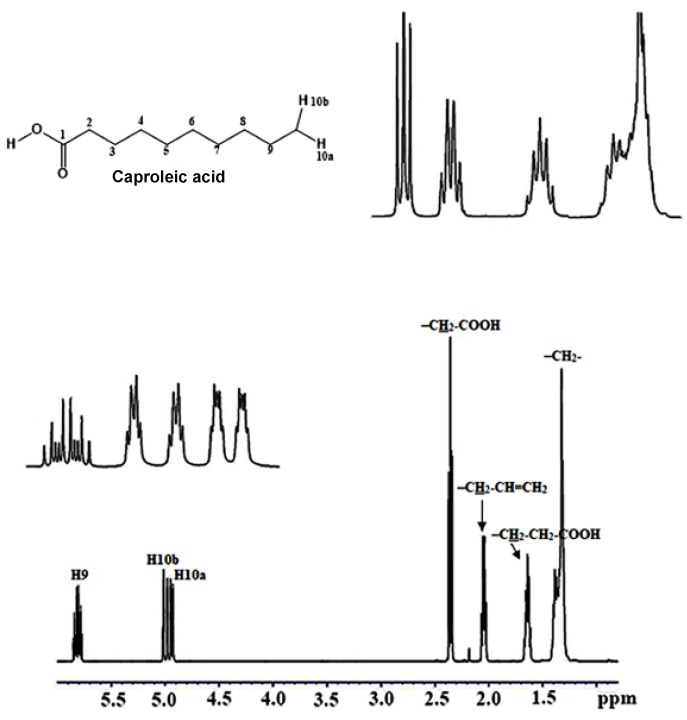
500 MHz 1D ^1^H-NMR spectrum of (9-*cis*, 11-*trans*) 18:2 CLA in CDCl_3_ (concentration: 10 mM, T: 298 K, acquisition time: 2.7 s, relaxation delay: 8 s, number of scans: 128, experimental time: ~23 min).

**Figure 17:**
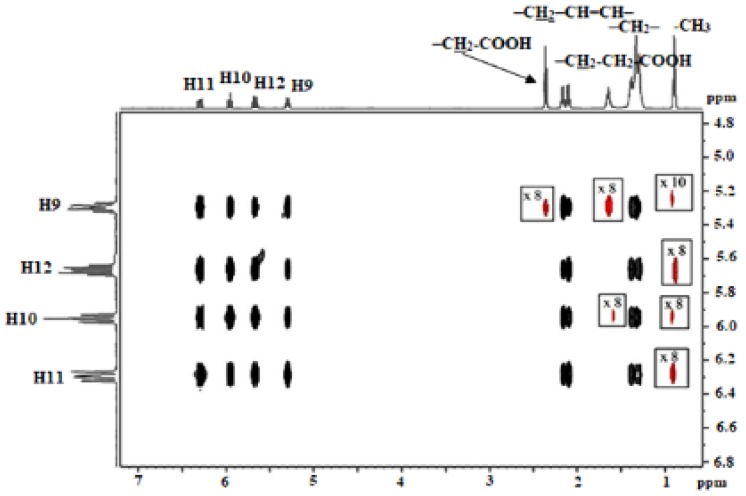
Selected region of 500 MHz 2D ^1^H-^1^H TOCSY NMR spectrum of (9-*cis*, 11-*trans*) 18:2 CLA (solution conditions the same as in Figure 16). Mixing time τ_m_ = 100 ms, number of scans (ns) = 16, number of increments = 256, total experimental time = 2 h 39 min.

The 1D ^1^H-NMR and 2D ^1^H-^1^H NMR spectra of the (9-*cis*, 11-*trans*) 18:2 CLA isomer are illustrated in Figure 16 and Figure 17, respectively. Again the use of mixing time τ_m_ = 100 ms cause the polarization to spread out the whole number of protons. Thus, the olefinic H9 proton demonstrates cross peaks, at lower contour level, with both H2 and H18 protons (Table 18). The assignment, however, of the composite signals at 1.25–1.35 ppm due to H4-H7 and H14-H17 protons would require the use of several 2D ^1^H-^1^H TOCSY or selective 1D ^1^H TOCSY with various mixing times. The 1D ^1^H TOCSY experiment has the following advantages: (i) significantly increases the digital resolution while in the 2D TOCSY it is limited by the number of points taken in the indirect dimension, (ii) removes dynamic range issues and detects cross peaks even in regions with strong signal overlap of lipid extracts and (iii) decreases significantly the experimental time [78].

### 4.4. ^1^H-^13^C Heteronuclear Single-Quantum Correlation Spectroscopy (^1^H-^13^C HSQC)-Band Selective ^1^H-^13^C HSQC [80,81,82,83]

^1^H-^13^C HSQC spectroscopy detects correlations between ^1^H-^13^C nuclei which are separated by one bond. ^1^*J* (^1^H-^13^C) couplings are typically optimized for 145 Hz, being an average value for one bond couplings [83,84,85]. This method results in one cross peak per pair of coupled nuclei whose two coordinates are the chemical shifts of ^1^H (observed nucleus) and ^13^C (indirectly detected nucleus). This experiment enables: (i) proton assignments to be mapped directly into their bonded carbons and, thus, may be used to spread complex ^1^H-NMR spectra, as in the case of lipids, according to the greater dispersion of the carbon resonances and (ii) quantification of minor lipids even in complex mixtures (see Section 5.2).

**Figure 18:**
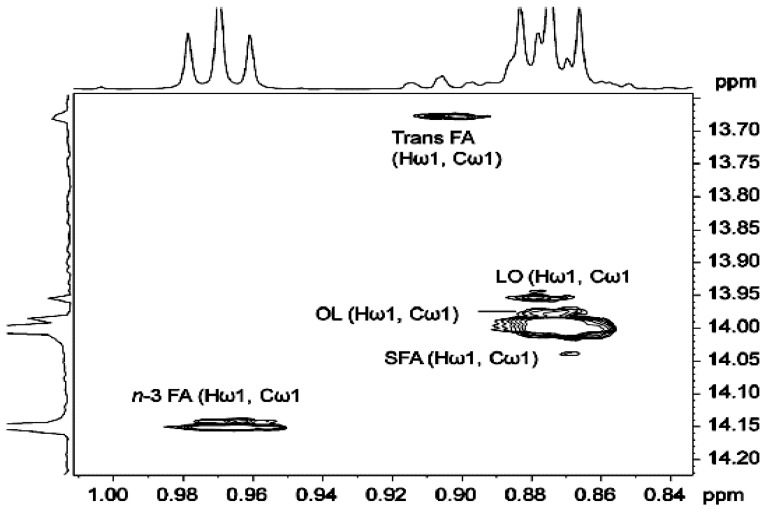
850 MHz band selective ^1^H-^13^C gHSQC spectrum of the aliphatic region of a fish oil supplement. Adopted, with permission, from [60]. Copyright 2015, Royal Society of Chemistry.

Identification and quantification of minor lipids in complex mixtures with the use of ^1^H-^13^C gHSQC experiment suffers from low resolution in the ^13^C dimension. A significant increase in the digital resolution can be achieved with the use of either reduced ^13^C spectra width (Section 5.2) or band selected ^1^H-^13^C gHSQC experiment [86]. Dais et al. [60] utilized band selected ^1^H-^13^C gHSQC experiment to identify the terminal methyl protons of minor *trans* fatty acids in fish oil supplements which indicated a cross peak at δ = 13.72 ppm (Figure 18). This low frequency chemical shift is characteristic of *trans* fatty acids [87]. The achievable resolution was found to be comparable to that of the 1D ^13^C-NMR spectrum in addition to much higher sensitivity due to indirect detection.

### 4.5. Multiplicity Edited ^1^H-^13^C HSQC

**Figure 19:**
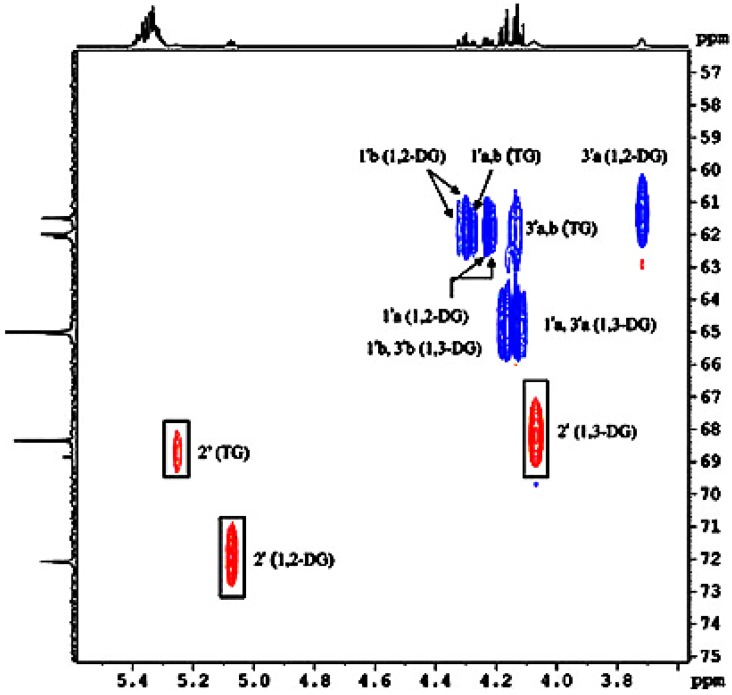
600 MHz HSQC-DEPT NMR spectrum of DAG olive oil in CDCl_3_ solution, showing one bond correlations between the glycerol backbone protons and carbons; negative (blue color) signals for the CH_2_ carbons and positive (red color) signals for the CH carbons. Adopted, with permission, from [59]. Copyright 2011, Springer AOCS.

This is a variant HSQC method which combines both spectra editing (i.e., DEPT-135) and HSQC into one experiment [81,86,88]. The correlation cross peaks are phase coded according to DEPT-135 multiplicity e.g., CH and CH_3_ are of the same positive phase, whereas CH_2_ groups are of negative sign. The method has been successfully applied for the assignment of the ^13^C signals of the glycerol backbone protons of diacylglycerol (DAG) oil (Figure 19). The same experiment was also utilized to assign the methyl, allylic and bis-allylic carbons of linoleic and linolenic acids [59].

### 4.6. 2D ^1^H-^13^C HSQC-TOCSY-Band Selective Experiments

**Figure 20:**
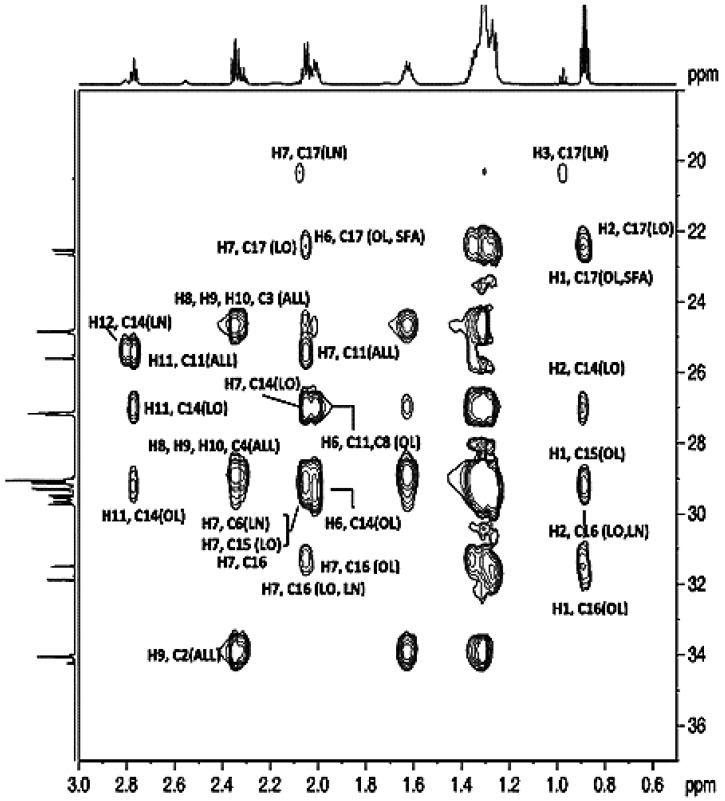
600 MHz gHSQC-TOCSY spectrum of DAG oil in CDCl_3_ solution, showing consecutive connectivities between carbons and protons along a common coupling pathway (for the notation system for ^1^H resonances see the original article). Adopted, with permission, from [59]. Copyright 2011, Springer AOCS.

2D ^1^H-^13^C HSQC-TOCSY is a hybrid NMR experiment which combines the HSQC and TOCSY pulse sequences in order to relay the magnetization along with a proton network, using the greater dispersion of the ^13^C chemical shifts. This experiment is very useful to elucidate assignments in overcrowded ^1^H-NMR spectra since its row, for a particular ^13^C chemical shift, contains a ^1^H TOCSY sub-spectrum of the molecular fragment. The 2D ^1^H-^13^C HSQC-TOCSY experiment has been successfully employed to correlate carbon signals with the adjacent allylic protons of unsaturated fatty acids [89,90] and to analyze the positional distribution of unsaturated chains in TAG model compounds [91]. Of particular interest is the application of gHSQC-TOCSY experiment for mixture analysis in DAG oils [59]. Carbon - proton pairs connected over two bonds were observed for the allylic and bis-allylic carbons of the unsaturated chains which resulted in the unambiguous assignment of several carbons of the acyl chains of oleic, linoleic and linolenic acids (Figure 20). For example, with the use of the pairs H7, C17 of linolenic acid (LN) direct assignment of the allylic carbons C17, C16, C14, C11 and C8 could be achieved with a mixing time of 80 ms (for the notation system for ^1^H resonances see the original article).

A significant increase in the digital resolution in the ^13^C dimension can be achieved with the use of band selective ^1^H-^13^C HSQC-TOCSY experiment. The method has been successfully applied by Willker et al. [86] to investigate multiple ^13^C labeled biofluids and to identify and quantify blood plasma lipids [89] (see Section 5.6.4).

### 4.7. ^1^H-^13^C Heteronuclear Multiple-Bond Correlation (^1^H-^13^C HMBC) Experiments—Band Selective Constant Time ^1^H-^13^C HMBC [80,81,82,83]

The ^1^H-^13^C HMBC experiment detects correlations due to *^n^J*(^1^H-^13^C) couplings, where *n* = 2–4 (possibly *n* > 4 for favorable extended conjugated systems). The method can provide correlations across C–C and C–X fragments, thus, providing one of the most powerful tools for investigating connectivities within extensive molecular skeletons. Due to the variety of *^n^J*(^1^H-^13^C) couplings, the pulse sequence is usually optimized for ~8 Hz.

**Figure 21:**
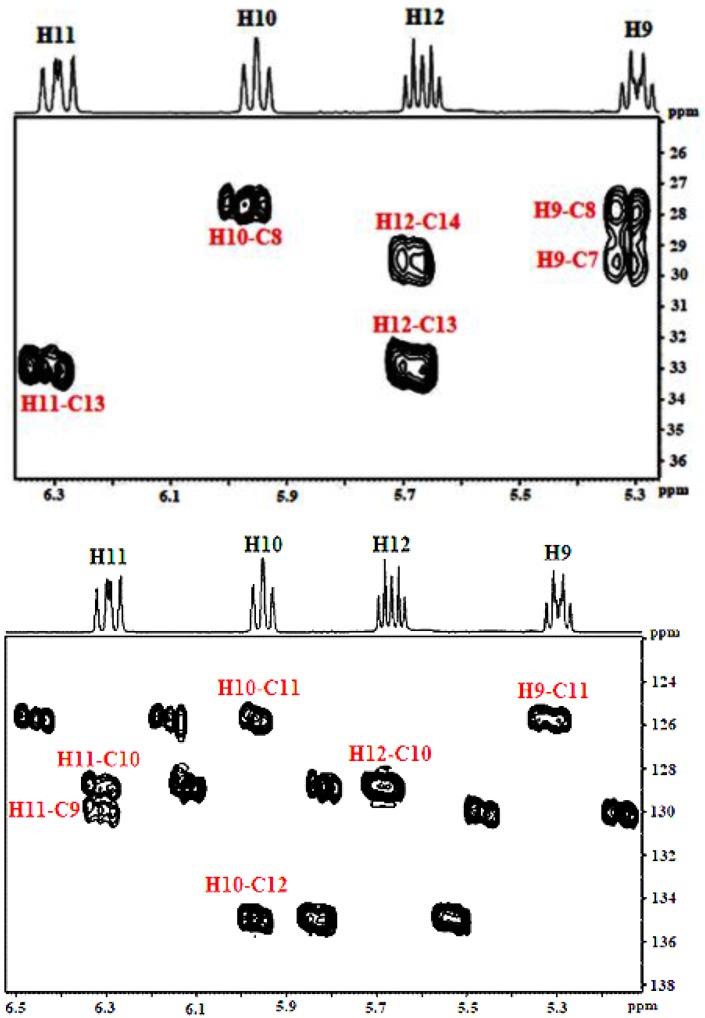
Selected region of 500 MHz 2D ^1^H-^13^C HMBC spectrum of the (9-*cis*, 11-*trans*) 18:2 CLA isomer in CDCl_3_ (solution conditions the same as in Figure 16). Total experimental time = 7 h 41 min.

Figure 21 illustrates selected regions of the ^1^H-^13^C HMBC experiments of the (*cis*-9, *trans*-11) 18:2 CLA isomer in CDCl_3_. The ^1^H signals at 6.28 and 5.93 ppm have been assigned to the protons involved in the olefinic bonds. Μore specifically, the proton at 6.28 ppm correlates with the carbon at 125.3 ppm that was assigned to C11 (Table 19). Similarly, the proton at 5.93 ppm correlates with the carbon at 128.4 ppm (C10). Proton at 5.33 ppm correlates with carbon at 129.6 ppm and, thus, was assigned to the C9. In addition, the H11 (6.28 ppm) shows correlations with the C13 (33.4 ppm), C10 (128.4 ppm) and C9 (129.6 ppm) The H10 (5.93 ppm) correlates with the carbons at 28.1, 125.3 and 134.8 ppm which were assigned to C8, C11, and C12, respectively. The H12 (5.65 ppm) shows correlations with the carbons C14 (29.1 ppm), C13 (33.4 ppm) and C10 (128.4 ppm). The H9 (5.33 ppm) shows correlations with C8 (28.1 ppm), C7 (29.7 ppm) and C11 (125.3 ppm).

^1^H-^13^C HMBC experiments have been successfully applied in lipid mixture analysis. Thus, the gHMBC spectrum of DAG oils [59] provided cross-peaks between protons of the glycerol moiety with the ester CO carbons of the attached fatty acid chain. This allowed the unequivocal identification of the fatty acid which was previously based on additive properties of inductive effects of the double bonds [92]. The authors were able to classify the carbonyl signals into three groups corresponding to 1,3-DAG (173.92–173.82 ppm), 1,2-DAG (173.84–173.30 ppm) and TAG (172.91–172.84 ppm).

**Figure 22:**
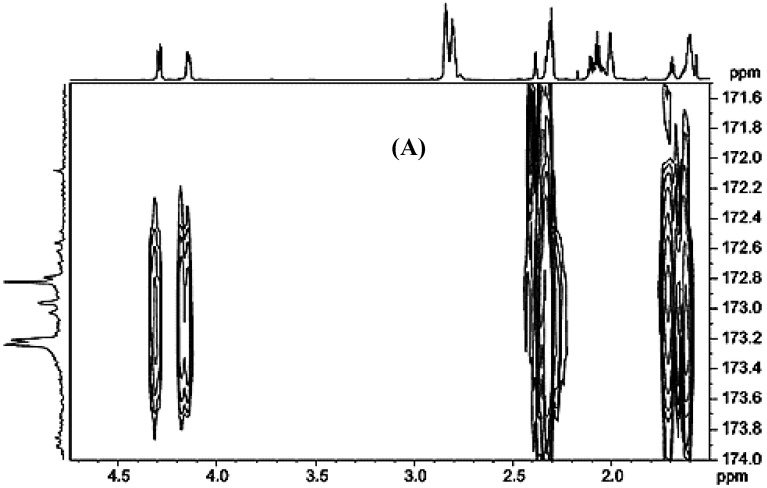

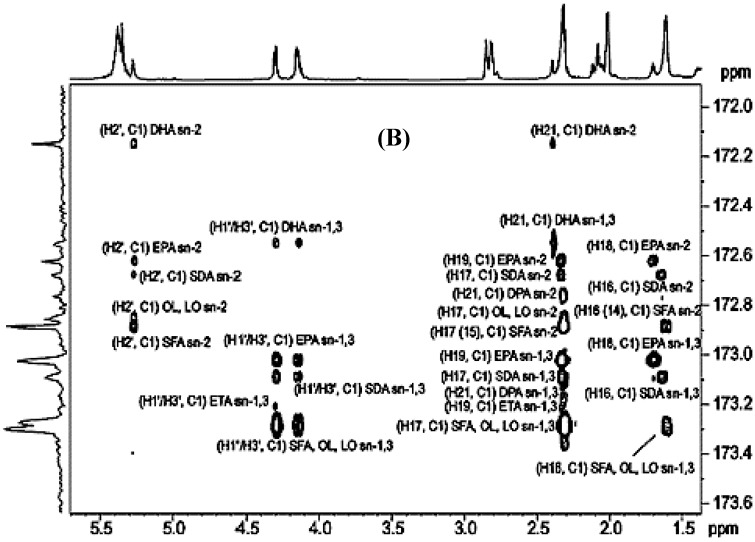
Comparison of the 850 MHz ^1^H-^13^C gHMBC spectrum of a fish oil supplement acquired over a 200 ppm spectral width (**A**) and the band selective constant time HMBC spectrum of the same sample acquired with a 2 ppm spectral width (**B**), in CDCl_3_ solution. Adopted, with permission, from [60]. Copyright 2015, Royal Society of Chemistry.

Band selective constant time ^1^H-^13^C HMBC experiments [93,94] provide a significantly enhanced digital resolution in the ^13^C dimension compared to that of the ^1^H-^13^C gHMBC experiments. Figure 22 illustrates a band selective constant time ^1^H-^13^C HMBC experiment of a fish oil sample using a selective 2 ppm ^13^C spectral width. The excellent resolution of the resulting cross peaks between the carbonyl carbons of various FAs with the glyceridic *sn*-1, *sn*-3 and *sn*-2 positions can provide unequivocal determination of the positional distribution of ω-3, ω-6 and ω-9 fatty acids in the glycerol moiety, including the ω-3 docosapentaenoic acid (DPA) which is an elongated metabolite of EPA (Table 2).

### 4.8. DOSY Experiments

Diffusion-ordered spectroscopy (DOSY) [95] is a sensitive technique that allows the determination of self-diffusion coefficients which are due to random translational motions of molecules as a consequence of their thermal energy. The self-diffusion coefficients, D(m^2^ s^−1^), depend upon molecular properties (size, shape, molecular weight) but, also, on concentration, solvent, temperature and aggregation state. With the DOSY method each component of a mixture can be separated according to its apparent diffusion coefficient and, thus, has found several applications in mixture analysis [96], combinatorial chemistry [95], food chemistry [59,97] and natural products [98,99,100]. In the field of lipid research, the signals of the glycerol segment of TAG were separated relative to those of TAG on the basis of their different diffusion coefficients obtained from the DOSY NMR spectrum of DAG oil [59,60]. The TAG, with larger molecular weight, showed smaller diffusion coefficient (4.5 × 10^−10^ m^2^ s^−1^) than that of the DAG components (5.4 × 10^−10^ m^2^ s^−1^). DOSY experiments were also utilized to obtain qualitative information about the degree of glycerol esterification by the *n*-acyl and *trans* acyl chains [60]. Diffusion-edited NMR techniques, which enhance NMR signals of lipoproteins and remove signals from low molecular weight analytes, have found several applications in body fluids and lipoprotein analysis. This subject will be discussed in Section 5.6.4.

### 4.9. Chemical Shift Reagents (CSR)

Lipid derivatives generally afford NMR spectra that preclude a simple first order interpretation, especially in the case of olefinic, allylic and (CH_2_)_n_ protons (Figure 5 and Figure 6). It has been demonstrated that NMR chemical shift reagents (CSR) such as Eu(fod)_3_ [*tris*-(1,1,1,2,2,3,3-heptafluoro-7,7-dimethyl-4,6-octanedionate)-europium(III)] can increase considerably the amount of structural information obtained from NMR studies [101]. The effect of chemical shift reagents can be considered to be mainly due to dipolar interaction and has been reported to depend on the Lewis basicity of the functional group [102,103]. Thus, the strength of interaction decreases in the order:

amines > alcohols > ketones ≥ aldehydes ≥ ethers > esters > nitriles.

Halides, indoles and double bonds are generally considered to be inactive. Chemical shift studies of methyl oleate revealed that at the optimum [Eu(fod)_3_]/[methyl oleate] ratio of ~1.8, discrete proton signals could only be obtained from C-1 up to C-5 which are near to the coordination site. No induced chemical shifts were observed for the olefinic protons [103]. Information for unsaturated lipid derivatives can be obtained by the introduction of additional functional groups which are CSR active. Thus, successful CSR studies were published on methyl ricinoleate (methyl 12-hydroxy-*cis*-Δ^9,10^-octadecenoate) and methyl 12-hydroxy stearate (methyl 12-hydroxy-9,10-octadecanoate) [104]. For methyl ricinoleate, individual signals were observed for each of the olefinic protons 9 and 10 and their induced shifts were related to the proximity of the OH group. Yb(fod)_3_ has been utilized to identify and discriminate side chain isomers of phytosterols even when using a 90 MHz instrument [105]. For unambiguous assignment of overlapping proton signals it was necessary to perform an incremental CSR study, the construction of proton plots and the calculation of induced shift values.

Despite the original promising results, the CSR method has found limited application in lipid research presumably due to extensive use of high magnetic fields. Nevertheless, Agiomyrgianaki et al. [106] exploited the use of a shift reagent in order to distinguish *cis* and *trans* unsaturation in oils and fats. The method is based on the ability of silver ions to form weak charge-transfer complexes with the olefinic double bonds and the fact that *cis*-unsaturated fatty acids form stronger complexes than the *trans* geometric isomers. The authors were able to differentiate *cis* and *trans* unsaturation in mixtures of *cis* and *trans* methyl esters of monoene aliphatic acids and unsaturated triacylglycerol mixtures, using a low magnetic field instrument (300 MHz for ^1^H). For a mixture of 0.2 M in CDCl_3_ of methyl oleate and methyl elaidate, which have single *cis* and *trans* bonds respectively, very good separation of the mono allylic and olefinic protons at δ = 2.02 ppm and δ = 5.34 ppm was achieved with a silver shift reagent/substrate molar ratio of 0.5. However, attempts to differentiate lipid molecules with different degree of unsaturation and positional distribution of *cis* double bonds were unsuccessful.

### 4.10. Database Matching Approach—Resolving NMR Signals Using Multivariate Data Analysis

Extracts of natural sources consist of complex mixtures of compounds which cannot be effectively and rapidly detected without considerable effort and extensive workup. Thus, the general availability of natural product databases containing experimental data and bioinformatics navigation tolls can greatly facilitate the identification of natural products from complex mixtures [107]. Several NMR databases have been published [108,109]. In the database matching approach, the NMR analysis relies on the spectral comparison using databases of NMR spectra. With this method automatic compound identification can be achieved or the number of possible candidates can be significantly reduced. Among the various data bases, the most popular are the various versions of the Chenomx NMR Suit library (Chenomx, Inc., Edmonton, AB, Canada). Unfortunately, the number of fatty acid standards is, at present, rather limited which precludes unambiguous identification. It should be emphasized that chemical shift variations can occur in lipid extracts with respect to model compounds, due to complex interactions that may occur in the former case and differences in concentration, temperature, solvent etc.

Multivariate data analysis (MDA) has been extensively used to reduce information contained in complex NMR spectra with strongly overlapped peaks. Among the variety of algorithms, the principal component analysis (PCA) has been extensively utilized in order to deconvolute the signals and convert complex systems to simplified and reduced processed information. Recently Pereira et al. [110] applied an unsupervised component analysis to resolve and identify the NMR signals of 3 short-chain fatty acids: acetic acid, propionic acid and butyric acid. The principal object analysis was able to identify the number of independent contributions present in the mixture. The method, however, was applied to a relatively simple mixture, therefore, its utility in complex lipid extracts has yet to be thoroughly investigated.

## 5. Selected Analytical and Structural Studies

### 5.1. Identification and Quantification of Unsaturated Fatty Acids in Complex Mixtures

Omega-3 fatty acids are widely distributed in nature and can be found in walnuts, flaxseed, soybean oil, canola oil, shellfish, salmon, herring, sardins, anchovies, trout, etc. (Table 20 [111]). Omega-6 fatty acids can be found in soybean oil, safflower oil and other liquid vegetable oils. The fatty acid profiles of a variety of fats and oils with emphasis on unsaturated and polyunsaturated fatty acids have been extensively quantified using ^1^H- and ^13^C-NMR [19,20,21,22,25,62,63,112,113,114,115,116,117,118,119,120,121,122]. The ^1^H-NMR method utilizes the integration area per proton and provides equations for determining the mol amounts of the unsaturated fatty acids. Although some modified equations have been suggested depending on the particular fat/oil to be investigated, there is a general consensus that ^1^H-NMR can provide an excellent method for obtaining quantitative results on TAG, 1,2-DAG, DHA, α-linolenic acid, linoleic acid and UFA.

Evaluation of the total amount (%) of triacylglycerides (TAG) can be based on the integrals of the signals due to –CH_2_–OOC– protons of the glycerol moiety at ~4.30 ppm (I_4.30_) using the following equation [123]:

(1) $$\mathrm{TAG}\;\left(\%\right)=\frac{I_{4.30}}{\;\frac{I_{2.46}}{2}+I_{2.33}\;}\times \;100.$$

where I_2.46_ and I_2.33_ are the integrals of the COOH–CH_2_–CH_2_– protons of DHA acid and –OOC–CH_2_– CH_2_– protons of all fatty acids.

Evaluation of the total amount (%) of 1,2-diacylglycerides (1,2-DAG) can be based on the integral of the HO–CH_2_–CH– group of the glycerol moiety at ~3.72 ppm (I_3.72_) using the following equation:

(2) $$1,\;2-\mathrm{DAG}\;\left(\%\right)=\;\frac{I_{3.72}}{\;\frac{I_{2.46}}{2}+I_{2.33}\;}\times 100.$$

Evaluation of the total amount (%) of docosahexaenoic acid (DHA) can be based on the integral of the COOH–CH_2_–CH_2_– protons at ~2.46 ppm (I_2.46_) using the following equation:

(3) $$\mathrm{DHA}\;\left(\%\right)\;=\frac{\frac{I_{2.46}}{2}}{\frac{I_{2.46}}{2}+I_{2.33}}\;\times \;100.$$

Alternatively, evaluation of the DHA can be based on the integral of the =HC–CH_2_–CH= protons of the DHA at ~2.85 ppm (I_2.85_) as follows:

(4) $$\mathrm{DHA}\;\left(\%\right)=\frac{3{\;I}_{2.85}}{6\;I_{0.89-1}}\times \;100$$

where I_0.89–1_ is the total integral of the –CH_3_ group of α-linolenic acid at 0.98 ppm and of the rest of the fatty acids at ~0.89 ppm.

Evaluation of the total amount (%) of α-linolenic acid was based on the integral of the =CH–CH_2_–CH= protons at ~2.82 ppm (I_2.82_) using the following equation:

(5) $$\alpha -\mathrm{linolenic}\;\left(\%\right)=\frac{3{\;I}_{2.82}}{4{\;I}_{0.89-1}}\times \;100.$$

Alternatively, evaluation of the α-linolenic acid can be based on the integral of the –CH_3_ group at ~0.98 ppm (I_0.98_) as follows:

(6) $$\alpha -\mathrm{linolenic}\;\left(\%\right)=\frac{2{\;I}_{0.98}}{3\;\left(\;\frac{I_{2.46}}{2}+I_{2.33}\;\right)}\;\times \;100.$$

Evaluation of the total amount (%) of linoleic acid was based on the integral of the =CH–CH_2_–CH= protons at ~2.78 ppm (I_2.78_) using the following equations:

(7) $$\mathrm{linoleic}\;\left(\%\right)=\frac{I_{2.78}}{\;I_{2.33}}\times \;100\;\mathrm{and}$$

(8) $$\mathrm{linoleic}\;\left(\%\right)=\frac{3{\;I}_{2.78}}{2{\;I}_{0.89-1}}\times \;100.$$

Evaluation of the total amount (%) of UFA was based on the integrals of the signals due to –CH_2_–CH=CH– protons at ~2.02 ppm (I_2.02_) as follows:

(9) $$\mathrm{UFA}\;\left(\%\right)=\frac{2{\;I}_{2.02}}{4\;\left(\;\frac{I_{2.46}}{2}+I_{2.33}\;\right)}\times \;100\;\mathrm{and}$$

(10) $$\mathrm{UFA}\;\left(\%\right)=\frac{3{\;I}_{2.02}}{4{\;I}_{0.89-1}}\times \;100.$$

**Figure 23:**
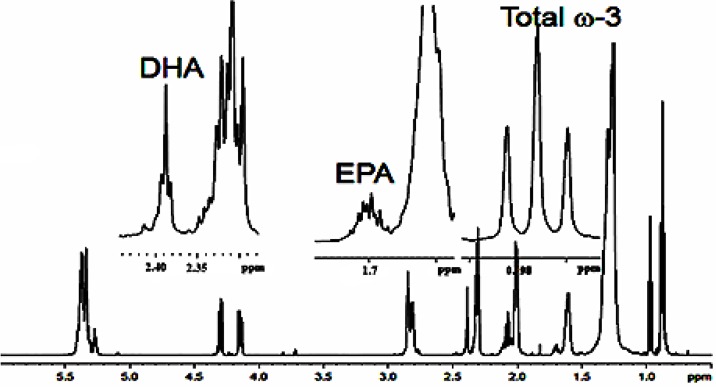
^1^H-NMR spectrum of a salmon oil which allows the simultaneous detection and quantification of ω-3, EPA and DHA. Free download from: http://www.ilps.org/index.php/Standardized_Methods.html.

Evaluation of the degree of total unsaturation in oils and fats with ^1^H-NMR (Equations (9) and (10)) has distinct advantages with the respect to the classical iodine value since it is more rapid and can provide information on the different types of unsaturated fatty acids [124]. Quantification of ω-6 and ω-3 polyunsaturated fatty acids can also be carried out by ^1^H-NMR using the above equations in a simple and efficient way compared to classical analytical methods which involve *trans*-esterification of the oil or fat to produce methyl esters and the identification and quantification by gas chromatography [125]. The conversion of ^1^H-NMR data from mol % to wt % can be achieved [124] with the hypothesis that the average chain lengths of mono- and poly- unsaturated are C_18_ and by estimating the average number of protons in unsaturated and saturated chains from the integrals of given resonances in the spectrum. Figure 23 illustrates the ^1^H-NMR spectrum of a salmon oil. The amount of total ω-3 can be easily calculated using the integral of the triplet at 0.97 ppm. Quantification of the individual DHA and EPA fatty acid can also be obtained on the basis of the integrals at 2.40 ppm, due to CH_2_–CH_2_–COOR protons of DHA, and, at 1.7 ppm, due to CH_2_–CH=CH protons of EPA.

**Figure 24:**
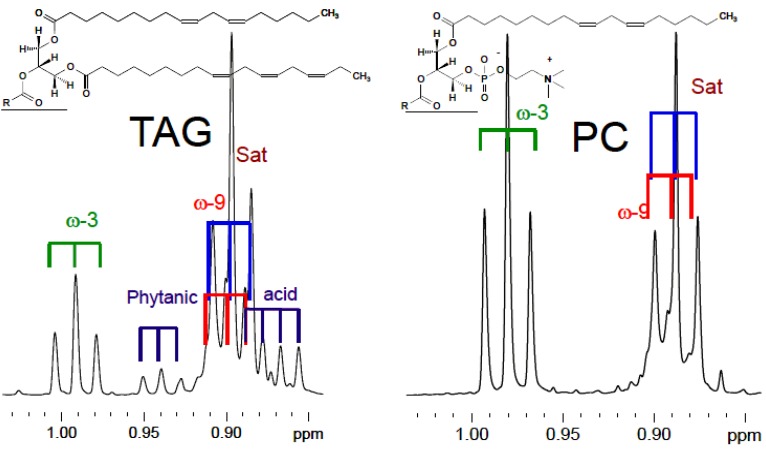
^1^H-NMR spectra of TAG and phospholipids from krill oil after preparative separation. The neutral lipids fraction contains the branched phytanic acid which can also be quantified. Free download from: http://www.ilps.org/index.php/Standardized_Methods.html.

Figure 24 illustrates the ^1^H-NMR spectra of TAG and phospholipid fractions from krill oil after preparative separation. The identification and quantification of ω-3 from ω-6, ω-9 and saturated fatty acids can be achieved. Interestingly, the branched phytanic acid can also be quantified.

### 5.2. Identification and Quantification of Minor Lipids in Complex Mixtures

**Figure 25:**
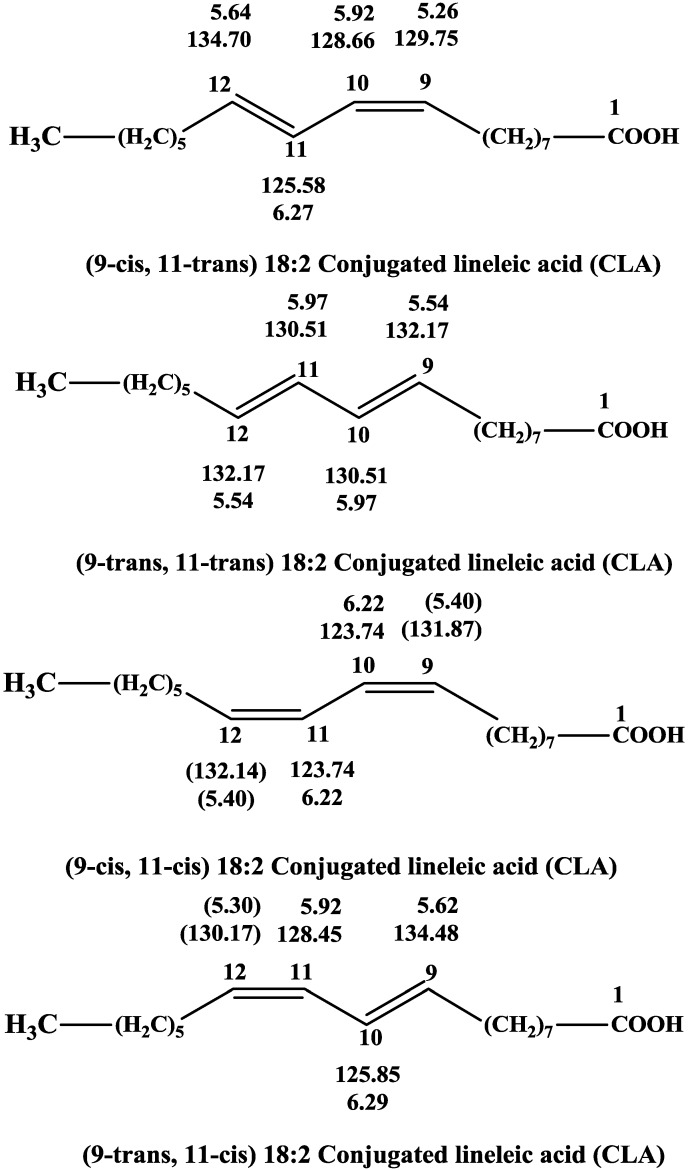
^1^H- and ^13^C-NMR chemical shifts, in ppm, of C9 to C12 carbons and their attached protons for the four geometric isomers of 18:2 CLA. Adopted, with permission, from [56]. Copyright 2014, Elsevier B.V.

**Figure 26:**
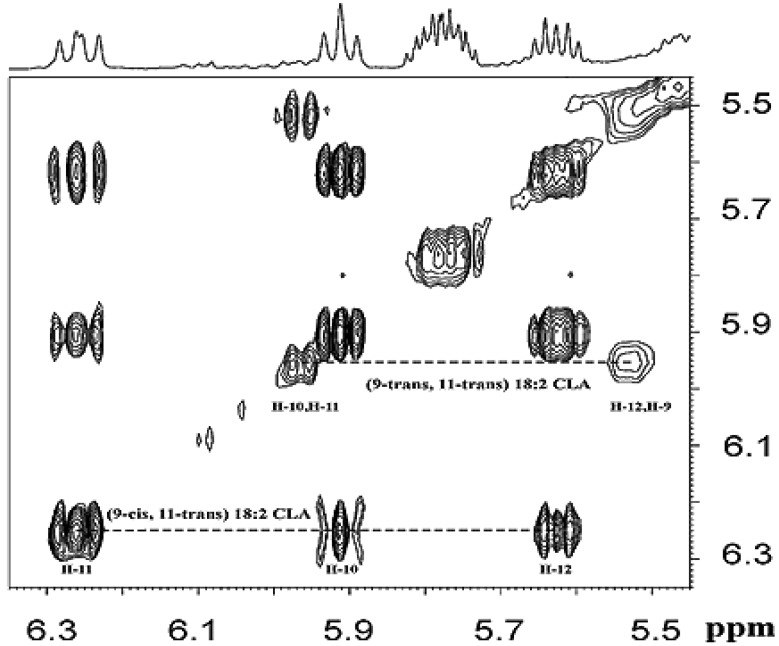
500 MHz ^1^H-^1^H TOCSY NMR spectrum of the lipid fraction of a lyophilized milk sample in CDCl_3_ with suppression of two major lipid proton resonances at 1.27 and 5.35 ppm. Experimental conditions: 298 K, 32 repetitions of 256 increments, total experimental time 5 h 5 min. Adopted, with permission, from [56]. Copyright 2014, Elsevier B.V.

**Figure 27:**
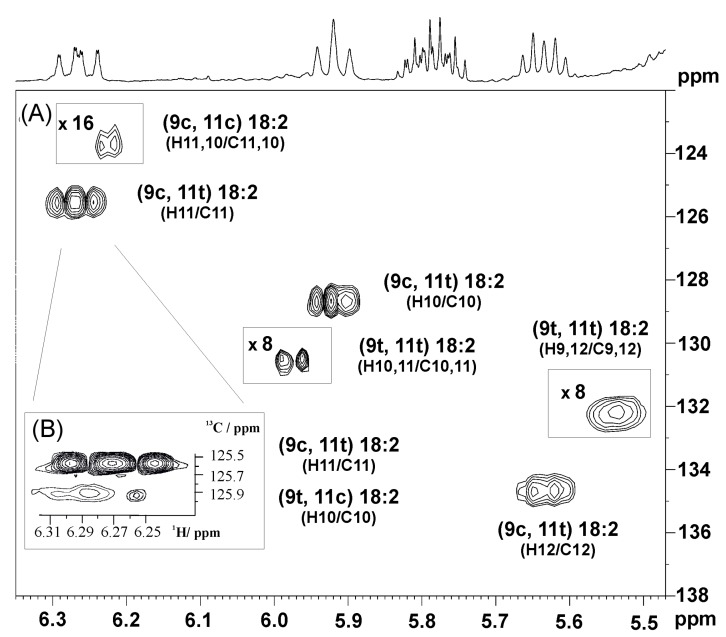
Selected regions of 500 MHz ^1^H-^13^C HSQC spectra of the lipid fraction of a lyophilized milk sample of Figure 26. (**A**) With ^13^C shielding spectral range from 0 to 160 ppm and (**B**) with reduced ^13^C spectral range from 112 to 142 ppm. Experimental conditions: 298 K, 40 repetitions of 256 increments, total experimental time 4 h 55 min and 5 h 49 min for (**A**,**B**), respectively. Adopted, with permission, from [56]. Copyright 2014, Elsevier B.V.

The analysis of minor lipids is extremely challenging and complex because it can be very slow and laborious using the classical chromatographic analytical methods. Tsiafoulis et al. [56] reported direct identification and quantification of four minor geometric (9-*cis*, 11-*trans*) 18:2, (9-*trans*, 11-*cis*) 18:2, (9-*cis*, 11-*cis*) 18:2 and (9-*trans*, 11-*trans*) 18:2 conjugated linoleic acid (CLA) isomers (Figure 25) in lipid fractions of lyophilized milk samples with the combined use of 2D ^1^H-^1^H TOCSY (Figure 26) and 2D ^1^H-^13^C HSQC (Figure 27). Selective saturation of major lipid signals, resulted in an increase in the S/N of the CLA resonances by a factor of three due to the significantly higher receiver gain of the spectrometer (Section 4.1). This allowed the identification of both the (9-*cis*, 11-*trans*) 18:2 CLA and the minor (9-*trans*, 11-*trans*) 18:2 CLA.

Further confirmation of the above assignments was achieved with the use of ^1^H-^13^C HSQC experiment (Figure 27). Thus, the near generated H10 and H11 protons at 5.97 ppm of (9-*trans*, 11-*trans*) 18:2 CLA isomer indicated a ^13^C cross peak at 130.51 ppm which should be compared with the literature ^13^C values of 130.37 and 130.51 ppm for carbons, respectively. Moreover, the H10 and H11 resonances at 6.22 ppm that correlate to the C10 and C11 carbons at 123.74 ppm are most likely due to the presence of (9-*cis*, 11-*cis*) 18:2 CLA isomer. Decrease of the spectral width in the ^13^C dimension from ~143 ppm to ~30 ppm resulted in a significant increase in the digital resolution. Thus, in addition to the major resonance at 6.27 ppm with a cross peak at 125.58 ppm, due to (9-*cis*, 11-*trans*) 18:2 isomer, the minor resonance at 6.29 ppm with a cross peak at 125.85 ppm was tentatively designed to H10-C10 connectivities of the (9-*trans*, 11-*cis*) 18:2 isomer.

The determination of the concentration of the CLA isomers was based on the combination of 1D ^1^H-NMR and 2D ^1^H-^13^C HSQC described by Lewis et al. [126] on the basis of calibration curves from known concentrations of the geometric isomers. The integral of each isomer was normalized with the respect to the signal of the reference compound TSP-*d*_4_ in DMSO-*d_6_*_,_ of known concentration, in a coaxial arrangement.

Although the reduced ^13^C spectra width is a useful method to increase digital resolution in the ^13^C dimension in 2D ^1^H-^13^C HSQC experiments, complications may arise due to folding resonances. As demonstrated by Dais et al. [60] band-selective ^1^H-^13^C HSQC and ^1^H-^13^C HMBC experiments can provide excellent resolution in the ^13^C dimension comparable to that obtain with 1D ^13^C-NMR (Figure 18 and Figure 22).

**Figure 28:**
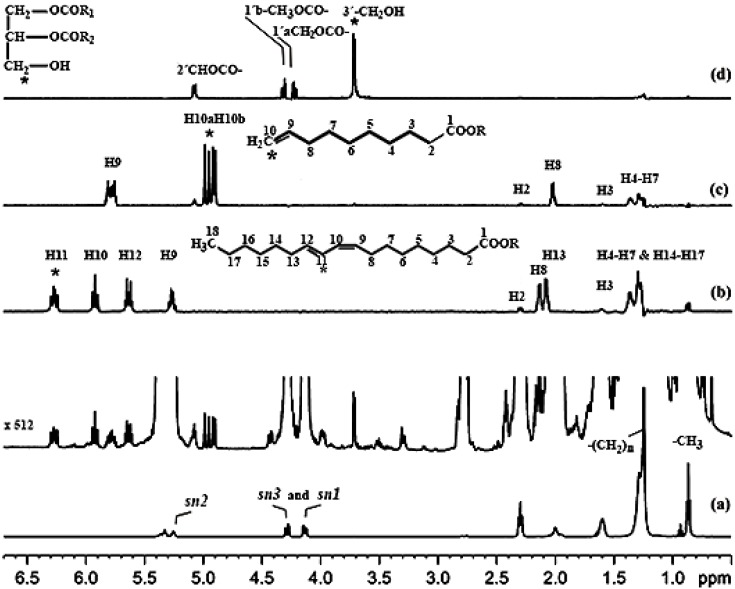
Spin chromatogram of the lipid fraction of a lyophilized cheese sample in CDCl_3_. (**a**) 500 MHz ^1^H-NMR spectrum of the lipid fraction of a lyophilized cheese sample in CDCl_3_ (T, 298 K; number of scans, 256; acquisition time, 4.3 s; relaxation delay, 5 s; and total experiment time, ~25 min). The major lipid resonances are denoted (*sn*1, *sn*2, and *sn*3 indicate the stereospecific numbering of esterified glycerol). The inset shows 512× magnification of the spectrum to display resonances from the 18:2 CLA and other minor species. (**b**–**d**) 1D TOCSY spectra of panel with τ_m_ = 400 ms (number of scans, 256; and total experiment time, ~25 min). The asterisks denote the resonances that were excited by the use of a selective pulse. Adopted, with permission, from [78]. Copyright 2015, American Chemical Society.

Recently the direct identification of six minor species in the lipid fraction of milk and halloumi cheese, (9 *cis*, 11-*trans*) 18:2 and (9-*trans*, 11-*trans*) 18:2 CLA isomers, caproleic acid (CA), glycerol in 1,2-diacylglyceride (1,2 DAG), 1-monoacylglyceride (1-MAG), and 2-monoacylglyceride (2-MAG), was achieved with the use of selective one-dimensional total correlation spectroscopy (1D-TOCSY). Figure 28 illustrates a 500 MHz ^1^H-NMR spectrum of the lipid fraction of a lyophilized halloumi cheese sample in CDCl_3_. Selective 1D TOCSY experiment, with 400 mixing time of the H11 proton of the (9-*cis*, 11-*trans*) 18:2 CLA isomer illustrates an effective magnetization transfer throughout the full spin system, although the signals of the H2 to H8 and H12 to H18 were completely hidden in the conventional 1D ^1^H-NMR spectrum under the resonances of the abundant TAG analytes with signal intensities stronger by a factor of 4 × 10^2^ to 3 × 10^3^. Similar experiments were performed with the selective excitation of the H10a H10b protons at 4.97 ppm of caproleic acid (Figure 28c) and at 3.72 ppm of the 3′-CH_2_OH proton of the glycerol moiety in 1,2 DAG (Figure 28d).

In all cases the 1D TOCSY experiment allows the complete identification of minor lipids in a time-efficient manner and with excellent resolution. The 1D TOCSY quantification was based on the standard addition method of Sandusky et al. [127]. More specific successive amounts of e.g., 0.5, 1.0 and 2.0 mM of caproleic acid were added and the resulting linear correlation was utilized for the estimation of the concentration of caproleic acid. Table 21 provides the quantification data which are in agreement with those obtained with the use of the GC-MS method of analysis (ISO 15884: 2002) [128].

Selective 1D TOCSY excitation of the bis-allylic protons at 2.82 ppm demonstrated the effective magnetization transfer to the proton of methyl group at 0.98 ppm. Similar results were obtained with selective 1D TOCSY excitation of the –CH_3_ group at 0.98 ppm which demonstrated the effective magnetization transfer to the bis-allylic protons at 2.82 ppm with complete elimination of the bis-allylic protons of linoleic acid at 2.78 ppm. This provided assignment that the resonances at 2.82 and 0.98 ppm should be attributed to the α-linolenic acid [123].

A selective 1D TOCSY excitation for the assignment of the weak bis-allylic protons at 2.85 ppm demonstrated not only an effective magnetization transfer to the –CH_3_ group at 0.98 ppm and, thus, its assignment as an ω-3 lipid, but also to a very characteristic complex resonance at 2.46 ppm. This provided an unequivocal assignment that the ω-3 lipid is the docasahexaenoic acid (DHA). These experiments clearly demonstrated that the 1D TOCSY allows for the unequivocal assignment and structure elucidation of ω-3 and ω-6 lipids in a time-efficient manner and with excellent spectral resolution [123].

**Figure 29:**
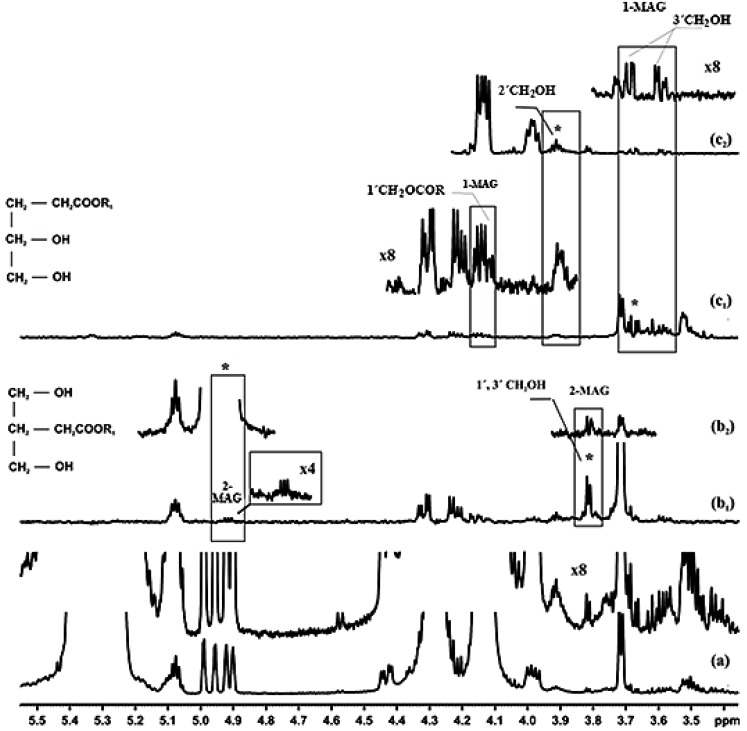
Selected regions of (**a**) Figure 28a, (**b**) 1D TOCSY spectrum that demonstrates the spin system of the glycerol moiety in 2-MAG (in panels b1 and b2, the selective excitation pulse was set on the 1′,3′-CH_2_OH (δ = 3.82 ppm) and 2′-CHOCOR (δ = 4.92 ppm) peaks in 2-MAG, respectively), and (**c**) 1D TOCSY spectrum of the spin system of the glycerol moiety in 1-MAG (in panels c1 and c2, the selective excitation pulse was set on the 3′-CH_2_OH (δ = 3.67 ppm) and 2′-CH_2_OH (δ = 3.92 ppm) peaks in 1-MAG, respectively). Adopted, with permission, from [78]. Copyright 2015, American Chemical Society.

**Figure 30:**
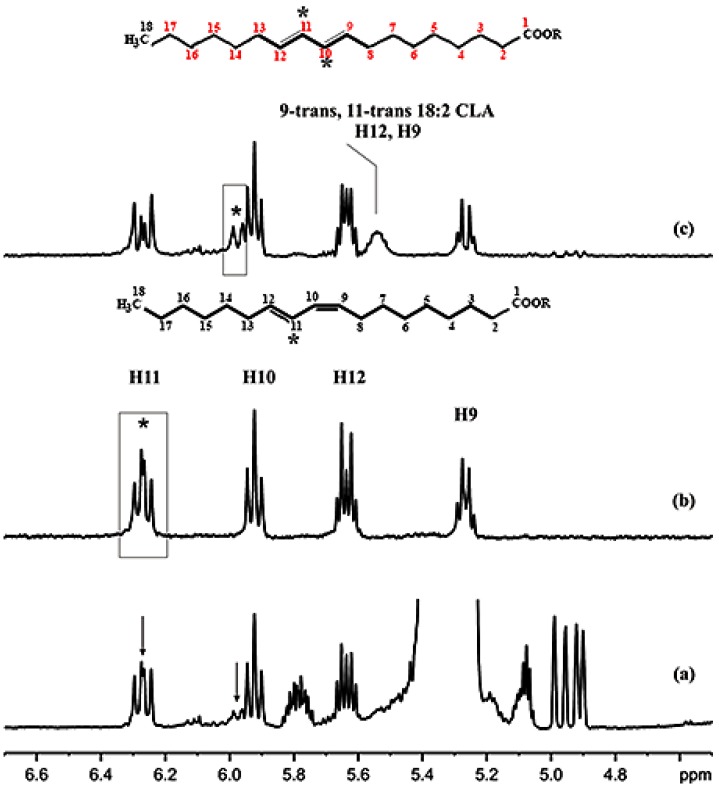
(**a**) Selected region of the 1D ^1^H-NMR spectrum of the solution of Figure 28a. The asterisks denote the position of the selected target resonances which were excited. (**b**,**c**) selective 1D TOCSY spectra of the above solution. In (**b**) τ_m_ = 400 ms was used for magnetization transfer from H11 to H9 of the (9-*cis*, 11-*trans*) 18:2 CLA. In (**c**) τ_m_ = 70 ms was used in order to optimize magnetization transfer from H10, 11 to H9, 12 of the (9-*trans*, 11-*trans*) 18:2 CLA. The asterisks denote the resonances that were excited with the use of a selective pulse.

Figure 29 illustrates the potential of 1D TOCSY experiment in the case of the minor components of 2-MAG and 1-MAG. The spin systems of the glycerol moieties of 2-MAG and 1-MAG were unequivocally assigned, although the 1-MAG resonances are strongly overlapped in the region of 3.75 to 3.55 ppm. The 1D TOCSY experiment can be applied even in cases of strongly overlapped resonances. Figure 30 illustrates an apparent triplet at 5.92 ppm (^3^*J* = 10.9 Hz) of the H10 olefinic proton of the (9-*cis*, 11-*trans*) 18:2 CLA which is strongly overlapped with a minor peak at 5.97 ppm which has been assigned to the composite signal of the H10 and H11 protons of the (9-*trans*, 11-*trans*) 18:2 CLA isomer. Selective excitation of the minor peak at 5.97 ppm clearly demonstrates magnetization transfer to the composite signal of the H9 and H12 resonances at 5.54 ppm which are not overlapped with other resonances of the lipid milk fraction.

### 5.3. Identification and Quantification of Free Fatty Acids

Free fatty acids (FFAs) in oils, waxes and various pharmaceutical excipients [129] arise from several processes including: (i) the hydrolysis of triacylglycerides (TAGs) during production and storage of the oil, (ii) handling of the row material and (iii) secondary oxidation of unsaturated aldehydes or other oxidation products originating from the cleavage of lipid hydroperoxides resulting in the formation of short chain FFAs [130].

Satyarthi et al. [131] utilized the integration of the α-CH_2_ methylene protons to quantify free fatty acids in non-edible lipids and biodiesel. The method is not sufficiently sensitive to detect small content of FFA in lipids due to the presence of a strong signal from the α-CH_2_ protons of esterified groups. Recently, Skiera et al. [129,132] utilized the integration of the free carboxylic group which is extremely deshielded. The method was applied to 305 oil and fat samples. Application to castor oil was not successful due to extensive line broadening which may be attributed to exchange with the OH protons of alcohol groups. It was, therefore, necessary to use mixtures of CDCl_3_/DMSO-*d*_6_ to reduce exchange rates and, thus, line widths. Non-protic hydrogen bonding organic solvents, especially DMSO-*d*_6_, have been extensively utilized in the reduction of proton exchange rates of OH groups in natural products [133,134,135,136]. ^31^P-NMR has been utilized to investigate FFA content in lipids, however, the method requires the derivatization of FFAs prior to ^31^P-NMR analysis [137,138,139]. It should be emphasized that the most successful method for the identification and quantification of FFAs is ^13^C-NMR in the carboxyl and ester carbonyl region (Section 3.2.1) despite the long experimental times required [140,141].

### 5.4. Investigation of Oxidation Products 

The problem of oxidative deterioration is of great economic importance in the production of lipid containing foods. Oxidation of unsaturated lipids not only produces offensive odours and flavours but can also decrease the nutritional quality and safety due to the formation of secondary reaction products. Even for a simple monounsaturated lipid, such as oleate, the autoxidation may lead to a variety of hydroperoxides and terminal products. NMR spectroscopy is regarded as one of the most reliable methods to study lipid peroxidation for two reasons: (i) it can investigate changes in the starting materials as well as the evolution of various oxidation products during the process, and (ii) does not require any chemical reaction, contrary to various spectrophotometric techniques [26,28,29].

**Figure 31:**
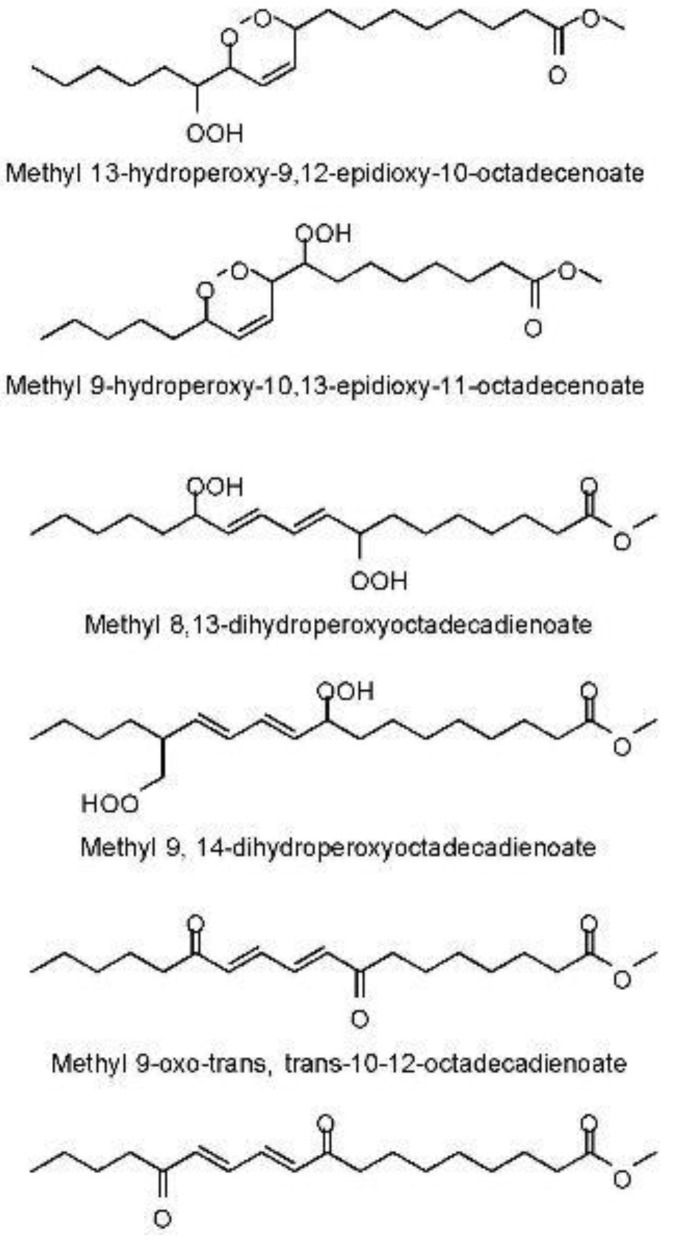
Oxidized products of methyl linoleate. Adopted, with permission, from [143]. Copyright 1983, Oil Chemists’ Society (AOCS).

^1^H-NMR spectroscopy has been extensively utilized to elucidate the structures of primary and secondary products of lipid peroxidation [26,28,29]. Chan et al. [142] investigated the oxidation of methyl linoleate for 48–72 h at 30 °C and elucidated, after HPLC analysis, the structures of four major oxidized products by using a low field (90 MHz) NMR spectrometer. Similarly, Neff et al. [143], elucidated the structures of oxidized products from photoxidation of methyl linoleate (Figure 31). It should be emphasized that the six membered epidioxides undergo hydrogen abstraction, loss of O_2_ and rearrangement of double bonds to form other oxidized products of methyl linoleate.

^1^H-NMR was used by Saito [144] to determine the extent of lipid peroxidation of fish oil stored at 40 °C. A significant difference in the peak area of olefinic protons compared to the aliphatic protons was observed, which is a strong indication of oxidation. The ratios of aliphatic to olefinic protons and aliphatic to diallylmethylene protons were found to gradually increase during the oxidation of lipids and may serve as an index of oxidative degradation of oil samples [145].

Grootveld et al. [146,147,148] investigated in detail lipid oxidation products in autoxidized culinary oils rich in PUFAs which were subjected to standard frying/cooking practices. With the use of various 2D ^1^H-^1^H and 2D ^1^H-^13^C-NMR techniques the detection and quantification of *trans*-2-alkenals, *trans*-*trans*, and *cis*-*trans*-alka-2,4-dienals and *n*-alkanals were achieved.

Goicoechea and Guillen [149], have reported a detailed study of primary and secondary oxidized products of corn oil being oxidized at room temperature and provided chemical shifts and splitting patterns for a variety of oxidation products which are of importance in analysing lipid oxidation. For example, the aldehyde proton resonates at 9.3–10 ppm and a doublet signal indicates an adjacent CH group. Therefore, the doublet signal at 9.49 ppm can be regarded as (*E*)-2-alkenal. On the other hand, the triplet signal at 9.75 indicates an adjacent CH_2_ group and may be regarded as the aldehyde proton from alkanals.

Guillen et al. [26] comprehensively reviewed the use of NMR techniques to study the thermal oxidation of food lipids. In addition to thermal oxidation, the concentration and nature of the compounds formed were also described. Table 22 presents chemical shifts and multiplicities of the ^1^H-NMR signals in CDCl_3_ of some primary and secondary (or further) oxidation compounds generated in edible oils and model lipid compounds submitted to different degradative conditions [149,150,151,152,153,154,155,156,157,158,159,160,161,162,163]. These include various types of aldehydes, ketones, alcohols and epoxides. The formation of these secondary oxidized compounds depends upon composition of the original food lipid and thermo-oxidation conditions used.

**Figure 32:**
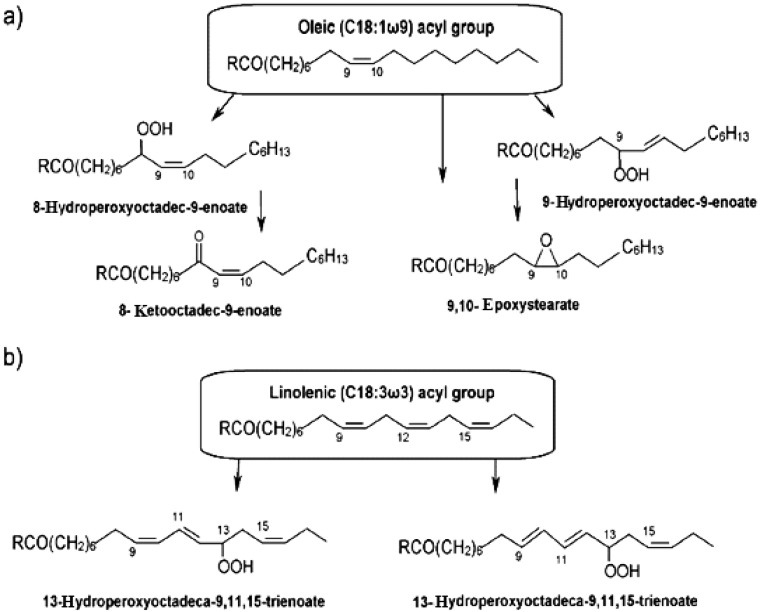
Some of the primary oxidation compounds derived from (**a**) oleic and (**b**) linolenic acyl groups, that can be detected by ^1^H-NMR. Adopted, with permission, from [26]. Copyright 2014, Institute of Food Technologists.

**Figure 33:**
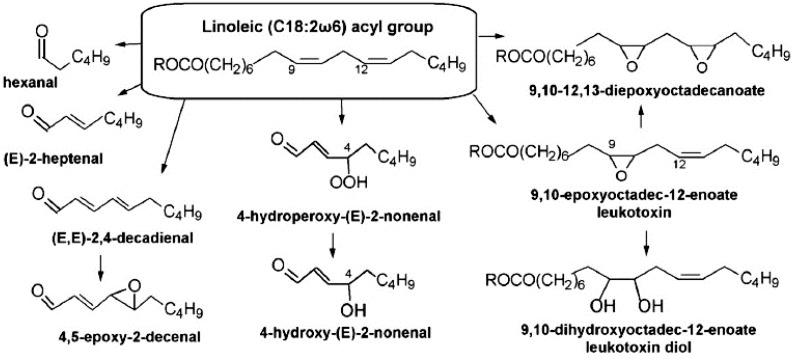
Some of the secondary or further oxidation compounds derived from linoleic acyl groups that can be detected by ^1^H-NMR. Adopted, with permission, from [26]. Copyright 2014, Institute of Food Technologists.

It is worth noticing that aldehydes are, generally, considered to be the secondary oxidation products, all α,β-unsaturated aldehydes have conjugated double bonds and few of them have hydroperoxy groups as well. This indicates that neither conjugated diene nor the hydroperoxy group are exclusive primary oxidation products. Figure 32 illustrates some primary oxidation products derived from unsaturated oleic and linolenic acyl groups. Figure 33 illustrates secondary and further oxidation products derived from linolenic acyl groups.

Skiera et al. [164] suggested a ^1^H-NMR method for the quantification of hydroperoxides in edible oils. Proton transfer line width broadening of the hydroperoxide (OOH) signal was significantly eliminated using a mixture of CDCl_3_ and DMSO-*d*_6_ (5:1 *v*:*v*) and, thus, accurate integration could be obtained. The method was applied to 200 edible oil samples and the analytical performance was compared with the commonly used method of peroxide value (PV). Significant discrepancies between the two methods for some oil varieties were observed. The low hydroperoxide values in the case of olive oils compared with the NMR values were attributed to the high content of specific phenolic compounds which exhibit strongly deshielded ^1^H-NMR chemical shifts in the region of hydroperoxide signals [165,166,167].

**Figure 34:**
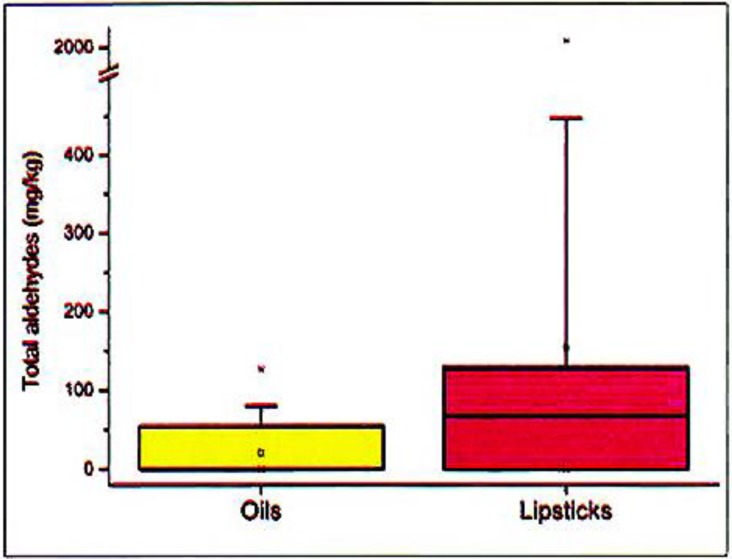
Total aldehydes in 72 oils and 38 cosmetics. The boxplot shows a statistical distribution whiskers: minimum and minimum (max 1.5 times the length of the inner quartiles; data points outside are outliers). Adopted, with permission, from [168]. Copyright 2010, John Wiley & Sons Ltd.

**Figure 35:**
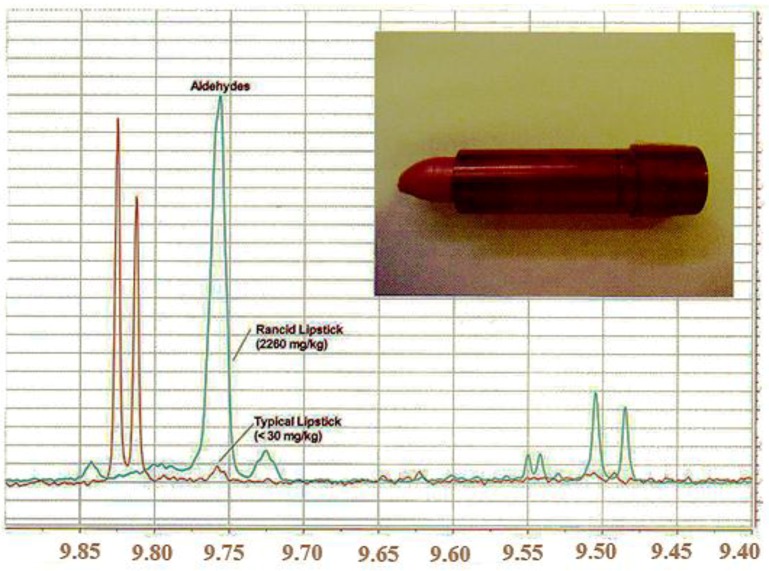
NMR spectrum of an authentic rancid lipstick sample (shown in insert) compared to a non-oxidized sample. Adopted, with permission, from [168]. Copyright 2010, John Wiley & Sons Ltd.

Lachenmeier et al. [166] utilized the aldehyde proton resonances in the region of 9–10 ppm for quality control of vegetable oils and decorative cosmetics. The method was used to analyze 72 oil and 38 cosmetic samples in order to investigate their compliance with food and cosmetics regulations. 22 samples (31%) from the vegetable oil, contained detectable levels of aldehydes. Only two samples contained levels above 100 mg kg^−1^ (Figure 34). 26 samples (68%) from the analyzed 38 lipsticks were found to contain aldehydes above the detection limits and 15 had levels above 100 mg kg^−1^. One sample, which was characterized by a rancid state, was found to contain over 2000 mg kg^−1^ of total aldehydes (Figure 35). It was concluded that lipsticks are problematic with regard to oxidative stability presumably due to relatively high temperatures (>75 °C) used and the presence of pro-oxidants, such as iron oxides, which may be contained as pigments.

### 5.5. LC-NMR

LC-NMR is one of the most powerful methods for separation and structural elucidation of unknown compounds in mixtures [169,170,171,172,173]. LC-NMR, thus, represents a potentially interesting complementary technique to LC-MS in analysis of complex biological matrices for the detailed on-line structural analysis of products. However, there is a very limited number of recent publications about applications that can demonstrate the usefulness of this technique in lipid analysis.

LC-NMR was used for the quantification of α- and γ-linolenic free fatty acids resulting from blackcurrant (*Ribes nigrum*) hydrolysis reaction [174]. A 2D reverse phase HPLC method with two HPLC columns, C_8_ and C_18_ in a series, was utilized. The results were found to be in agreement with the GC method on FAME.

### 5.6. Lipidomics

Lipidomics is a subfield of metabolomics that focuses on the study of molecular lipids within a cell, tissue, and biofluids, including the analysis of lipid species and their abundance as well as studies of their biological activities, subcellular localization, and tissue distribution [175,176,177]. The most commonly applied analytical methods for lipidomics are based on MS, often combined with chromatographic separation methods such as LC and GC. Several hundreds of lipids can be separated with the UPLC-MS methodologies in the profiling studies of various biological matrices. For sufficiently volatile lipids, GC-MS and GC coupled to flame ionization detection is the most widely used method for the analysis of FAs, both for FFAs and esterified FAs. Nevertheless, a significant number of studies have used NMR in the field of lipidomics including composition and authenticity of oils, quality and identification of dairy products and their provenance and geographical origin quality, and lipoprotein analysis in serum/plasma. Some representative examples will be analyzed below.

#### 5.6.1. Authentication and Quality Assessment of Oils

Extra virgin olive oil is the most investigated oil due to its high quality nutritional and sensorial properties and, thus, high commercial value. This frequently leads to adulterations with low quality oils of different botanical origin or refined olive oils. Furthermore, the analysis and monitoring of olive oils of specific protected geographical origin is a challenging analytical problem for NMR spectroscopy because there is no single physicochemical parameter which allows a comprehensive investigation [178,179]. Therefore, emphasis has been given to food fingerprinting with the intrinsic aim to detect as many compounds in the metrics as possible. Mannina and Sobolev provided a comprehensive overview of high resolution NMR characterization of Mediterranean olive oils in terms of quality, authenticity and geographical characterization [180]. Dais and Hatzakis investigated, with the use of NMR, the geographical and varietal classification of extra virgin oil [181] and provided a comprehensive overview of NMR methods for quality control and authentication of olive oil through metabolic profiling or fingerprinting [182].

^1^H-NMR investigations of a large number of oil samples and subsequent PLS-DA statistical analysis demonstrated the correct, up to 90%, sample classification/prediction [183,184,185]. ^1^H NMR was utilized to predict the geographical origin of olive oil samples (three regions from Italy and four regions from Greece) with a use of data bucketing and PSA and by a Monte Carlo embedded cross-validation [73]. Due to the rather limited number of samples which were investigated, correct prediction probabilities of 78% were achieved with region specific correct predictions of 53% to 100%. ^1^H-NMR and isotopic fingerprinting were utilized to investigate the geographical origin or protected designation origin (PDO) of 125 virgin olive oils from five Mediterranean countries, using a variety of supervised or unsupervised statistical tools [186]. Mannina et al. [187] investigated by ^1^H NMR the presence of refined hazelnut oil in admixtures with refines olive oils. Analysis of 92 samples with multiple regression models predicted, with R^2^ >0.9984, contamination of hazelnut oil in olive oil at concentrations as low as 10%.

#### 5.6.2. Authentication and Quality Assessment of Dairy Products

^1^H-NMR was used to characterize the lipid fraction of buffalo and cow milk samples [188]. Multivariate statistical analysis of the quantification results permitted buffalo and cow milk to be differentiated. The total concentration of saturated and unsaturated FAs were eliminated from the data since they were found to be highly correlated with the MUFA concentrations.

**Figure 36:**
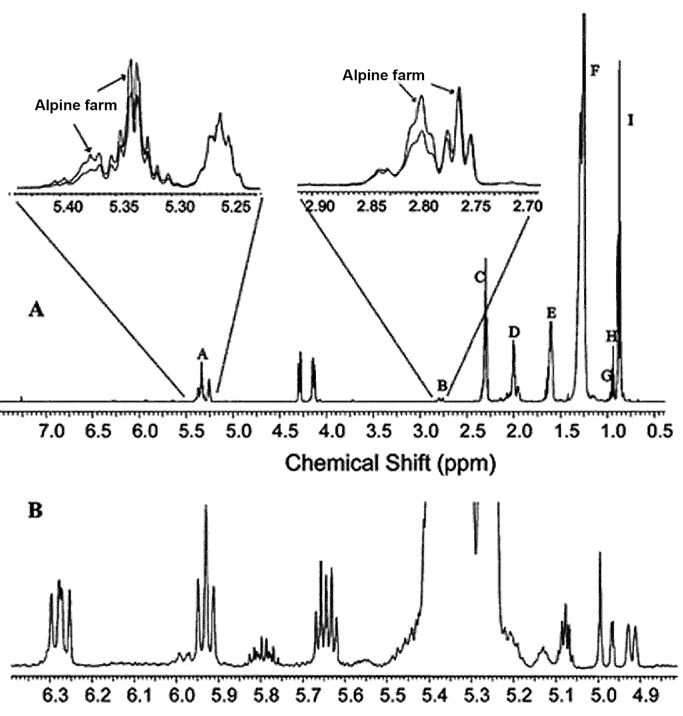
600 MHz ^1^H-NMR spectra of: (**A**) an organic lipid fraction of an Asiago cheese from an alpine farm with the assignment of the major components (A, olefinic protons of all unsaturated chains; B, bis-allylic protons; C, methylenic protons bonded to C2 of all fatty acid chains; D, allylic protons; E, methylenic protons bonded to C3; F, methylenic protons; G, methyl protons of linolenic acid; H, methyl protons of butyric acid; I, all other methyl protons. The insets illustrate a comparison of the lipid fraction of an alpine farm and an industrial factory. (**B**) Expansion of a region in which some important olefinic resonances from CLA are illustrated. Adopted, with permission, from [189]. Copyright 2008, American Chemical Society.

**Figure 37:**
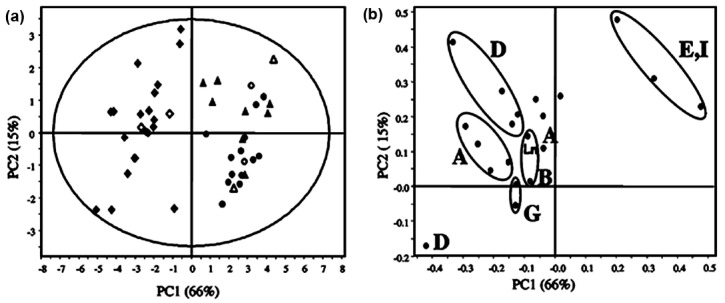
PCA of the ^1^H-NMR resonances of lipid fractions: (**a**) score plot of PC1 vs. PC2 [training set (◆) alpine farms; (•) lowland industrialized factories, (▲) mountain industrialized factories; test set (◊) alpine farms; (○) lowland industrialized factories, (△) mountain industrialized factories]; (**b**) loadings profile (letters correspond to Figure 36). Adopted, with permission, from [189]. Copyright 2008, American Chemical Society.

Schievano et al. [189] utilized a combination of ^1^H-NMR selective 1D TOCSY, ^13^C and ^1^H-^13^C HMQC to discriminate Asiago d’ Allevo cheese samples. The cheese, which is produced from raw dairy milk, has a protected denomination of origin (PDO) mark. Figure 36 illustrates selected regions of the ^1^H-NMR spectrum of a representative cheese sample from an alpine farm and comparison with the spectrum from an industrial factory (insets in Figure 36). A higher amount of unsaturated fatty acids, both in the olefinic and bis-allylic regions, were observed in the cheese from alpine farms. A statistical analysis of the NMR spectra of the lipid fraction demonstrated that the cheese samples from alpine farms are clearly separated from the remaining ones and are clustered in the left side of the PCA plot. The loadings in Figure 37 demonstrate PC1 negative values for the olefinic and allylic protons, the methyl groups of linolenic acid and the bis-allylic protons in which case the higher contribution is from linolenic acid. On the contrary, PC1 loadings show positive values for the saturated fatty acids. It was concluded that cheeses produced in alpine farms are significantly different i.e., they contain higher amount of PUFA, from those produced in low land and mountain industrialized factories based on objective NMR metabolite parameters.

Of particular interest is the application of ^1^H-NMR to whole milk without any pre-treatment [190]. The ^1^H-NMR signals of butyric acid, mono and polyunsaturated fatty chains and lecithin have been assigned using 2D NMR experiments.

Chemometric analysis of ^1^H and ^13^C-NMR, stable isotope-ratio mass spectrometry and GC data was utilized to differentiate organic and conventional milk [191]. Yang et al. [192] investigated metabolic biomarkers of the water soluble fraction of milk from Holstein cows and other minor dairy animals. Data of the lipid fraction, however, were not reported.

#### 5.6.3. Authentication and Quality Assessment of Fish and Meat Lipids

The increasing production and consumption of fish products, has led to an increasing demand for analytical methods for the unequivocal determination of wild vs. farmed specimens, ecological products and geographical origins. The lipid variability and profile of fish is very large with about 20 fatty acids in relative amounts >1%.

**Figure 38:**
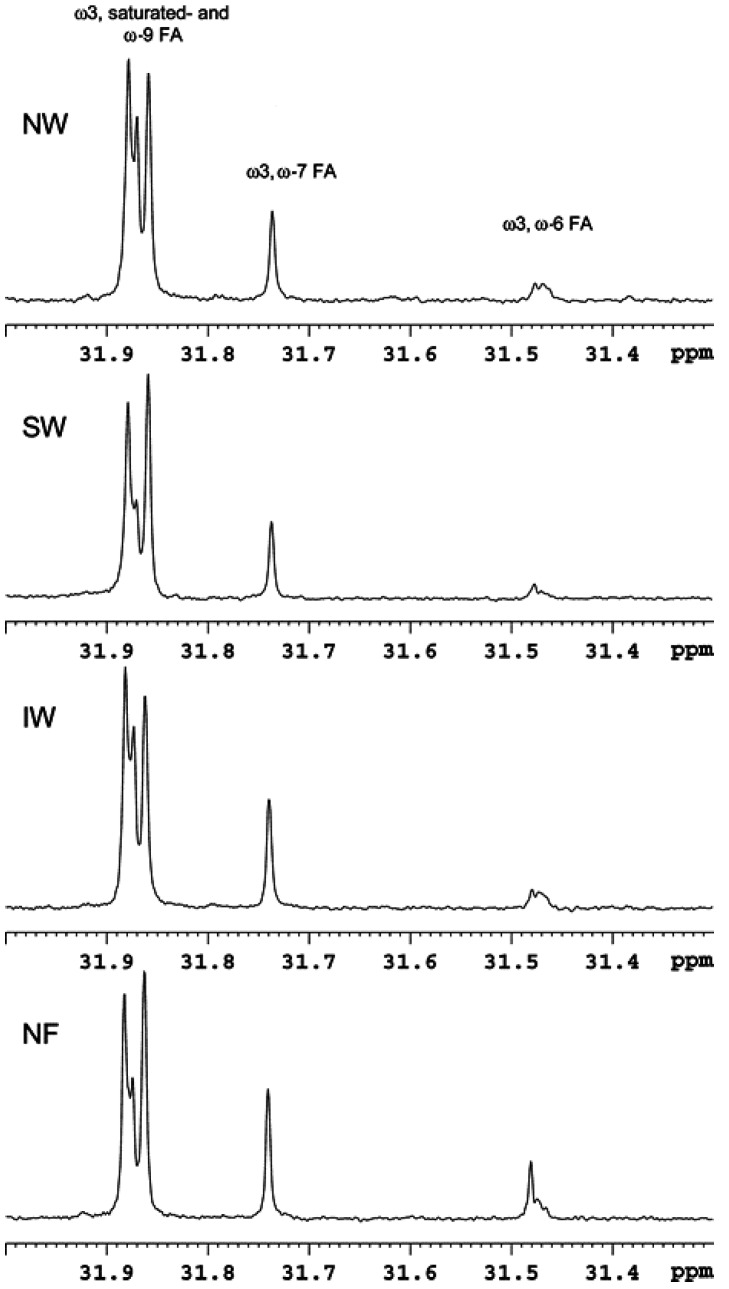
^13^C-NMR aliphatic region (32.0–31.3 ppm) of lipids extracted from salmon of four different origins: (from top) wild salmon from Norway (NW), Scotland (SW), and Ireland (IW) and farmed salmon from Norway (NF). Adopted, with permission, from [193]. Copyright 2009, American Chemical Society.

Aursand et al. [193] utilized ^13^C-NMR and pattern recognition techniques for the discrimination of farmed and wild Atlantic salmon (*Salmo salar* L.), different geographical origins and verification of the origin of market samples. Muscle lipids of 195 samples from seven different countries in addition to market samples, were analyzed. Excellent discrimination (98.5% to 100%) was achieved between wild and farmed salmon. Correct classification with respect to geographical origin was from 82.2% to 99.3%. The farmed fish was found to have a relatively high level of ω-6 fatty acids, abundant in vegetables oils, compared to the wild fish (Figure 38). ^13^C-NMR was used for the regiospecific analysis of TAG in fish oils [194]. Through PCA analysis, it was concluded that the chemical shifts of the *sn*-2 MUFAs and SFAs contribute mostly to the classification of different salmon species. ^13^C-NMR was also applied for the classification of 112 commercial fish oil capsule products using unsupervised multivariate analysis, Kohonen neural networks and generative topographic mapping [195]. ^1^H-NMR profiling of lipids extracts combined with PCA and LDA was used to classify wild and farmed samples of gilthead sea bream from the Mediterranean region [196] and to investigate the effect of various production practices.

**Figure 39:**
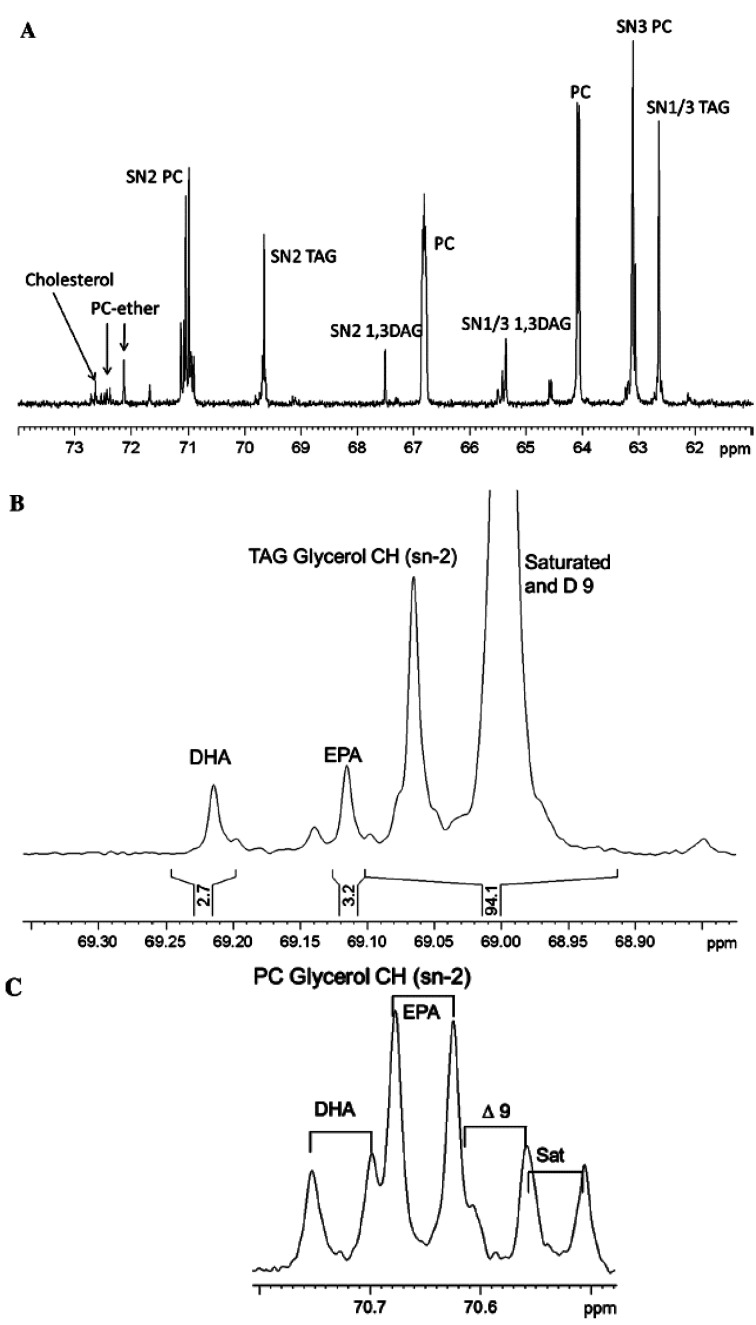
^13^C-NMR spectrum of krill oil in the region of glycerol carbon atoms (**A**). Selected ^13^C regions of triacylglycerol (TAG) (**B**) and phosphatidylchorine (PC) (**C**). Adopted, with permission, from [197]. Copyright 2016, AOCS.

Although the glycerol carbon atoms are usually considered to be less sensitive to individual asymmetric distribution, Diehl et al. [197] were able to investigate detailed regiospecific analysis of the FA distribution in neutral and polar lipids from krill oil. In contrast to fish oil where EPA and DHA are found in the TAG form, krill oil is characterized by lower amounts of TAG. Figure 39 illustrates the dominant presence of PUFAs and more specific of DHA and EPA in the *sn*-2 position of phosphatidylcholine (PC).

^31^P-NMR was validated, with regard to major phospholipid species of krill oil, according to Good Laboratory Practice (GLP) and International Council for Harmonization (ICH) guidelines [197]. It was demonstrated that the method is highly accurate, sensitive, reproducible and robust regardless of the type of NMR spectrometer used by different laboratories.

^1^H-NMR has also been used in meat authentication and determination of geographical origin and investigation of adulteration practice of mixing meat from different species or tissues. Al-Jowder et al. [198] reported that ^1^H-NMR can be utilized to distinguish liver, kidney and muscle tissues of beef. Authentication of beef vs. horse meat was achieved using a very low frequency (60 MHz) instrument [199]. The analysis of 107 extracts was based on the ^1^H-NMR signals of olefinic, bis-allylic, and terminal –CH_3_ groups. ^1^H-NMR fingerprinting of lipids with chemometric tools allowed the detection of irradiated vs. non irradiated beef samples [200]. NMR and isotope ratio mass spectrometry was used to characterize animal products according to geographic origin and feeding diet [201]. ^13^C-NMR demonstrated that the degree of saturation of FAs and MUFAs was significantly higher (*p* < 0.005) for animals fed on maize silage than for grazing steers (vice versa for PUFAs and SFAs).

#### 5.6.4. Serum/Plasma Lipoprotein Analysis

**Figure 40:**
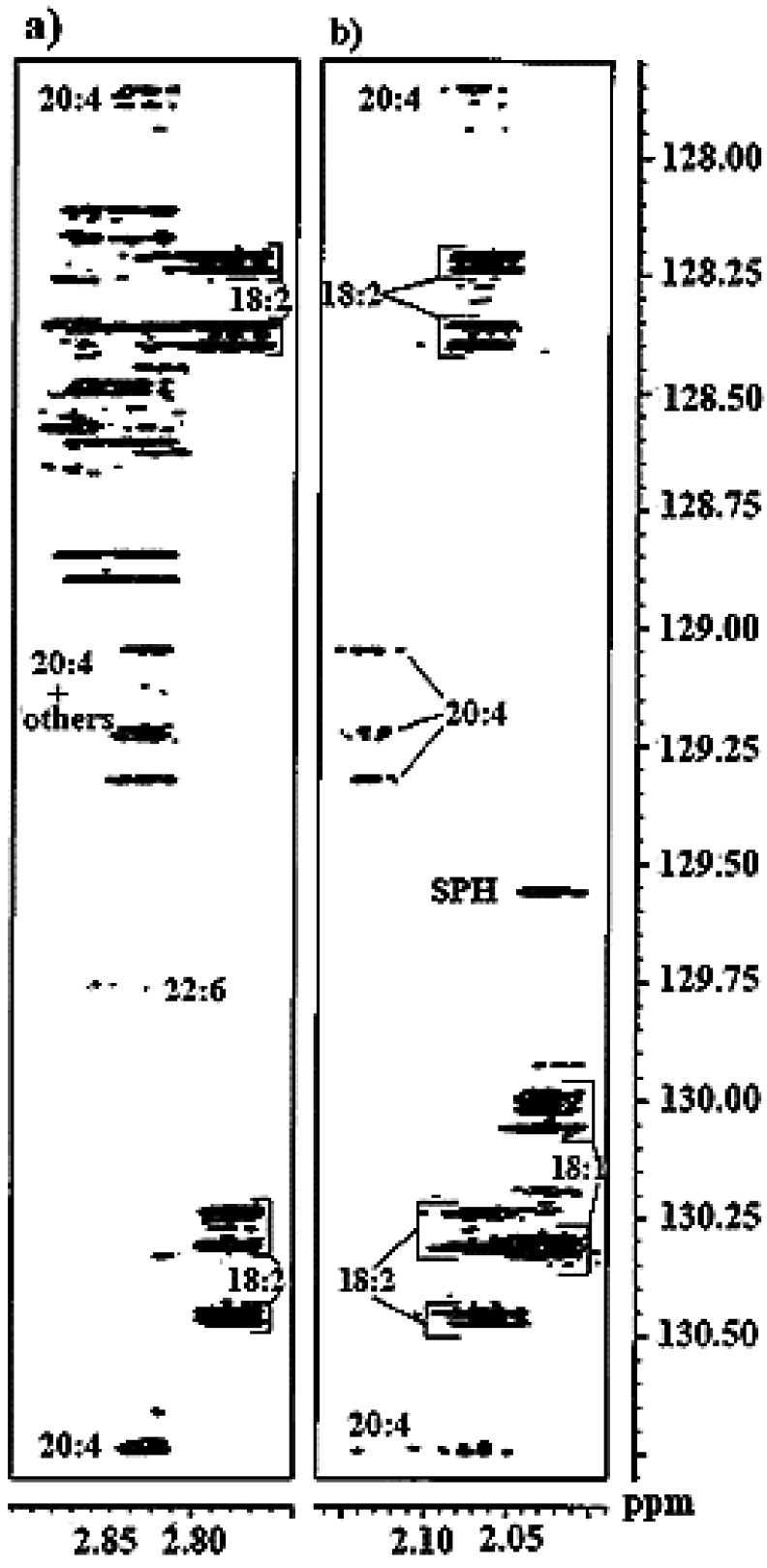
Semi-selective 800 MHz HSQC-TOCSY spectrum of the double bond region of blood plasma lipids. (**a**) Correlations to the allylic Δ–CH_2_–Δ protons (ca. 2.8 ppm) (b); Δ–^1^CH protons (ca*.* 2.05 ppm). In the Δ–CH_2_–Δ region the 18:2 signals are well separated from the higher polyunsaturated fatty acids, whereas in the Δ–1 region the 18:1 signals are separated from all other signals. The 20:4 fatty acid shows additional well separated signals at 2.13 ppm. Adopted, with permission, from [89]. Copyright 1998, John Wiley & Sons, Ltd.

Several human diseases show abnormal patterns of unsaturated fatty acids especially due to insufficient capabilities for desaturation or chain elongation. Therefore, the assignment and quantification of individual unsaturated fatty acids in body fluids are of great importance. Willker and Leibfritz utilized a semi-selective ^1^H-^13^C HSQC experiment [86,89] to obtain ultra-high resolution in the ^13^C double bond region (~0.7 Hz per point) and, thus, to assign and quantify individual unsaturated fatty acids, such as 18:1, 18:2 and 20:4 in body fluids (blood plasma and cerebrospinal fluid) and pig brain (grey and white matter). Additional ^1^H-^13^C HSQC-TOCSY experiments allowed the direct correlation between the olefinic ^13^C signals with the adjacent allylic ^1^H signals. Figure 40 illustrates selective 800 MHz ^1^H-^13^C HSQC-TOCSY spectrum of the double bond region of blood plasma lipids. In the C=C–CH_2_–C=C region the 18:2 signals are well separated from 18:3 and 20:4 polyunsaturated fatty acids. In the C=C–CH_2_– region the 18:1 and 20:4 signals are well separated from the rest of the lipids [89].

**Figure 41:**
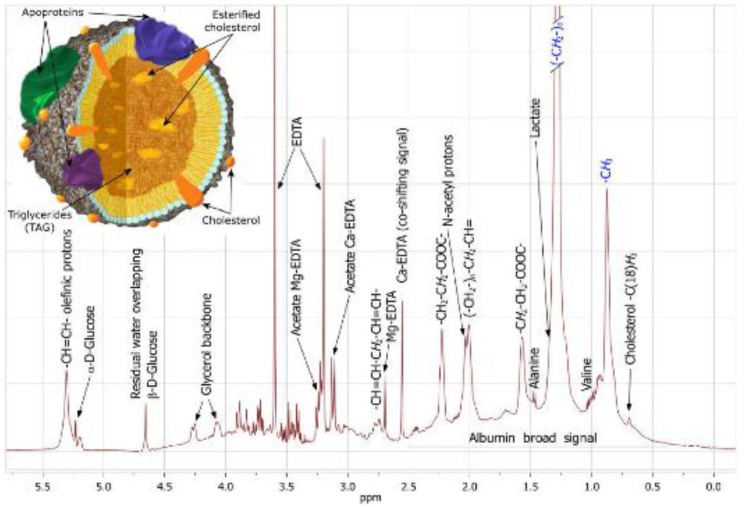
Representative ^1^H-NMR spectrum (500 MHz) of human blood. Inset shows a schematic view of the micellar chylomicron structure. Adopted, with permission, from [202]. Copyright 2010, BioMed Central Ltd.

NMR spectroscopy has been extensively utilized for the qualitative and quantitative determination of lipoproteins, cholesterol and triacylglycerides (Figure 41 [202]) and the number of sub fraction articles and their size [203,204]. Numerous studies have been reported to monitor changes in the organization of lipids within particle subclasses [205], to investigate the effects of exercise on lipoprotein profile [206], changes in lipids induced by diet therapies [207], lipoprotein analysis in the study of diabetes and related diseases [208], in assessing CVD risk [202] and in investigating the progression of coronary heart disease [209]. Readers should consult a recent comprehensive review article [203].

A diffusion-edited NMR spectrum enhances the NMR signal of lipoproteins and removes signals from small metabolites [210]. This allows the composition of the constituent lipids of lipoproteins to be assigned and analyzed, through the acquisition of a set of diffusion-edited NMR spectra with an increasing strength in gradient, which enables the translational diffusion coefficients to be calculated.

For the quantification of cholesterol and triacylglycerides in several sub fractions and the calculation of particle number, the decomposition of the CH_3_ groups and/or correlation statistical methods have been utilized [211,212,213]. Peak overlapping, however, is very severe which makes the lineshape fitting to individual sub fractions problematic and prone to finding multiple solutions [204].

### 5.7. Investigation of Lipid-Derived Molecules

Lipid-derived molecules play a pivotal role in many biological processes including cellular signaling, secretion and cellular proliferation [214]. Brash et al. [215] isolated and characterized two geometric allene isomers synthesized from natural 9*S*-hydroperoxylinoleic acid by allene oxide synthese cytochrome P450 CYB74C3. The obtained results, especially of a novel 10 *cis* isomer, provided insights into the mechanisms of allene oxide cyclization and the double bond geometry in naturally occurring allene oxides.

Liu et al. [216] characterized by GC-MS and ^1^H-NMR several dihydroxylated metabolites derived from α-linolenic acid (9(*R*),16(*S*)-dihydroxy-10*E*,12*E*,14*E*-octadecatrienoic acid, 9(*S*),16(*S*)-dihydroxy-10*E*,12*E*,14*E*-octadecatrienoic acid, 9(*S*),16(*S*)-dihydroxy-10*E*,12*Z*,14*E*-octadecatrienoic acid, and 9(*R*),16(*S*)-dihydroxy-10*E*,12*Z*,14*E*-octadecatrienoic acid) treated by soybean 15-lipoxygenase (sLOX). Despite the use of a 1 GHz (23.4 T) NMR instrument several selective decoupling, 2D DQF COSY, and 2D-J resolved experiments were necessary for unequivocal assignments of the resulting dihydroxylated metabolites. Mono- and dihydroxyresolvins from EPA and DHA using soybean 15-lipoxygenase were also investigated [217]. Of particular interest is the use of D-camphor ketals as ^1^H-NMR shift reagent to investigate the unusual stereochemical configuration of an endosome-specific lipid [218].

### 5.8. Protein-Lipid Interactions

Large amounts of free fatty acids, FFA, circulate in mammalian plasma and play significant role in a variety of physiological activities, as substrates for complex protein-lipid interactions and in important signaling events [219,220]. FFA are present in blood at concentration from about 100 μM to more than 0.1 mM. The pool of circulating FFA is composed of approximately forty distinct molecular species. The FFA profile is expected to reflect the physiological state and changes in the profile have been correlated with diseases [221].

**Figure 42:**
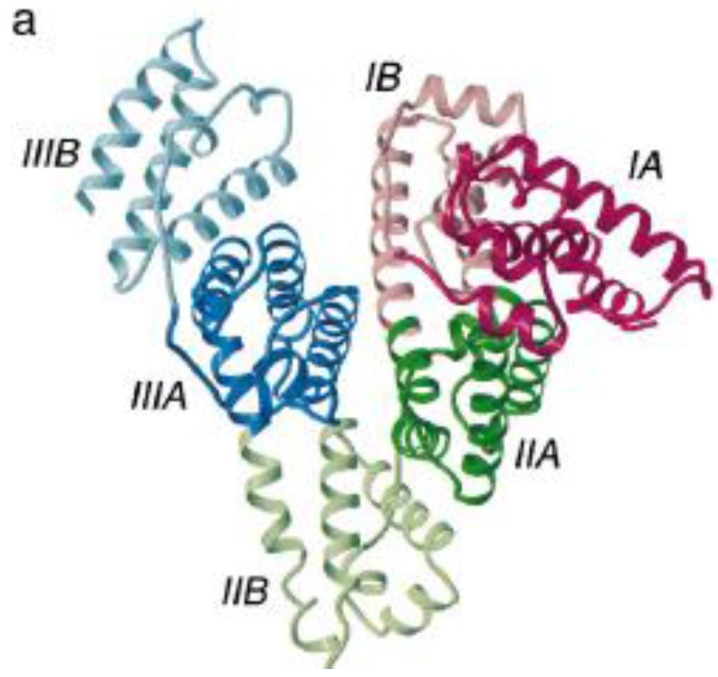

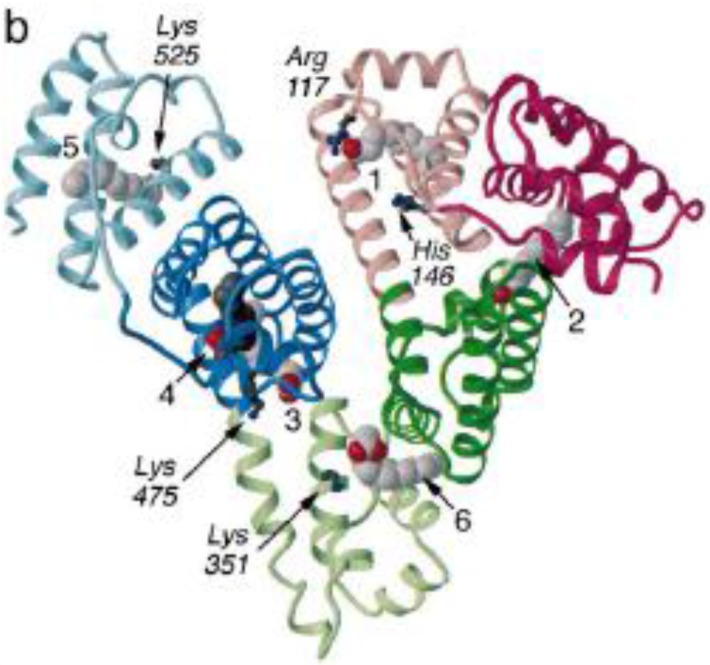
Binding of fatty acid to HSA induces significant conformational changes in the protein. (**a**) Crystal structure of unliganded HSA [223] (PDB ID, 1ao6). The subdomains are color-coded as follows: IA, red; IB, light-red; IIA, green; IIB, light-green; IIIA, blue; IIIB, light-blue. (**b**) Crystal structure of HSA-myristate [224] (PDB ID, 1bj5). Both crystal structures have been fully refined to 2.5 Å resolution. The six myristate molecules are numbered 1–6 and shown in a space-filling representation. Adopted, with permission, from [225]. Copyright 1999, Elsevier Science B.V.

Many FFA are bound to serum albumin. Under normal physiological conditions 0.1–2 moles of fatty acids are bound to albumin. The fatty acid ligands are in rapid exchange between solution and their binding sites in the protein. Human serum albumin (HSA) is a monomeric protein of 585 amino acids with a molecular weight of 66.4 kDa. The X-ray structure determination [222,223] demonstrated that the protein is 67% α-helical, without β-sheets, with three homologous domains (labeled I–III) and 17 intra-subdomain disulphide bridges which are conserved across species and contribute to the high thermostability of the protein. Each domain is comprised of two subdomains. The X-ray structure of the complex of HSA with myristate revealed six fatty acid binding sites [224]. Five of the sites (numbers 1–5 in Figure 42) are characterized by a strong electron density of the ligand [225]. The sixth ligand was tentatively assigned at the interface between the subdomains IIA and IIB. The binding sites 1, 4 and 5 are within a single subdomain in IB, IIIA and IIIB, respectively. The binding sites 2 and 3 incorporate residues from different domains [225].

A number of biochemical labeling and NMR experiments [226,227,228] implicated domains I and III in fatty acid binding. In particular, NMR studies using fatty acids selectively labeled with ^13^C demonstrated that the carboxylate group of the fatty acid ligand was bound more tightly in the binding site due to electrostatic interactions, than the flexible hydrophobic methylene chain [229,230]. Subsequent NMR studies with ^13^C-labeled laurate, myristate, palmitate, stearate and oleate were interpreted with the existence of at least five distinct chemical shifts for the carboxylate carbon due to five distinct binding sites with BSA [231,232,233]. Variation of the pH revealed that four of the five binding sites are involved in electrostatic interactions in the pH range of 4-8 due to ionic hydrogen bonds between the carboxylate group of the fatty acid and side chain of basic aminoacids, such as arginine or lycine. It should be noticed that the X-ray structure demonstrates ionic hydrogen bond interactions for all six binding sites. Comparative NMR studies of intact and proteolitic fragments of BSA identified three high-affinity sites of oleic acid [226], one in domain I and two in domain II.

Detailed NMR studies of the binding of ^13^C-carboxyl labelled palmitate to HSA in the presence of competitor ligands and drugs, for specific binding sites, allowed the location of high and low affinity fatty acid and the correlation of specific binding site from the X-ray structures to specific ^13^C-NMR signals.

**Figure 43:**
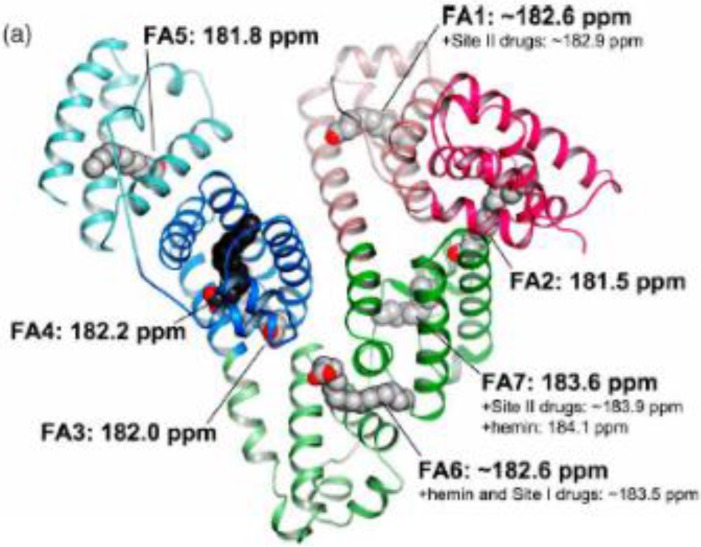

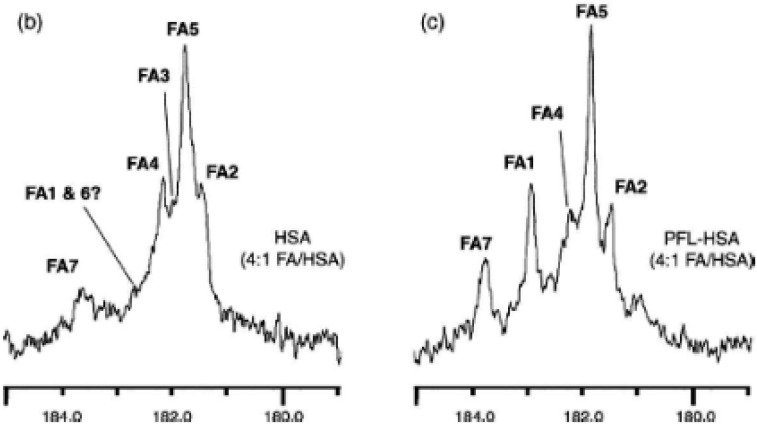
Correlation of the ^13^C-NMR and crystal structure data for HSA. (**a**) Crystal structure of HSA complexed with palmitate. Labels indicate the FA binding sites and their assigned chemical shifts. The effects of various drugs on these chemical shifts are noted. (**b**) Spectrum of [^13^C] palmitate bound to HSA at a FA/HSA mole ratio of 4:1. Although a broad shoulder signal may be present at ∼182.6 ppm, the peaks for FA sites 1 and 6 are typically not observed in spectra of wild-type HSA at the FA/HSA ratios investigated. The positions of these two peaks (derived from multiple competition experiments) are indicated in the spectrum for [^13^C]-palmitate bound to wild-type HSA at a ratio of 4:1. (**c**) Spectrum of [^13^C] palmitate bound to PFL-HSA at a FA/HSA mole ratio of 4:1. Adopted, with permission, from [236]. Copyright 2013, Elsevier B.V.

**Figure 44:**
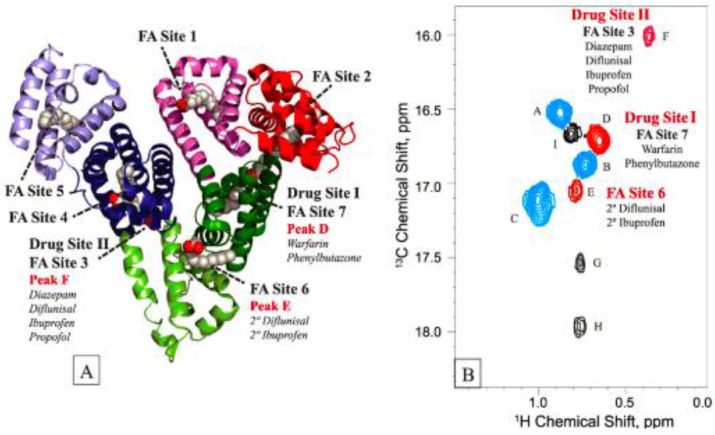
Summary of oleic acid (OA)-HSA competition experiments. (**A**) Crystal of HSA bound to seven labeled OA molecules. Drugs and endogenous compound primary and secondary binding sites are listed (PDB entry 1E7G). (**B**) ^1^H-^13^C HSQC spectrum of the 4:1 OA–HSA complex. FA-2, -4, and -5 (blue) are primary sites for FA and are generally not impacted by competition binding. The low-affinity sites, FA-3, -6, and -7 (red), were identified through drug competition experiments. The locations of the competition of the drugs and endogenous compounds for their primary and secondary binding are listed. The spectrum was recorded at 500 MHz, 25 °C, and pH 7.4 in a 50 mM phosphate, 50 mM NaCl buffer. Adopted, with permission, from [235]. Copyright 2013, by the American Chemical Society.

Figure 43 illustrates a strategy of resolving and identifying peaks by drug competition. FA site 1 appears as a well-resolved peak and FA site 2 with enhanced resolution. The propofol (PFL), site II drug, displaces FA from sites 3 and 4 and, thus, 4 attenuates the NMR peaks corresponding to those sites. Displacement of FA from these sites leads to enhancement of the signal from site 1 [234]. It was demonstrated that fatty acid sites 2,4 and 5 bind FA with high affinity, while sites 1, 3, 6 and 7 exhibit low affinity to FA. More recently, the enhanced sensitivity and resolution of ^1^H-^13^C HSQC experiments using [^13^C]-methyl-labeled oleic acid 18:1 provided evidence of nine distinct binding sites to HSA (Figure 44) [235]. Individual binding sites were detected in the NMR spectra since the binding sites are structurally distinct and the binding site exchange rates are slow in the NMR time scale. Detailed analysis of competition experiments, with a wide range of drugs, provided evidence for site-specific characterization of the mutual effects of FA and ligand binding [235,236].

NMR studies have not been published using isotopic labeling of specific protein aminoacids with ^13^C and ^15^N to investigate the effect of pH on fatty acid binding. Furthermore, to the best of our knowledge, saturation transfer difference (STD) [237,238,239] and Tr-NOESY [240,241] experiments for unsaturated lipids have not been applied. These experiments would allow the rapid characterization of the binding epitope of the ligand closest to the receptor and the conformational changes upon ligand binding without the need of selectively enriched lipids.

## 6. Practical Considerations

### 6.1. Preparation of Solid and Liquid Samples

The sample preparation includes sampling, concentration and homogenation. In the case of dairy products specific methods of conservation should be used in order to preserve the quality and the composition of nutrients. For animal tissues, rapid metabolic changes can be quenched by a rapid freezing in liquid nitrogen after slaughter [24]. Lyophlization is a common method for long-term stability of samples which increases the efficiency of extraction of lipids due to disruption of cellular membranes [242,243] and increased surface area, and provides a convenient method for a normalization of metabolites per sample dry weight [24]. Some volatile analytes may be lost during the freeze-drying process although, compared to other drying methods, the loss of these compounds may not be a major concern [244]. In the case of oils, the analysis does not involve any pretreatment although, due to high viscosity, dilution is necessary as a preliminary step [114]. The significant effect of concentration on the chemical shifts, should also be taken into account (see discussion below).

### 6.2. Lipid Extraction Methods

**Figure 45:**
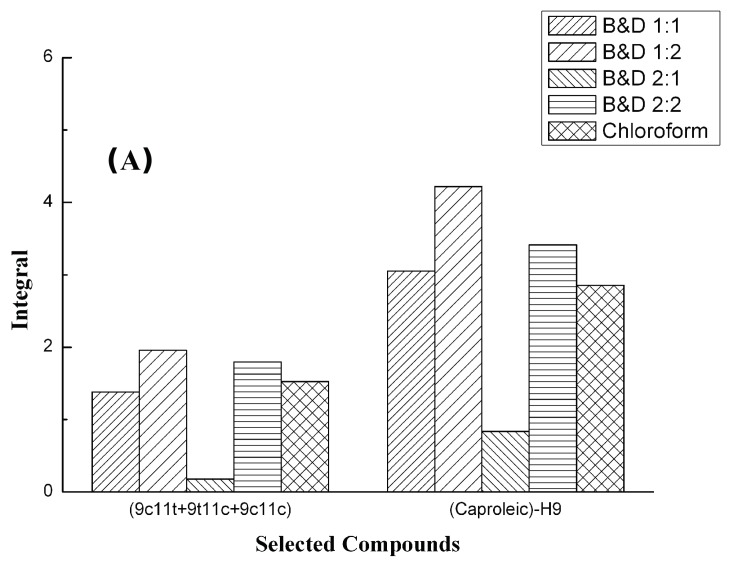

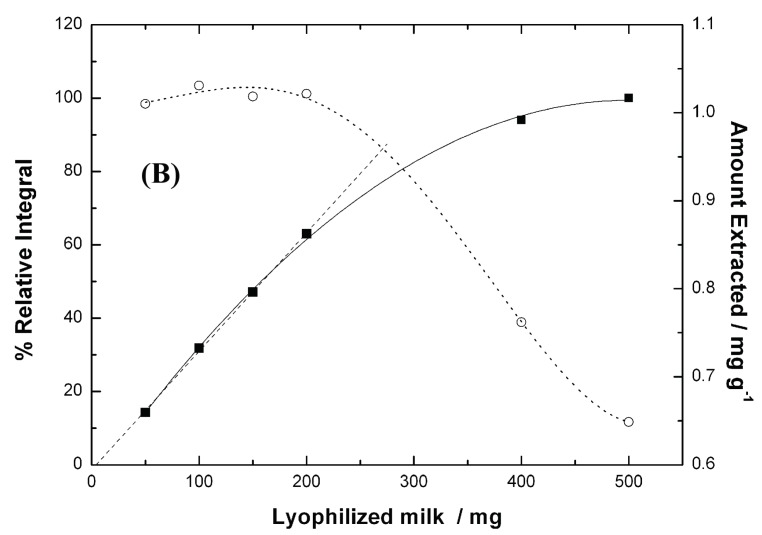
(**A**) Comparison of various extraction methods in respect to the total (9-*cis*, 11-*trans*), (9-*trans*, 11-*cis*) and (9-*cis*, 11-*cis*) 18:2 CLA isomers and caproleic acid integrals. (**B**) Efficiency of the extraction of the milk lipid fraction using the Bligh and Dyer 1:2 method in respect to mg of lyophilized milk sample; (■), the relative integrals of the composite (9-*cis*, 11-*trans*), (9-*trans*, 11-*cis*) and (9-*cis*, 11-*cis*) 18:2 CLA isomers in respect to the standard reference compound as above. (○), the amount of the extracted composite (9-*cis*, 11-*trans*), (9-*trans*, 11-*cis*) and (9-*cis*, 11-*cis*) 18:2 CLA isomers expressed in mg g^−1^ of lyophilized milk sample. Adopted, with permission, from [56]. Copyright 2014, by the Elsevier B.V.

Sample preparation is one of the most important steps in the analysis of lipids since it can affect analyte concentration and, thus, the accuracy of quantitative results. The Folch et al. [245] and Bligh-Dyer [246] methods and their variants have been widely used. The method relies upon partitioning the lipid matrix into two immiscible layers, as upper water-methanol and a chloroform one. Optimization of the extraction process can involve several parameters such as agiation or sonication assisted, time duration and temperature of the extraction, the lipid content extracted and the optimum chloroform volume [56]. Figure 45 illustrates a comparison of the efficiency of various extraction methods with respect to unsaturated fatty acids in lyophilized milk samples. The concentration of the extracted analytes deviates strongly from linearity for lyophilized milk above 250 mg, presumably due to progressive saturation of the solution. The extracted solution, after a centrifiguration step, can be evaporated or dried under vacuum and subsequently dissolved in deuterated solvent.

### 6.3. NMR Sample Preparation

#### 6.3.1. NMR Solvent, Referenicng and Quantification Methods

Deuterated chloroform has been widely used in ^1^H-and ^13^C NMR since it is an excellent solvent for most lipids. For specific applications, mixtures of CDCl_3_ and DMSO-*d*_6_ have also been utilized [132,164]. DMSO-*d*_6_ induces significant chemical shift changes with respect to those in CDCl_3_ which should be taken into consideration when comparing literature data. The chemical shift of the residual ^1^H-NMR signal of CDCl_3_ and the ^13^C signal of CDCl_3_ have been commonly utilized for referencing.

Quantification of unsaturated fatty acids can be achieved in ^1^H-NMR using Equations (1) to (10) without the use of internal standard. As previously emphasized, the conversion of ^1^H-NMR data from mol % to wt % can be achieved with the hypothesis that the average chain lengths of unsaturated fatty acids are C_18_ and by estimating the average number of protons in unsaturated and saturated chains from the integrals of given resonances in the spectrum [124]. An alternative method for quantification is the ERETIC method which provides a reference signal synthesized by an electronic device [247]. Quantification in ^13^C-NMR can be obtained by integration of the appropriate carbonyl and olefinic signals [20,92].

For ^31^P-NMR, pyridine-chloroform solutions have been utilized (1.6:1.0 volume ratio) containing 0.165 μM of Cr(acac)_3_. Integration of the signal was obtained with respect to cyclohexanol as an internal standard [23,59].

#### 6.3.2. Effects of Temperature and Conentration 

Most ^1^H-, ^13^C- and ^31^P-NMR spectra have been acquired in the range of 296 to 298 K, therefore, no significant changes in the chemical shifts of literature data due to temperature variation are expected.

The effect of concentration on chemical shifts is an important aspect which should be considered in lipid analysis. The use of high concentrated solutions in ^1^H NMR results: (i) in broadening of the NMR signals and, thus, poor resolution, (ii) baseline distortions and spurious signals due to receiver overload, (iii) saturation of analytes and, thus, unreliable quantification results and (iv) chemical shift variation. Vlahov et al. [92] investigated in detail the effect of sample concentration (200 mg and 50 mg) on the ^13^C NMR chemical shifts of lampante olive oils. In most cases, chemical shift variations below 0.2 ppm were observed, however, the large chemical shift variations of the methylene C-2 and C-3 carbons prevented an unequivocal assignment.

## 7. Conclusions and Future Prospects

The research work summarized in this article provides sufficient evidence that ^1^H-, ^13^C- and ^31^P-NMR spectroscopy has been a valuable structural and analytical tool for unsaturated lipids The quantitative analysis performed by integration of the appropriate signals is greatly facilitated by the unequivocal assignment of the signals using a variety of 1D and 2D NMR techniques, such as ^1^H-^1^H TOCSY, ^1^H-^13^C HSQC, ^1^H-^13^C HMBC, ^1^H-^13^C HSQC-DEPT, etc. Further, the latest developments with respect to sensitivity, and the introduction of cryogenerated micro-NMR probes have greatly facilitated the rapid de novo identification of specific polyunsaturated fatty acids.

NMR spectroscopy will undoubtedly continue to be developed as a primary structural and analytical spectroscopic method in the field of unsaturated lipid research. It should be emphasized, however, that it appears unlikely that any single NMR technique will be a panacea for the complete structural analysis of lipids. Cited below are selected current and potential areas where further research could be fruitful:

(1) The excellent resolution and sensitivity advantages of the selective 1D TOCSY and band selective ^1^H-^13^C HSQC, ^1^H-^13^C HSQC-TOCSY and ^1^H-^13^C HMBC experiments show great potential in deciphering complex lipid extracts and oxidation products, even though such methods are still not frequently used in lipid research.
(2) Application of broadband ^1^H homonuclear decoupled techniques will result in highly resolved ^1^H-NMR spectra with collapsed singlets, thus minimizing overlap and expediting spectral analysis [248,249]. Several novel 1D and 2D selective experiments and improved slice—selective experiments have been proposed which can provide ultra-highly resolved NMR spectra with great potentialities for accurate determination of very small chemical shift differences, coupling constants, and relaxation times [250,251,252,253,254].
(3) Development of comprehensive NMR databases combined with prediction software will greatly improve the amount of structural information that can be extracted from ^1^H, ^13^C, ^1^H-^1^H COSY, ^1^H-^1^H TOCSY, ^1^H-^13^C HSQC, and ^1^H-^13^C HMBC NMR data and would accelerate the dereplication process [255,256].
(4) Application of hyperpolarizable techniques will greatly improve sensitivity since they are capable of generating spin population levels that are ~5 × 10^4^ times higher than the Boltzman equilibrium at room temperature [257]. Particularly promising results can be obtained in solution state by dissolution dynamic nuclear polarization (DNP), where the sample to be analyzed is mixed with free radicals in a solution frozen to liquid helium temperatures and hyperpolarized by irradiating in the vicinity of the ESR of the unpaired electrons of the radicals [258,259].
(5) Development of automated hyphenated LC-SPE-NMR-MS platforms [260].
(6) More comprehensive and systematic comparisons between NMR and other analytical methods, such as GC-MS, should be performed in order to investigate their limitations, strengths and weaknesses as well as the advantages of their combined use.
(7) Development of officially recognized NMR methodologies for fats and oils through collaboration of various NMR groups.
(8) Application of STD and Tr-NOESY techniques would allow the analysis of interactions of chemically unmodified fatty acids with full-length proteins and, thus, provide further understanding of fatty acid conformational changes upon binding to lipoproteins even at a cellular level [240,241].
(9) Further developments in DOSY (Diffusion Order Spectroscopy) can make it special powerful tool for the analysis of unsaturated fatty acids and lipids [248].
(10) DFT calculations of ^1^H-, ^13^C- and ^13^P-NMR chemical shifts [261,262,263,264,265] can contribute significantly in the new field of NMR “crystallography” in the powder [266,267] and in solution [268,269].

It is hoped that this review will provide some guidance and stimulus for future NMR applications in the field of unsaturated lipid research.

## Figures and Tables

**Table 1:** Some representative unsaturated and polyunsaturated ω-6 fatty acids.

Common Name	Lipid Notation	Δ^n^ Notation	Molecular Formula
Oleic acid	18:1 (ω-9)	Δ^9^	CH_3_(CH_2_)_7_CH=CH(CH_2_)_7_COOH
Linoleic acid	18:2 (ω-6)	Δ^9,12^	CH_3_(CH_2_)_4_CH=CHCH_2_CH=CH(CH_2_)_7_COOH
Arachidonic acid (AA)	20:4 (ω-6)	Δ^5,8,11,14^	CH_3_(CH_2_)_3_(CH_2_CH=CH)_4_(CH_2_)_3_COOH
γ-Linolenic acid	18:3 (ω-6)	Δ^6,9,12^	CH_3_(CH_2_)_3_(CH_2_CH=CH)_3_(CH_2_)COOH
Dihomo-γ-linolenic acid	20:3 (ω-6)	Δ^8,11,14^	CH_3_(CH_2_)_3_(CH_2_CH=CH)_4_(CH_2_)_3_COOH
Adrenic acid	22:4 (ω-6)	Δ^7,10,13,16^	CH_3_(CH_2_)_3_(CH_2_CH=CH)_4_(CH_2_)_5_COOH

**Table 2:** Common ω-3 fatty acids found in Nature.

Common Name	Lipid Notation	Chemical Name
n/a	16:3 (ω-3)	all-*cis*-7,10,13-hexadecatrienoic acid
α-Linolenic acid (ALA)	18:3 (ω-3)	all-*cis*-9,12,15-octadecatrienoic acid
Stearidonic acid (SDA)	18:4 (ω-3)	all-*cis*-6,9,12,15-octadecatetraenoic acid
Eicosatrienoic acid (ETE)	20:3 (ω-3)	all-*cis*-11,14,17-eicosatrienoic acid
Eicosatetraenoic acid (ETA)	20:4 (ω-3)	all-*cis*-8,11,14,17-eicosatetraenoic acid
Eicosapentaenoic acid (EPA)	20:5 (ω-3)	all-*cis*-5,8,11,14,17-eicosapentaenoic acid
Docosapentaenoic acid (DPA)	22:5 (ω-3)	all-*cis*-7,10,13,16,19-docosapentaenoic acid
Docosahexaenoic acid (DHA)	22:6 (ω-3)	all-*cis*-4,7,10,13,16,19-docosahexaenoic acid
Tetracosapentaenoic acid	24:5 (ω-3)	all-*cis*-9,12,15,18,21-docosahexaenoic acid
Tetracosahexaenoic acid (Nisinic acid)	24:6 (ω-3)	all-*cis*-6,9,12,15,18,21-tetracosenoic acid

**Table 3:** ^1^H-NMR chemical shifts of alkenyl protons of some methyl octadecenoates in CCl_4_. Reprinted, with permission, from [54]. Copyright 1975, North-Holland Publishing Company.

Double Bond	δ-Value	Double Bond	δ-Value
2-*cis*	6.145 5.680	2-*trans*	6.85 5.72
3-*cis*	5.51	3-*trans*	5.47
4-*cis*	5.31	4-*trans*	5.40
5-*cis*	5.32	5-*trans*	5.34
6-*cis*	5.29	6-*trans*	-
7-*cis*	5.28	7-*trans*	-
17-*cis*	5.72 4.94 4.88	17-*trans*	- - -

**Table 4:** ^1^H-NMR resonance assignments of olefinic protons of conjugated linoleic acids (CLAs) in CDCl_3_ [55,56].

Compound	Atom	Functional Group	δ (ppm)	Multiplicity (Hz)
(10-*cis*, 12-*cis*)-CLA	H11, H12	–CH=	6.85	m
(7-*trans*, 9-*cis*)-CLA	H8	–CH=	6.3	dd
(9-*trans*, 11-*cis*)-CLA	H10	–CH=	6.29	t
(9-*cis*, 11-*trans*)-CLA	H11	–CH=	6.28	dd
(9-*cis*, 11-*cis*)-CLA	H10, H11	–CH=	6.22	dd
(10-*trans*, 12-*cis*)-CLA	H11	–CH=	6.22	m
(10-*cis*, 12-*cis*)-CLA	H10, H13	–CH=	6.13	m
(10-*trans*, 12-*trans*)-CLA	H11, H12	–CH=	5.99	m
(9-*trans*, 11-*trans*)-CLA	H10, H11	–CH=	5.96	m
(9-*cis*, 11-*trans*)-CLA	H10	–CH=	5.93	t
(9-*trans*, 11-*cis*)-CLA	H9	–CH=	5.93	m
(7-*trans*, 9-*cis*)-CLA	H9	–CH=	5.93	dd
(10-*trans*, 12-*cis*)-CLA	H12	–CH=	5.87	t
(9-*trans*, 11-*cis*)-CLA	H11	–CH=	5.66	t
(9-*cis*, 11-*trans*)-CLA	H12	–CH=	5.65	m
(7-*trans*, 9-*cis*)-CLA	H7	–CH=	5.64	m
(10-*trans*, 12-*cis*)-CLA	H10	–CH=	5.58	m
(10-*trans*, 12-*trans*)-CLA	H10, H13	–CH=	5.56	m
(9-*trans*, 11-*trans*)-CLA	H9, H12	–CH=	5.54	m
(9-*cis*, 11-*cis*)-CLA	H9, H12	–CH=	5.40	m
(9-*cis*, 11-*trans*)-CLA	H9	–CH=	5.33	m
(7-*trans*, 9-*cis*)-CLA	H10	–CH=	5.31	m
(9-*trans*, 11-*cis*)-CLA	H12	–CH=	5.30	m
(10-*trans*, 12-*cis*)-CLA	H13	–CH=	5.23	m

**Table 5:** ^1^H-NMR chemical shift values (ppm) and coupling constants (Hz) of olefinic hydrogens of conjugated linolenic acids (CL_n_As) in CDCl_3_. Reprinted, with permission, from [58]. Copyright 2006, American Chemical Society.

Proton Notation	β-eleostearic Acid: (9-*trans*, 11-*trans*, 13-*trans*) 18:3	Punicic Acid: (9-*cis*, 11-*trans*, 13-*cis*) 18:3	α-eleostearic Acid: (9-*cis*, 11-*trans*, 13-*trans*) 18:3
9,14-H	5.66, *J* = 12.8 Hz	5.46, *J* = 10.8 Hz	5.40 (9-H, *J* = 10.8 Hz) 5.74 (14-H, *J* = 14 Hz)
10,13-H	6.04, *J* = 12.8 Hz	6.08, *J* = 10.8 Hz	6.01 (10-H, *J* = 10.8 Hz) 6.12 (13-H, *J* = 14 Hz)
11,12-H	6.10, *J* = 11.6 Hz	6.48, *J* = 12.8 Hz	6.40 (12-H, *J* = 13.6 Hz) 6.19 (11-H, *J* = 13.6 Hz)

**Table 6:** ^1^H-NMR resonance assignments of the glycerol moiety of lipids in CDCl_3_ [59,60].

Compound	Functional Group	δ (ppm)	Multiplicity	*J* Coupling (Hz)
Glycerol in TAG	2′-CHOCO–	5.26	m	*J*_1′a,2′_ = 5.9 Hz
*sn*-1,2/2,3 DAG	2′-CHOCO–	5.08	m	-
*sn*-1,2 DAG	1′b-CH_2_–OCO–	4.31	dd	*J*_1′a,1′b_ = 11.9 Hz *J*_1′a,2′_ = 4.5 Hz
Glycerol in TAG	1′a,b-CH_2_–OCO–	4.30	dd	*J*_3′a,3′b_ = 11.9 Hz *J*_3′a,2′_ = 4.4 Hz
*sn*-1,2 DAG	1′a-CH_2_–OCO–	4.23	dd	*J*_1′a,1′b_ = 11.9 Hz *J*_3′a,2′_ = 5.7 Hz
*sn*-1,3 DAG	1′b, 3′b-CH_2_–OCO–	4.18	dd	*J*_1′a,1′b_ = 11.4 Hz *J*_1′a,2′_ = 4.4 Hz
Glycerol in TAG	–CH_2_–OCO–	4.15	dd	-
*sn*-1,3 DAG	1′a, 3′a-CH_2_–OCO–	4.13	dd	*J*_1′a,1′b_ = 11.4 Hz *J*_1′a,2′_ = 6.0 Hz
*sn*-1,3 DAG	–CH_2_–OCO–	4.03	m	-
*sn*-1,2/2,3 DAG	HO–CH_2_–CH–	3.72	m	-
Glycerol in 1-MAG	3′a-CH_2_–OCO-	3.59	dd	*J*_3′a,3′b_ = 11.4 Hz *J*_3′a,2′_ = 6.0 Hz

**Table 7:** ^1^H-NMR features of some characteristic groups in polyenoic fatty acids and esters (in CC1_4_). Reprinted, with permission, from [54]. Copyright 1975, North-Holland Publishing Company.

Group	δ-Value
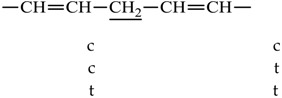 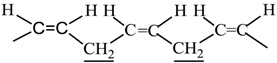	2.72 2.67 2.61 2.76
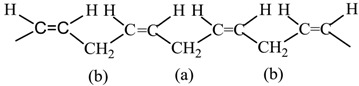	(a) 2.78 (b) 2.76
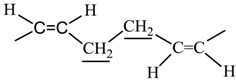	2.04
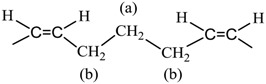	(a) 1.38 (b) 2.00
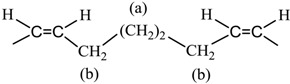	(a) 1.34 (b) 2.00

**Table 8:** ^1^H-NMR chemical shifts of some characteristic groups in polyynoic fatty acids and esters (in CC1_4_). Reprinted, with permission, from [54]. Copyright 1975, North-Holland Publishing Company.

Group	δ-Value
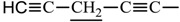	3.06–3.07
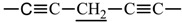	2.98–2.99
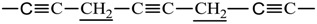	3.04–3.05
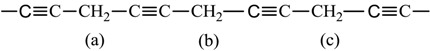	(a) 3.04 (b) 3.0–3.10
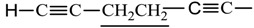	2.33

**Table 9:** ^1^H-NMR resonance assignments of allylic protons, α-protons and CH_3_ groups in CDCl_3_.

Compound	Functional Group	δ (ppm)	Multiplicity
DHA	–OOC–CH_2_–CH_2_–	2.46	d
All FA	–OOC–CH_2_–CH_2_–	2.33	t
ω-3	CH_3_–CH_2_–CH=CH–	2.07	m
ω-6	–CH_2_–CH=CH–	2.03	m
UFA	–CH_2_–CH=CH–	2.02	m
ω-9	–CH_2_–CH=CH–	2.01	m
α-linolenic acid	–CH_3_	0.98	t (7.5 Hz)
Butyric acid	–CH_3_	0.94	t (7.4 Hz)
Linoleic acid	–CH_3_	0.883	t (7.0 Hz)
ω-9	–CH_3_	0.880	t (7.2 Hz)
SFA	–CH_3_	0.88-0.87	t (6.9 Hz)

**Table 10:** Longitudinal relaxation times T_1_ of proton nuclei of acyl chains of olive oil triacylglycerides. Reprinted, with permission, from [20]. Copyright 1999, Elsevier Science B.V.

^1^H Resonances	Chemical Shift (ppm)	T_1_ (s)
Glycerol residue		
1(3)	4.32	0.42
1′(3′)	4.17	0.44
2	5.26	0.79
Acyl residues		
2	2.30	0.53
3	1.57	0.73
4–7	1.25	0.86
8	2.00	1.06
9, 10	5.34	1.26
11	2.76	1.30
18	0.87	1.51

**Table 11:** ^13^C-NMR peak assignments of the carboxyl and the ester carbonyl region of UFAs in CDCl_3_.

Compound	Carbon	Functional Group	δ (ppm)
(9-*cis*, 11-*trans*)-CLA, (9-*trans*, 11-*cis*)-CLA, (11-*cis*, 13-*trans*)-CLA, (10-*trans*, 12-*cis*)-CLA	C1	OOC–CH_2_-	180.09
(8-*trans*, 10-*cis*)-CLA	C1	OOC–CH_2_-	180.05
(9-*cis*, 11-*cis*)-CLA	C1	OOC–CH_2_-	179.98
(9-*trans*, 11-*trans*)-CLA	C1	OOC–CH_2_-	179.86
FFA	C1	OOC–CH_2_-	178.04
Unsaturated FA in *sn*-1,3 of DAG	C1	–CH_2_–OOC–CH_2_-	173.32
FA in *sn*-1 (*sn*-3) of 1,2 (2,3) DAG	C1	–CH_2_–OOC–CH_2_-	173.18
FA in *sn*-2 of 1,2 (2,3) DAG	C1	–CH–OOC–CH_2_-	173.08
Unsaturated FA in *sn*-1,3 of TAG	C1	–CH_2_–OOC–CH_2_-	172.70
Butyric acid in *sn*-1,3 of TAG	C1	–CH_2_–OOC–CH_2_-	172.60
Unsaturated FA in *sn*-2 of TAG	C1	–CH–OOC–CH_2_-	172.38

**Table 12:** NOE and T_1_ values of carbonyl carbons of triglycerides. Reprinted, with permission, from [20]. Copyright 1999, Elsevier Science B.V.

Carbon Atom	δ (ppm) ^a^	NOE (1 + η)	Τ_1_ (s)
C1 *sn*-1(3)-Tripalmitin	173.10	1.77	5.6
Triolein	173.07	1.781	5.6
Trilinolein	173.06	1.73	5.4
C1 *sn*-2-Tripalmitin	172.70	1.74	3.9
Triolein	172.67	1.67	4.5
Trilinolein	172.66	1.68	4.4

^a^ Chemical shifts are referenced with respect to TMS = 0 ppm.

**Table 13:** ^13^C-NMR peak assignments of the olefinic carbons of some representative UFAs in CDCl_3_.

Compound	Carbon	Functional Group	δ (ppm)
Caproleic acid	C9	–CH=CH_2_	138.70
(9-*cis*, 11-*trans*)-CLA	C12	–CH=CH	135.80
(11-*cis*, 13-*trans*)-CLA	C14	–CH=CH	134.66
(10-*trans*, 12-*cis*)-CLA	C10	–CH=CH	134.58
(9-*trans*, 11-*cis*)-CLA	C9	–CH=CH	134.51
(8-*trans*, 10-*cis*)-CLA	C8	–CH=CH	134.32
(9-*trans*, 11*trans*)-CLA	C12	–CH=CH	132.53
(9-*trans*, 11-*trans*)-CLA	C9	–CH=CH	132.21
(9-*cis*, 11-*cis*)-CLA	C12	–CH=CH	132.19
(9-*cis*, 11-*cis*)-CLA	C9	–CH=CH	131.87
All ω-3 FA	ω3	–CH=CH	131.66
(9-*trans*, 11-*trans*)-CLA	C10	–CH=CH	130.46
(9-*trans*, 11*trans*)-CLA	C11	–CH=CH	130.30
(8-*trans*, 10-*cis*)-CLA	C11	–CH=CH	130.27
(9-*trans*, 11-*cis*)-CLA	C12	–CH=CH	130.17
(10-*trans*, 12-*cis*)-CLA	C13	–CH=CH	130.16
(11-*cis*, 13-*trans*)-CLA	C11	–CH=CH	130.04
Linoleic acid, Linolenic acid	C13, C9	–CH=CH	129.89
(9-*cis*, 11-*trans*)-CLA	C9	–CH=CH	129.89
Linoleic acid	C9	–CH=CH	129.51–129.49
(9-*cis*, 11-*trans*)-CLA	C10	–CH=CH	128.73
(11-*cis*, 13-*trans*)-CLA	C12	–CH=CH	128.66
(10-*trans*, 12-*cis*)-CLA	C12	–CH=CH	128.60
(9-*trans*, 11-*cis*)-CLA	C11	–CH=CH	128.57
(8-*trans*, 10-*cis*)-CLA	C10	–CH=CH	128.54
Linolenic acid	C13, C12	–CH=CH	127.97–127.92
Linoleic acid	C10	–CH=CH	127.77–127.76
Linoleic acid	C12	–CH=CH	127.59–127.58
Linolenic acid	C10	–CH=CH	127.46–127.44
All ω-3 FA	ω4	–CH=CH	126.77
(8-*trans*, 10-*cis*)-CLA	C9	–CH=CH	125.83
(9-*trans*, 11-*cis*)-CLA	C10	–CH=CH	125.72
(10-*trans*, 12-*cis*)-CLA	C11	–CH=CH	125.70
(11-*cis*, 13-*trans*)-CLA	C13	–CH=CH	125.65
(9-*cis*, 11-*trans*)-CLA	C11	–CH=CH	125.58
(9-*cis*, 11-*cis*)-CLA	C10	–CH=CH	123.72
(9-*cis*, 11-*cis*)-CLA	C11	–CH=CH	123.55
Caproleic acid	C10	–CH=CH_2_	114.05

**Table 14:** ^13^C chemical shift values of olefinic carbons of α-eleostearic acid, punicic acid, and β-eleostearic acid (ppm) in CDCl_3_. Reprinted, with permission, from [58].

Carbon Notation	β-eleostearic Acid: (*trans*-9, *trans*-11, *trans*-13) 18:3	Punicic Acid: (*cis*-9, *trans*-11, *cis*-13) 18:3	α-eleostearic Acid: (*cis*-9, *trans*-11, *trans*-13) 18:3
C-9	134.46	132.69	131.75
C-10	130.87	128.82	128.72
C-11	130.51	127.94	132.83
C-12	130.41	127.79	126.00
C-13	130.73	128.71	130.53
C-14	134.23	132.46	135.17

**Table 15:** ^13^C chemical shift increments (ppm) of the olefinic carbons in several positional CLA isomers. Reprinted, with permission, from [68].

Δδ	7-*cis*, 9-*trans*	8-*cis*, 10-*trans*	9-*cis*, 11-*trans*	10-*cis*, 12-*trans*	11-*cis*, 13-*trans*
C_1ol_–C_2ol_	0.45	0.83	1.07	1.22	1.30
C_4ol_–C_3ol_	9.43	9.25	9.14	9.06	8.93

**Table 16:** ^13^C-NMR peak assignments of the carbons of the glycerol moiety in CDCl_3_.

Compound	Carbon	Functional Group	δ (ppm)
Glycerol in 1,2/2,3 DAG		–CH–OOC–	71.85
Glycerol in TAG		–CH–OOC–	68.72
Glycerol in 1,3 DAG		HO-CH–(CH_2_)_2_	67.81
Glycerol in 1,3 DAG		–CH_2_–OOC–	64.75
Glycerol in 1,2/2,3 DAG		HO–CH_2_–CH–	62.02
Glycerol in TAG		–CH_2_–OOC–	61.83
Glycerol in 1,2 DAG		–CH_2_–OOC–	60.83

**Table 17:** ^13^C-NMR peak assignment of the aliphatic carbons of lipids in CDCl_3_.

Compound	Carbon	Functional Group	δ (ppm)
Butyric acid	C2	–OOC–CH_2_–CH_2_–	35.62
(9-*trans*, 11-*cis*)-CLA	C2	–OOC–CH_2_–CH_2_–	34.10
(9-*cis*, 11-*cis*)-CLA, (11-*cis*, 13-*trans*)-CLA, (10-*trans*, 12-*cis*)-CLA	C2	–OOC–CH_2_–CH_2_–	34.06
(9-*cis*, 11-*trans*)-CLA	C2	–OOC–CH_2_–CH_2_–	34.05
(18-*trans*, 10-*cis*)-CLA	C2	–OOC–CH_2_–CH_2_–	34.03
(9-*trans*, 11-*tans*)-CLA	C2	–OOC–CH_2_–CH_2_–	34.00
All FA except butyric in *sn*-2 of TAG	C2	–OOC–CH_2_–CH_2_–	33.92
All FA except butyric in *sn*-1,3 of TAG	C2	–OOC–CH_2_–CH_2_–	33.76
(9-*cis*, 11-*trans*)-CLA	C13	–CH_2_–CH=CH–	32.90
(10-*trans*, 12-*cis*)-CLA	C9	–CH_2_–CH=CH–	32.87
(9-*trans*, 11-*cis*)-CLA	C8	–CH_2_–CH=CH–	32.86
(8-*trans*, 10-*cis*)-CLA	C7	–CH_2_–CH=CH–	32.78
(9-*trans*, 11-*trans*)-CLA	C13	–CH_2_–CH=CH–	32.63
(11-*cis*, 13-*trans*)-CLA	C15	–CH_2_–CH=CH–	32.57
(9-*trans*, 11-*trans*)-CLA	C8	–CH_2_–CH=CH–	32.56
(8-*trans*, 10-*cis*)-CLA	C16	–CH_2_–CH_2_–CH_3_	31.87
(9-*trans*, 11-*trans*)-CLA, (9-*trans*, 11-*cis*)-CLA	C16	–CH_2_–CH_2_–CH_3_	31.77
(9-*cis*, 11-*cis*)-CLA, (9-*cis*, 11-*trans*)-CLA	C16	–CH_2_–CH_2_–CH_3_	31.76
(11-*cis*, 13-*trans*)-CLA	C16	–CH_2_–CH_2_–CH_3_	31.59
(10-*trans*, 12-*cis*)-CLA	C16	–CH_2_–CH_2_–CH_3_	31.50
Linoleic acid	ω3	–CH_2_–CH_2_–CH_3_	31.32
(8-*trans*, 10-*cis*)-CLA	C13	–CH_2_–	29.76
(11-*cis*, 13-*trans*)-CLA	C9	–CH_2_–	29.73
(9-*trans*, 11-*cis*)-CLA	C14	–CH_2_–	29.73
(9-*cis*, 11-*cis*)-CLA	C14	–CH_2_–	29.64
(9-*cis*, 11-*cis*)-CLA	C7	–CH_2_–	29.57
All FA		–(CH_2_)_n_–	29.56–28.73
(10-*trans*, 12-*cis*)-CLA	C15	–CH_2_–	29.44
(9-*trans*, 11-*trans*)-CLA	C14	–CH_2_–	29.42
(9-*cis*, 11-*trans*)-CLA	C14	–CH_2_–	29.40
(10-*trans*, 12-*cis*)-CLA	C8	–CH_2_–	29.39
(9-*trans*, 11-*trans*)-CLA	C7	–CH_2_–	29.36
(10-*trans*, 12-*cis*)-CLA	C5, C6, C7	–CH_2_–	29.16–29.29
(9-*trans*, 11-*cis*)-CLA	C5, C6, C7	–CH_2_–	29.13–29.45
(9-*cis*, 11-*trans*)-CLA	C5, C6, C7	–CH_2_–	29.12–29.67
(11-*cis*, 13-*trans*)-CLA	C4, C5, C6, C7, C8	–CH_2_–	29.07–29.45
(10-*trans*, 12-*cis*)-CLA	C4	–CH_2_–	29.06
(9-*cis*, 11-*trans*)-CLA	C4	–CH_2_–	29.03
(9-*cis*, 11-*cis*)-CLA	C4, C5, C6, C15	–CH_2_–	28.99–29.11
(9-*trans*, 11-*cis*)-CLA	C4, C15	–CH_2_–	28.97
(9-*cis*, 11-*trans*)-CLA	C15	–CH_2_–	28.93
(9-*trans*, 11-*trans*)-CLA	C4, C5, C6, C15	–CH_2_–	28.92–29.10
(8-*trans*, 10-*cis*)-CLA	C4, C5, C6, C14, C15	–CH_2_–	28.81–29.25
(9-*trans*, 11-*cis*)-CLA	C13	–CH_2_–CH=CH–	27.72
(8-*trans*, 10-*cis*)-CLA	C12	–CH_2_–CH=CH–	27.71
(11-*cis*, 13-*trans*)-CLA	C10	–CH_2_–CH=CH–	27.69
(10-*trans*, 12-*cis*)-CLA	C14	–CH_2_–CH=CH–	27.68
(9-*cis*, 11-*trans*)-CLA	C8	–CH_2_–CH=CH–	27.65
(9-*cis*, 11-*cis*)-CLA	C8	–CH_2_–CH=CH–	27.52
(9-*cis*, 11-*cis*)-CLA	C13	–CH_2_–CH=CH–	27.43
Unsaturated FA		–CH_2_–CH=CH–	26.91–26.88
PUFA		–CH=CH–CH_2_–CH=CH–	25.34
(9-*trans*, 11-*cis*)-CLA	C3	–CH_2_–	24.95
(11-*cis*, 13-*trans*)-CLA	C3	–CH_2_–	24.69
(9-*cis*, 11-*cis*)-CLA	C3	–CH_2_–	24.68
(9-*trans*, 11-*trans*)-CLA, (9-*cis*, 11-*trans*)-CLA, (10-*trans*,12-*cis*)-CLA	C3	–CH_2_–	24.67
(8-*trans*, 10-*cis*)-CLA	C3	–CH_2_–	24.63
All FA except butyric		–OOC–CH_2_–CH_2_–	24.58
(8-*trans*, 10-*cis*)-CLA	C17	–CH_2_–CH_3_	22.68
(9-*trans*, 11-*cis*)-CLA	C17	–CH_2_–CH_3_	22.65
(9-*cis*, 11-*cis*)-CLA	C17	–CH_2_–CH_3_	22.64
(9-*trans*, 11-*trans*)-CLA, (9-*cis*, 11-*trans*)-CLA	C17	–CH_2_–CH_3_	22.63
(10-*trans*, 12-*cis*)-CLA	C17	–CH_2_–CH_3_	22.57
All FA except ω-3	ω2	–CH_2_–CH_3_	22.48
(11-*cis*, 13-*trans*)-CLA	C17	–CH_2_–CH_3_	22.29
ω-3	ω2	–CH_2_–CH_3_	20.13
Butyric acid	C3	–CH_2_–CH_3_	18.10
(9-*trans*, 11-*cis*)-CLA	C18	–CH_3_	14.12
ω-3	ω1	–CH_3_	14.11
(8-*trans*, 10-*cis*)-CLA	C18	–CH_3_	14.11
(9-*trans*, 11-*trans*)-CLA, (9-*cis*, 11-*trans*)-CLA	C18	–CH_3_	14.10
(9-*cis*, 11-*cis*)-CLA	C18	–CH_3_	14.09
(10-*trans*, 12-*cis*)-CLA	C18	–CH_3_	14.06
(11-*cis*, 13-*trans*)-CLA	C18	–CH_3_	13.95
Saturated	ω-1	–CH_3_	13.96
ω-9	ω-1	–CH_3_	13.95
ω-7	ω-1	–CH_3_	13.94
ω-6	ω-1	–CH_3_	13.91

**Table 18:** ^1^H-NMR peak assignments of the (9-*cis*, 11-*trans*) 18:2 CLA in CDCl_3_.

Functional Group	Atom	δ (ppm)	Multiplicity
HOOC–	H1	10.43	^a^
–CH=	H11	6.28	dd
–CH=	H10	5.93	t
–CH=	H12	5.65	m
–CH=	H9	5.33	m
–CH_2_-COOH	H2	2.35	t
–CH_2_–CH=CH–	H8, H13	2.13	m
–CH_2_-CH_2_–COOH	H3	1.65	m
–CH_2_–	H4–H7, H14–H17	1.25–1.35	m
–CH_3_	H18	0.87	t

^a^ Very broad resonance due to ^1^H exchange with traces of H_2_O in the organic solvent.

**Table 19:** ^1^H and ^13^C peak assignments of the (9-*cis*, 11-*trans*) 18:2 CLA isomer in CDCl_3_ (solution conditions the same as in Figure 16).

^1^H	δ_Η_ (ppm)	^13^C	δ_c_ (ppm)
H1	10.43	C1	179.0
H2	2.35	C2	34.2
H3	1.65	C3	24.9
H4	1.31	C4	29.5
H5	1.32	C5	29.4
H6	1.34	C6	29.6
H7	1.36	C7	29.7
H8	2.1	C8	28.1
H9	5.33	C9	129.6
H10	5.93	C10	128.4
H11	6.28	C11	125.3
H12	5.65	C12	134.8
H13	2.08	C13	33.4
H14	1.30	C14	29.1
H15	1.28	C15	28.9
H16	1.27	C16	32.0
H17	1.26	C17	23.1
H18	0.87	C18	14.3

**Table 20:** ω-3 content (%) of α-linolenic acid (ALA) in seed oils. Reprinted with permission from [111].

Common Name	Linnaean Name	ω-3 (%)
Chia	*Salvia hispanica*	64
Kiwifruit	*Actinidia chinensis*	62
Perilla	*Perilla frutescens*	58
Flax	*Linum usitatissimum*	55
Lingonberry	*Vaccinium vitis-idaea*	49
Camelina	*Camelina sativa*	36
Purslane	*Portulaca oleracea*	35
Black Raspberry	*Rubus occidentalis*	33

**Table 21:** Results obtained using 1D TOCSY NMR, conventional ^1^H-NMR and GC-MS methods for the determination of the caproleic acid in two milk samples. Reprinted, with permission, from [78]. Copyright 2015, American Chemical Society.

Sample	Analyte/Units	1D TOCSY ^a^	^1^H-NMR ^a^	Relative Deviation ^b^ (%)	GC-MS	Relative Deviation ^c^ (%)
1	Caproleic acid/mM	1.02 ± 0.03	0.97 ± 0.02 ^a^	5.15		
	Caproleic acid/% of the lipid fraction		0.26 ± 0.01		0.28	−7.14
2	Caproleic acid/mM	1.92 ± 0.06	1.83 ± 0.06 ^a^	1.75		
	% of the lipid fraction		0.74 ± 0.01		0.68	8.82

^a^ Results are expressed as mM of caproleic acid (in tube; standard deviation (*n* = 3)). ^b^ Results are expressed as 100 × [(1D TOCSY_value_) − (1D ^1^H NMR_value_)]/(1D ^1^H NMR_value_). ^c^ Results are expressed as 100 × [(NMR_value_) − (GC-MS_value_)]/(GC-MS_value_).

**Table 22:** ^1^H-NMR chemical shifts and spin multiplicities of oxidation products of edible lipids. Adopted, with permission, from [26]. Copyright 2014, Institute of Food Technologists.

Signal	Chemical Shift (ppm)	Multiplicity	Functional Group
**Primary Oxidation Compounds**			
–CH=CH–CH=CH–	6.58	dddd	(*Z*,*E*)-conjugated double
	6.00	ddtd	bonds associated with
	5.56	ddm	hydroperoxides (OOH)
	5.51	dtm	
–CH=CH–CH=CH–	6.45	ddd	(*Z*,*E*)-conjugated double
	5.94	dd	bonds associated with
	5.64	dd	hydroxides (OH)
	5.40	ddt	
–CH=CH–CH=CH–	6.27	ddm	(*E*,*E*)-conjugated double
	6.06	ddtd	bonds associated with
	5.76	dtm	hydroperoxides (OOH)
	5.47	ddm	
–CHOOH–CH=CH–	5.72	m	Double bond associated with
			hydroperoxides (OOH)
–OOH	8.3 to 8.9	-	Hydroperoxide group
**Secondary or Further Oxidation Compounds**			
**Aldehydes**			
–CHO	9.49	d	(*E*)-2-alkenals
–CHO	9.52	d	(*E*,*E*)-2,4-alkadienals
–CHO	9.55	d	4,5-epoxy-2-alkenals
–CHO	9.57	d	4-hydroxy-(*E*)-2-alkenals
–CHO	9.58	d	4-hydroperoxy-(*E*)-2-alkenals
–CHO	9.60	d	(*Z*,*E*)-2,4-alkadienals
–CHO	9.75	t	*n*-alkanals
–CHO	9.78	t	4-oxo-alkanals
–CHO	9.79	t	*n*-alkanals of low molecular
			weight (ethanal and propanal)
**Alcohols**			
–CHOH–CHOH–	3.43	m	9,10-dihydroxy-12-octadecenoate
			(leukotoxindiol)
–CHOH–	3.54–3.59	m	secondary alcohols
–CH2OH–	3.62	t	primary alcohols
**Epoxides**			
–CHOHC–	2.63	m	(*E*)-9,10-epoxystearate
–CHOHC–	2.88	m	(*Z*)-9,10-epoxystearate
–CHOHC–	2.90	m	9,10-epoxy-octadecanoate;
			9,10-epoxy-12-
			octadecenoate
			(leukotoxin); 12,13-epoxy-
			9-octadecenoate
			(isoleukotoxin)
–CHOHC–CHOHC–	2.90	m	9,10–12,13-diepoxy
			octadecanoate
–CHOHC–CH_2_–CHOHC–	3.10	m	9,10–12,13-diepoxy
			octadecanoate
**Ketones and Unidentified**			
O=C*<*CH=CH–	6.08	dt	Double bond conjugated with a keto group
	6.82	m	
			
Unidentified	7.50	-	Unidentified
Unidentified	8.10	-	Unidentified

d, doublet; t, triplet; m, multiplet.
